# Reaction Studies on the Conversion of Aryl Methyl Halides to Aryl Carbonyl Compounds Using Ph_4_PBr/EDTA/CaF_2_ as a Catalytic Oxidation System

**DOI:** 10.3390/molecules31203742

**Published:** 2026-10-18

**Authors:** Xianwei Xu, Sida Li, Xincheng Liu, Jun You, Yanchao Yu, Yifang Liang, Wenju Wu

**Affiliations:** Key Laboratory of Green Chemical Engineering and Technology of College of Heilongjiang Province, College of Materials Science and Chemical Engineering, Harbin University of Science and Technology, Harbin 150080, China; xuxianwei34@163.com (X.X.); lisida1101@126.com (S.L.); liuxincheng2001@163.com (X.L.); yychao136@163.com (Y.Y.); liangyifang960701@163.com (Y.L.)

**Keywords:** catalytic oxidation, tetraphenylphosphonium bromide, arylmethyl halides, aromatic carbonyl compounds

## Abstract

We have developed an efficient protocol for the selective oxidation of arylmethyl halides to aromatic carbonyl compounds. This transformation employs tetraphenylphosphonium bromide (Ph_4_PBr) as both an oxidant and phase-transfer catalyst (PTC), in combination with EDTA and CaF_2_ as cocatalysts. Under mild reaction conditions, the method enables highly selective conversion of C-X bonds (X = Cl, Br) to carbonyl (C = O) functionalities. A diverse range of aromatic carbonyl products—including aldehydes, ketones, *α*-diketones, *α*-keto esters, and anhydrides—were obtained in good to excellent yields (50~95%).

## 1. Introduction

Oxidation reactions play a fundamental role in organic synthesis, enabling the preparation of alcohols, acids, ketones, aldehydes, esters, and other key intermediates for pharmaceuticals, agrochemicals, and fine chemicals. Due to their broad applicability, they remain one of the most studied transformations in organic chemistry. Early approaches relied on direct C–H oxidation, often requiring noble metal catalysts to achieve sufficient selectivity and yield [1,2]. However, recent efforts have focused on developing sustainable alternatives, including organocatalytic systems, to minimize metal dependence and suppress overoxidation [3,4,5].

**Scheme 1:**
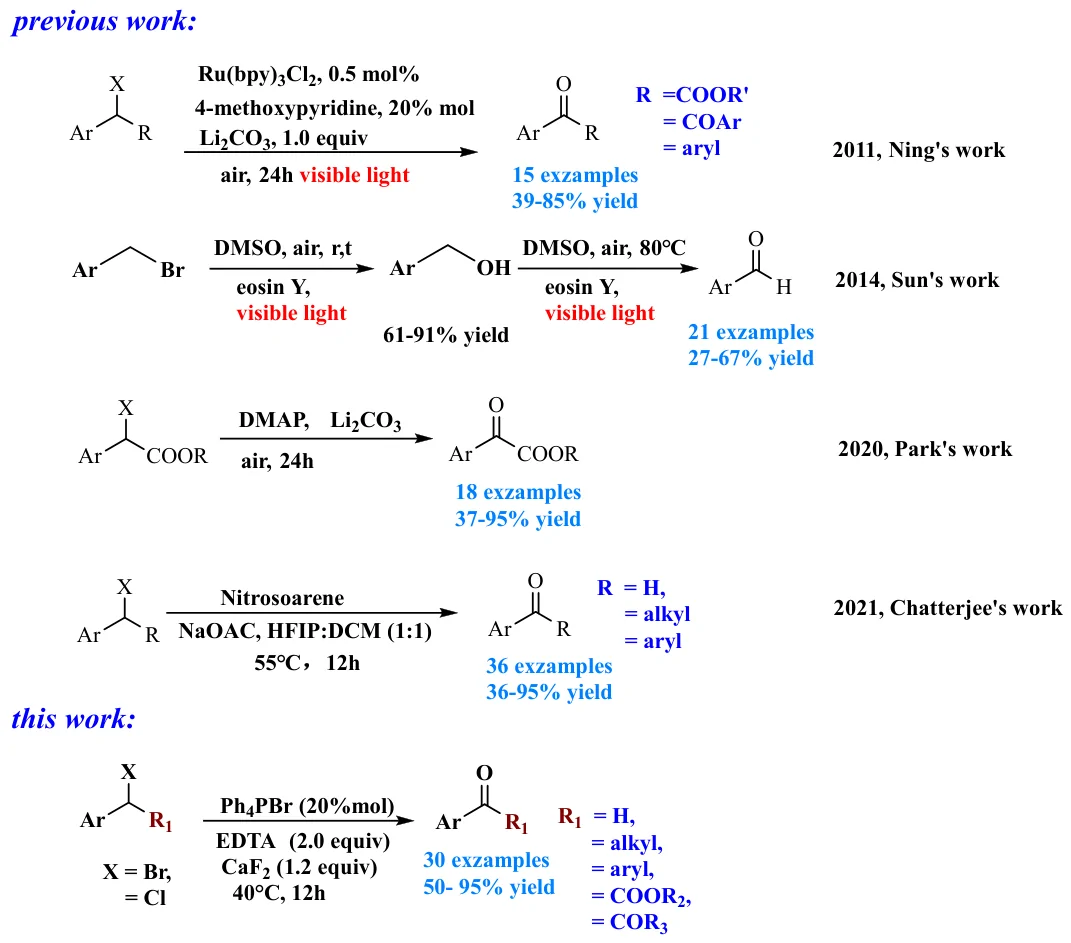
Comparison between the results of Ning et al. and this work.

An alternative strategy involves the oxidation of arylmethyl halides to aromatic carbonyl compounds [6,7]. Classical methods such as the Hass–Bender [8], Sommelet [9], Kornblum [10], and Kröhnke [11] oxidations have been widely employed, yet they suffer from a few limitations, including byproduct formation, harsh conditions, reliance on precious metals, and narrow substrate scope [12]. Consequently, there is a growing demand for more practical, economical, and environmentally benign protocols [13]. Recent advances have introduced organocatalytic approaches for this transformation, offering improved sustainability and broader applicability (Scheme 1, previous work) [14,15,16,17].

Quaternary phosphine salts were often used as non-homogeneous PTCs due to their stability under neutral and acidic conditions [18], but in 2006, S. Aldridge found that Ph_4_PBr readily generates triphenylphosphine oxide (Ph_3_PO) under alkaline conditions [19]. Moreover, Jian-Qiu Zhang et al. revealed that pentavalent Ph_3_PO could be converted to trivalent phosphorus by eliminating a phenyl ring [20]. Since pentavalent phosphorus can be reduced to trivalent phosphorus, we aim to utilize the electron transfer process from P^(5+)^ to P^(3+)^ to establish a redox system using pentavalent phosphorus as the oxidant (Scheme 1, *this work*).

## 2. Results and Discussion

### 2.1. Synthesis of Aryl Carbonyl Compounds

**Scheme 5:**
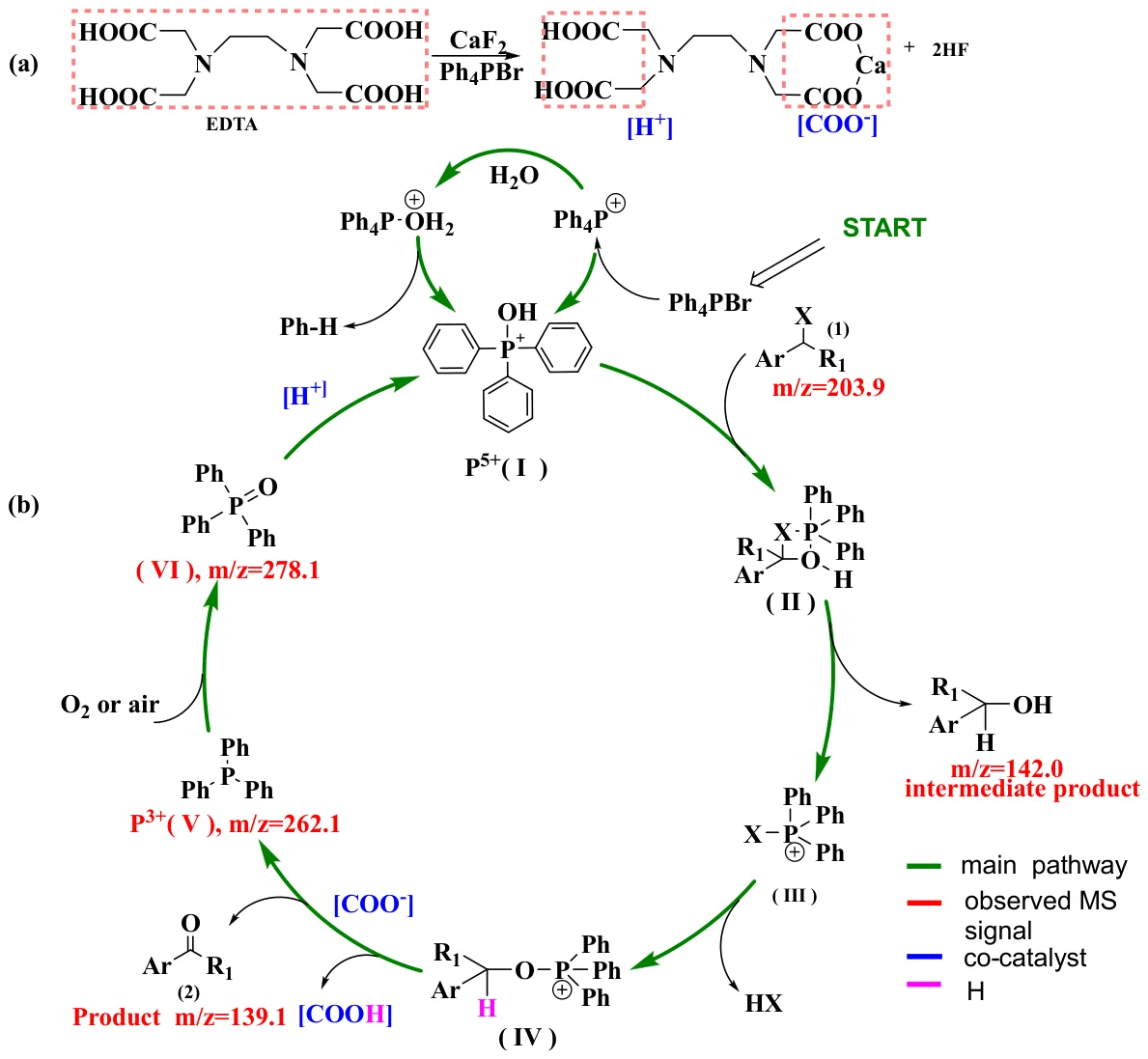
Speculate on possible reaction mechanisms.

To verify our idea, we studied the oxidation of benzyl bromide (**1a**) in an open environment at 40 °C (Table 1). The oxidation reaction cannot take place in the absence of pentavalent phosphorus salts (Table 1, entry 17). Comparative observations were made with different pentavalent phosphines (**A**~**D**, 20 mol%) as catalysts in the polar nonprotonic solvent DMF. From the comparisons (Table 1, entries 1–4), we observed that both EDTA and the base were essential for the reaction to take place. The yield was significantly reduced after changing TPPB (**D**) to tetrabutylphosphonium bromide (**A**) or butyltriphenylphosphonium bromide (**B**), even with the addition of EDTA (Table 1, entries 5~6), which was attributed to the fact that the alkyl chain of the quaternary phosphonium salt was not easily eliminated compared to the benzene ring; this was demonstrated in the report by Jian-Qiu Zhang (20), who experimentally argued that the alkyl chain was more stable in the quaternary phosphonium salt structure compared to the benzene ring. At the same time, Ph_3_PO (**C**) had a slower reaction rate (Table 1, entry 7), and this result reinforced our speculation about the two intermediates of this reaction, triphenyl-phosphine hydroxide (I) and four-membered ring (II, Scheme 5).

We also screened the types of bases (Table 1, entries 4, 8~10), and calcium fluoride ultimately proved to be the most suitable base, probably due to the chelation of calcium fluoride by EDTA forming COO^−1^ that is analogous to Perkin reaction, exhibiting a higher tendency to deprotonate the benzylic H. For hydrogen bond donors, we examined hexafluoroisopropanol (HFIP), EDTA and 18-crown-6 ether. When EDTA was replaced with 18-crown-6, although 40% benzaldehyde was obtained, benzoic acid was also generated in 50% yield (Table 1, entry 11), which meant that the EDTA system was more conducive to keeping the oxidation reaction at the aldehyde stage, whereas 18-crown-6 ether significantly reduced the selectivity of the reaction, resulting in a mixture of both the aldehyde and carboxylic acid. When HFIP was used as the hydrogen bond donor, the yield was also reduced (Table 1, entry 12) due to the weaker basicity of HFIP; it could not capture the H on the phenylmethyl C. When the reaction temperature was lowered from 40 °C to room temperature, the reaction yield was also reduced from 82% to 30% (Table 1, entry 13). At this point, the optimal conditions for Ph_4_PBr/EDTA/CaF_2_ as a catalytic oxidation system for this reaction had been determined.

We further optimized the loading of Ph_4_PBr. Reducing the loading from 20 mol% to 10 mol% led to an obvious decrease in reaction rate, with merely 50% yield at 24 h. A yield of 72% was obtained at 15 mol% loading, whereas increasing the loading to 25 mol% gave negligible yield improvement. Hence, 20 mol% was chosen as the optimum condition (Table 1, entries 14~16).

**Scheme 2:**
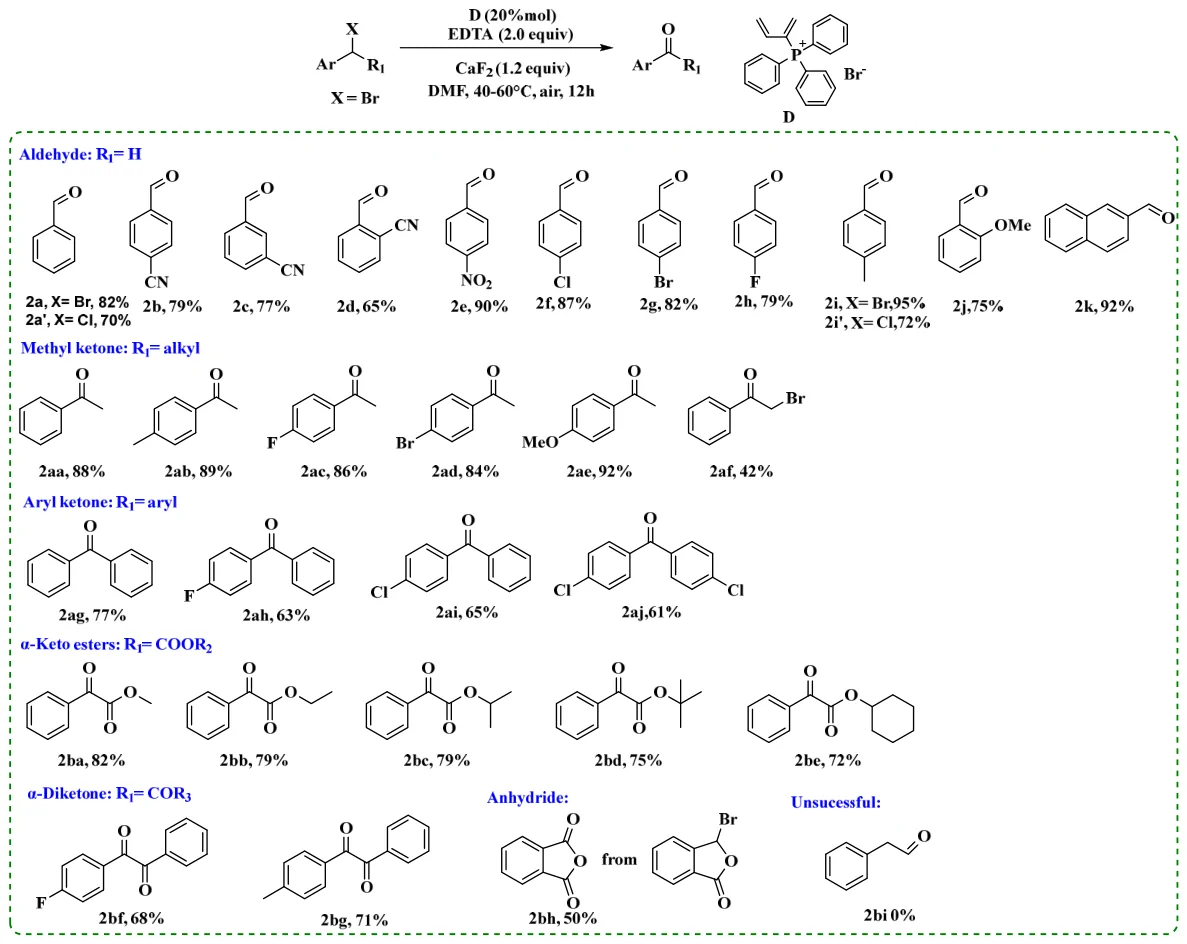
Transformation of arylmethyl bromides to aryl carbonyl compounds.

During the exploration of the substrate scope (Scheme 2), benzyl bromide generated the corresponding aldehydes (**2a**–**b, 2e**–**h**) in good to excellent yields (77–90%) when the benzene ring substituents of the arylmethyl bromide were CN, NO_2_, and halogens (F, Cl, and Br), respectively; the yields were slightly reduced when the electron-withdrawing group was in the neighboring position (**2d**, 65%), and the meta substituents did not have much effect on the oxidation reaction (**2c**, 77%). Electron-rich aryl methyl bromides such as Me and OMe were even higher in yield, up to 95% (**2i**). The fused *β*-bromomethyl naphthalene was also successfully oxidized to *β*-naphthaldehyde in 92% yield (**2k**). Benzyl chlorides could undergo this reaction as well, but the conversion was slightly reduced compared to the bromine substitutes (**2a’**, **2i’**). The method was also suitable for the preparation of aliphatic ketones and benzophenones (**2aa**–**2af**) in yields up to 42–92%.

As we could see from the above findings, the method was suitable for the preparation of aromatic aldehydes and aromatic ketones. In order to more deeply investigate whether the method was appropriate for other substrates, we added complex functional groups such as acyl groups and alkoxycarbonyl groups to the neighboring positions of phenylmethyl groups. The results showed that the method could oxidize C-Br bonds into C = O double bonds with high selectivity, and particularly tolerates sensitive groups such as ester groups in the molecule. In other words, by using this oxidation method, α-carbonylic acid esters, α-diketones and anhydrides can be prepared in yields of 50–82% (**2ba**–**2bh**). When bromine was located at the β-position of the benzene ring side chain, the oxidation can not occur (**2bi**), likely because the COO^−^ from EDTA/Ca was unable to attack the β-H (weaker acidity than α-H) during the oxidation process.

### 2.2. Oxidation Reaction Mechanism of α-Carbonyl Bromide

**Scheme 3:**
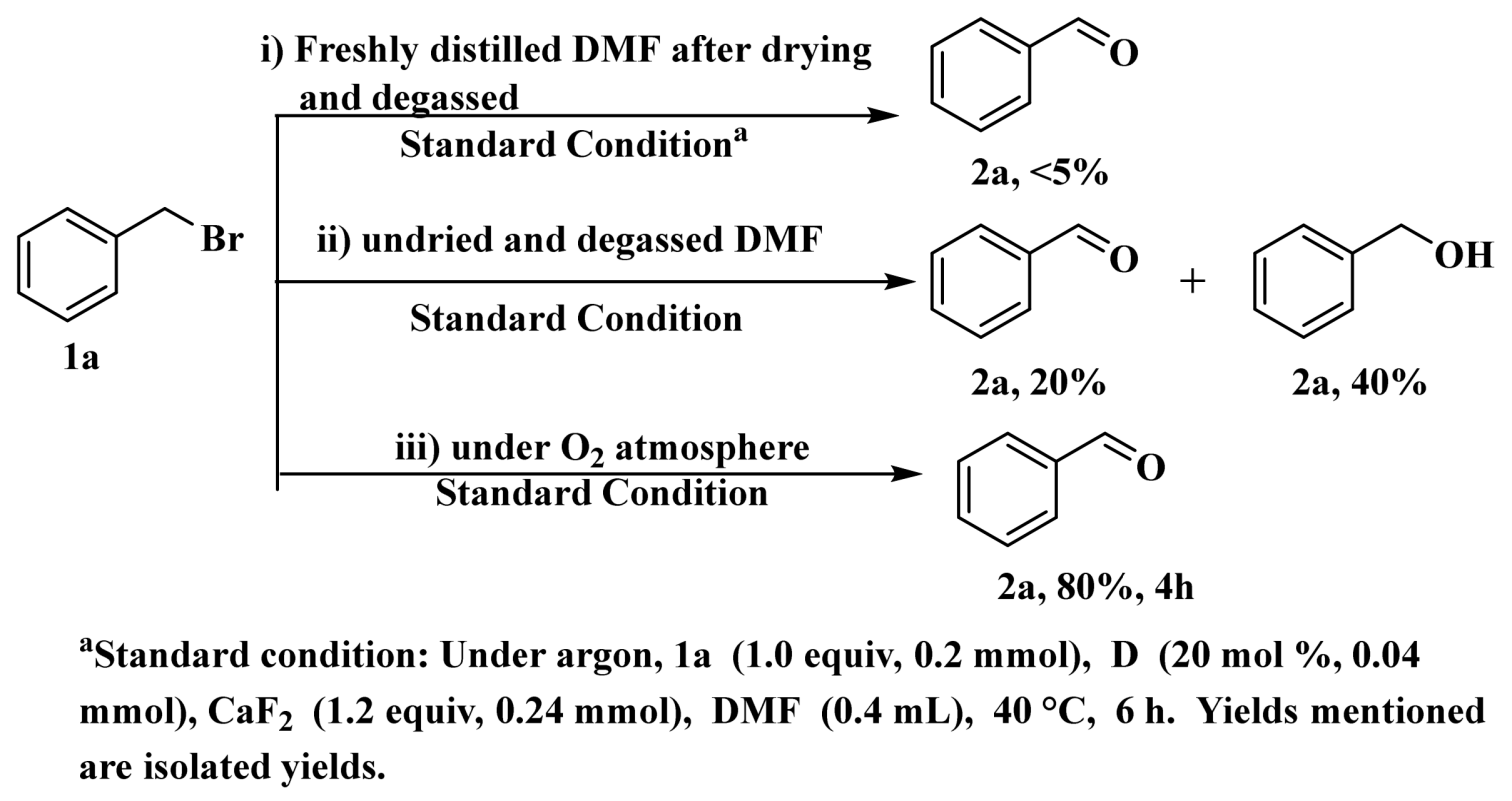
Effect of oxygen and water on the reactions.

To elucidate the reaction mechanism, we carried out a series of controlled experiments (Scheme 3) to investigate whether oxygen and water promote the reaction. As shown in Experiment i (Scheme 3), benzyl bromide barely produced benzaldehyde in strictly degassed and anhydrous DMF, indicating that the oxidation reaction cannot proceed under strictly anhydrous and anaerobic conditions. However, in degassed but non-anhydrous DMF, 20% benzaldehyde and 40% benzyl alcohol were generated, demonstrating that even in the absence of oxygen, C–X bonds can still be converted into C–O and C = O bonds in the presence of trace water, albeit at a very slow rate (Scheme 3, ii). Direct introduction of oxygen into the system markedly accelerated the reaction, affording an 80% aldehyde yield in just 4 h, which implies that the oxidant may require oxygen as a co-catalyst and another crucial oxidant to drive C-O bond formation. (Scheme 3, iii).

**Scheme 4:**
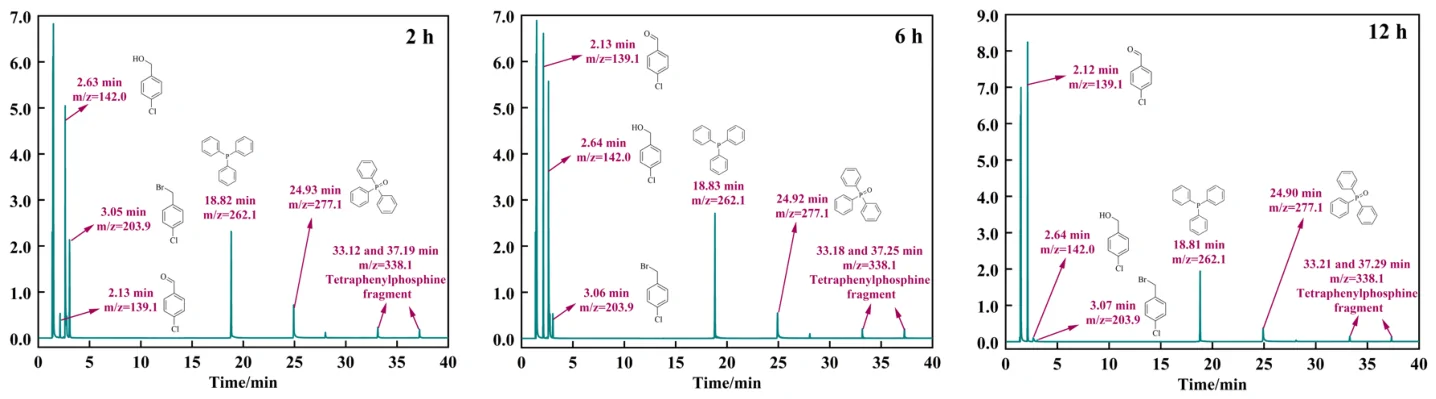
Exploring the reaction process by GC-MS.

During the experiment, the reaction was monitored using GC-MS. It was found that alcohol was the main intermediate in the initial reaction. As the reaction proceeded, the yield of aldehyde gradually increased, and finally, almost all the alcohol was completely converted to aldehyde. It was also found that Ph_3_P and Ph_3_PO were always present in the system, which verified part of our conjecture about the mechanism (see Scheme 4).

Based on control experiments and GC-MS monitoring, we proposed the following reaction mechanism. In this heterogeneous catalytic system, Ph_4_PBr not only acted as the PTC but also served as a key oxidant to drive the reaction. The reaction proceeds via the following steps:

(1) **EDTA-CaF_2_ Complexation:** Under Ph_4_PBr as the PTC, EDTA formed a complex with CaF_2_. Due to the 2.0: 1.2 molar ratio of EDTA to CaF_2_, uncomplexed EDTA acted as an H^+^ donor, while the EDTA-Ca complex provided COO^−^ for abstraction of benzylic hydrogen (Scheme 5a).

(2) **Oxidative Mechanism of Ph_4_PBr** (Scheme 5b): Ph_4_PBr underwent HBr elimination under acidic conditions, generating Ph_4_P^+^ (*m*/*z* = 339.1). With H_2_O participation, Ph_4_P^+^ further lost a phenyl ring to form the key intermediate (I). Intermediate (I) reacted with the benzyl halide substrate, forming a four-membered transition state (II) and rearranging to yield the alcohol product (*m*/*z* = 142.0) and Ph_3_P^+^X^−^ (III). Ph_3_P^+^X^−^ reacted with the alcohol, eliminating HX to form intermediate (IV). The COO^−^ from EDTA-Ca abstracted the benzylic H from IV, ultimately producing the carbonyl product **2** (*m*/*z* = 139.1) and Ph_3_P (V, *m*/*z* = 262.1). Here, the pentavalent phosphorus (Ph_4_PBr) was reduced to trivalent phosphorus (Ph_3_P), while the C-X bond was oxidized to a C = O bond (Scheme 5b). Notably, intermediate IV was also an intermediate formed by the substitution of alcohol with X^−^, which was exactly the intermediate of the classic Appel reaction. However, the Appel reaction is generally carried out with carbon tetrabromide as the solvent or under strongly acidic conditions such as oxalyl chloride. These conditions were vastly different from those in this paper. In addition, GC-MS monitoring of the reaction process verified that the starting bromide gradually decreased during the reaction and was almost fully consumed at the endpoint, so this reaction was not reversible.

(3) **Regeneration of Ph_3_P:** Ph_3_P (V) was reoxidized to Ph_3_PO (VI, *m*/*z* = 278.1) by air or oxygen, then converted back to intermediate (I) under H^+^, completing the catalytic cycle.

It was worth mentioning that when Ph_3_PO was used directly as the catalyst, the reaction rate dropped significantly (Table 1, Entry 7, 12 h, 37% yield), highlighting the essential roles of Ph_4_PBr’s phase-transfer ability and synergistic H^+^/[COO^−^] supply.

It should be noted that the proposed mechanism is only speculative. In particular, phosphorus-containing intermediates II, IIL, and IV are merely plausible structures deduced from the available evidence, and their actual structures remain to be further verified.

## 3. Experimental Section

### 3.1. Instrumentation and Materials

Unless otherwise stated, all reactions were carried out in flame-dried glassware under aerobic conditions. Liquids and solutions were transferred using syringes. All solvents were dried and purified according to standard procedures. Industrial-grade solvents (ethyl acetate and petroleum ether) used for extraction or chromatography were distilled before use. CDCl_3_ was stored over 4 Å molecular sieves. Chemicals were purchased from Sigma-Aldrich Co. LLC (St. Louis, MO, USA), Tokyo Chemical Industry (Tokyo, Japan), Alfa Aesar (Ward Hill, MA, USA), and Sisco Research Laboratories (Mumbai, India) and used without further purification. Tetraphenylphosphonium bromide was purchased from Annaiji (Shang Hai, China) and stored at room temperature. Analytical thin-layer chromatography (TLC) was performed on silica gel 60 F254 plates from Merck Chemicals (Shanghai) Co., Ltd. (Shang Hai, China). Flash column chromatography was carried out on Merck silica gel 60 (40–63 μm, 230–400 mesh, ASTM) using the specified solvents. ^1^H and ^13^C NMR spectra were recorded in CDCl_3_ unless otherwise stated, using a AVANCE-300 spectrometer from Bruker BioSpin GmbH (Silberstreifen 4, 76287 Rheinstetten, Germany), Chemical shifts are reported in parts per million (ppm) relative to the residual solvent signal (CDCl_3_: δ = 7.26 ppm for ^1^H NMR and δ = 77.0 ppm for ^13^C NMR; DMSO: δ = 2.51 ppm for ^1^H NMR and δ = 40.5 ppm for ^13^C NMR). Data are reported as follows: chemical shift, multiplicity (br = broad singlet, s = singlet, d = doublet, dd = doublet of doublets, t = triplet, q = quartet, sept = septet, m = multiplet), coupling constant (Hz), and integration. Agilent 7890B gas chromatograph coupled with a 5977A mass selective detector (Agilent Technologies, Inc., Santa Clara, CA, USA) was used for GC-MS analysis.. Detection methods (gasification chamber: 300 °C; auxiliary heating zone: 280 °C; column box: 150 °C, hold for five minutes, 5 °C/min ramp rate to 250 °C, hold for another 15 min; MS ion source temperature: 230 °C; MS quadrupole temperature: 150 °C).

### 3.2. General Procedure for the Substrate Synthesis of (**1ba**–**1be**)


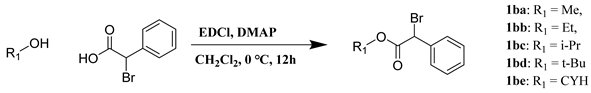


A 100 mL three-neck flask equipped with a magnetic stir bar was charged with fatty alcohol (20–30 mmol) and dissolved in 20 mL of refined dichloromethane. Subsequently, 1-(3-dimethylaminopropyl)-3-ethylcarbodiimide hydrochloride (EDCI) (15 mmol) and 4-dimethylaminopyridine (DMAP) (12 mmol) were added to the flask. Under nitrogen protection, 2-bromophenylacetic acid (10 mmol) was slowly introduced dropwise, with the addition rate carefully controlled to ensure the system temperature did not exceed 0 °C. The consumption of the starting material (2-bromophenylacetic acid) was monitored by thin-layer chromatography (TLC). After 12 h of reaction, the starting material spot had largely disappeared. Upon completion, the reaction was quenched by adding 20 mL of saturated sodium bicarbonate solution. The mixture was then extracted with dichloromethane and saturated brine 2–3 times. The combined organic layers were dried over anhydrous magnesium sulfate, filtered, and concentrated under reduced pressure to obtain the crude product. The crude product was purified by silica gel column chromatography using a mobile phase of petroleum ether: ethyl acetate = 3:1, yielding a white solid.

#### 3.2.1. Methyl 2-phenyl-2-bromoacetate (**1ba**) [16]

The compound was purified by flash column chromatography (ethyl acetate/hexanes: 1/20). Colorless liquid (90% yield). ^1^H NMR (300 MHz, CDCl_3_) δ 7.59–7.47 (m, 2H), 7.40 (dd, *J* = 5.0, 2.0 Hz, 3H), 5.39 (s, 1H), 3.80 (s, 3H). ^13^C NMR (75 MHz, CDCl_3_) δ 168.9, 135.7, 129.3, 128.8, 127.9, 58.9, 53.3.

#### 3.2.2. Ethyl 2-phenyl-2-bromoacetate (**1bb**) [16]

The compound was purified by flash column chromatography (ethyl acetate/hexanes: 1/20). Colorless liquid (89% yield). ^1^H NMR (300 MHz, DMSO) δ 7.57–7.47 (m, 2H), 7.39 (dd, *J* = 5.4, 2.2 Hz, 3H), 5.26 (s, 1H), 1.45 (s, 9H). ^13^C NMR (75 MHz, DMSO) δ 167.3, 136.4, 129.0, 128.7, 127.8, 83.1, 60.0, 27.8.

#### 3.2.3. 2-phenyl 2-phenyl-2-bromoacetate (**1bc**) [16]

The compound was purified by flash column chromatography (ethyl acetate/hexanes: 1/20). Colorless liquid (82% yield). ^1^H NMR (300 MHz, DMSO-*d_6_*) δ 7.58–7.46 (m, 2H), 7.46–7.32 (m, 3H), 5.85 (s, 1H), 4.97 (p, *J* = 6.2 Hz, 1H), 1.21 (d, *J* = 6.3 Hz, 3H), 1.11 (d, *J* = 6.2 Hz, 3H). ^13^C NMR (75 MHz, DMSO-*d*_6_) δ 167.8, 136.7, 129.6, 129.2, 128.4, 70.4, 59.2, 21.7.

#### 3.2.4. Tert-butyl 2-bromo-2-phenylacetate (**1bd**) [21]

The compound was purified by flash column chromatography (ethyl acetate/hexanes: 1/20). Colorless liquid (93% yield). ^1^H NMR (300 MHz, CDCl_3_) δ 7.57–7.47 (m, 2H), 7.39 (dd, *J* = 5.4, 2.2 Hz, 3H), 5.26 (s, 1H), 1.45 (s, 9H). ^13^C NMR (75 MHz, CDCl_3_) δ 167.3, 136.4, 129.0, 128.7, 127.8, 83.1, 60.0, 27.7.

#### 3.2.5. Eyclohexylester 2-bromo-2-phenylacetate (**1be**) [22]

The compound was purified by flash column chromatography (ethyl acetate/hexanes: 1/20). Colorless liquid (87% yield). ^1^H NMR (300 MHz, CDCl_3_) δ 7.63–7.50 (m, 2H), 7.50–7.33 (m, 3H), 5.36 (s, 1H), 4.86 (dt, *J* = 8.4, 4.9 Hz, 1H), 1.99–1.16 (m, 11H). ^13^C NMR (75 MHz, CDCl_3_) δ 167.7, 136.1, 74.9, 59.5, 47.4, 31.2, 31.0, 25.3, 23.4, 23.3.

### 3.3. General Procedure for the Synthesis of **1bg**


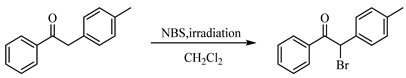


The mixture of 2-phenyl-1-p-tolyl-ethanone (10 mmol) and NBS (13 mmol) in CH_2_Cl_2_ and put under a 15 w ultraviolet lamp. After stirring for 3.5 h, it was quenched with saturated brine. The organic layer was separated, and the aqueous phase was extracted twice with CH_2_Cl_2_. The organic layer was concentrated under reduced pressure, and the resultant crude mixture was purified by silica gel column chromatography (n-hexane/EtOAc = 30/1) to give the desired 2-bromo-1-(4-methylphenyl)-2-phenyl-acetone (**1bg**).

#### 2-bromo-2-phenyl-1-(p-tolyl)ethenone (1bg) [23]

The compound was purified by flash column chromatography (ethyl acetate/hexanes: 1/20). White solid (70% yield). ^1^H NMR (300 MHz, DMSO-*d_6_*) δ 8.03–7.92 (m, 2H), 7.61–7.49 (m, 2H), 7.43–7.26 (m, 5H), 7.16 (d, *J* = 2.7 Hz, 1H), 2.34 (d, *J* = 2.4 Hz, 3H). ^13^C NMR (75 MHz, DMSO-*d_6_*) δ 191.4, 145.0, 137.1, 131.6, 129.9, 129.7, 129.3, 52.6, 21.6.

### 3.4. General Procedure for the Synthesis of (**2a**–**2bh**)


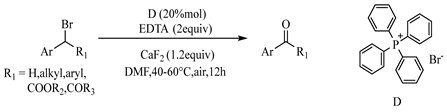


An oven-dried glass vial was charged with a magnetic stir bar, catalyst-D (20 mol%, 0.04 mmol), CaF_2_ (1.2 equiv, 0.24 mmol) and EDTA (2 equiv, 0.4 mmol). Moreover, 4 mL DMF was added to the mixture and stirred 10 min in an aerobic atmosphere, and then benzyl halide 1 (1.0 equiv, 0.2 mmol) was added. The vial was maintained for 12 h at 40 °C in a preheated oil bath. The organic layer was separated, and the aqueous phase was extracted twice with EtOAc; then, the collected organic phases were evaporated under pressure. The analytically pure product was obtained using petroleum ether and ethyl acetate as eluent by column chromatography on silica gel.

#### 3.4.1. Benzaldehyde (**2a**) [18]

The compound was purified by flash column chromatography (ethyl acetate/hexanes: 1/20). Colorless liquid (82% yield, 70% for 2a’). ^1^H NMR (300 MHz, CDCl_3_) δ 10.01 (s, 1H), 7.88 (d, *J* = 6.9 Hz, 2H), 7.62 (d, *J* = 7.3 Hz, 1H), 7.52 (t, *J* = 7.4 Hz, 2H). ^13^C NMR (75 MHz, CDCl_3_) δ 192.4, 134.5, 129.7, 129.0.

#### 3.4.2. 4-Cyanobenzaldehyde (**2b**) [18]

The compound was purified by flash column chromatography (ethyl acetate/hexanes: 1/10). White solid (79% yield). ^1^H NMR (300 MHz, CDCl_3_) δ 10.10 (d, *J* = 1.7 Hz, 1H), 8.10–7.90 (m, 2H), 7.90–7.64 (m, 2H).^13^C NMR (75 MHz, CDCl_3_) δ 190.6, 138.7, 132.9, 129.9, 117.7, 117.6, 77.5, 77.0, 76.6.

#### 3.4.3. 3-Cyanobenzaldehyde (**2c**) [24]

The compound was purified by flash column chromatography (ethyl acetate/hexanes: 1/10). White solid (77% yield). ^1^H NMR (300 MHz, CDCl_3_) δ 10.07 (s, 1H), 8.23–8.09 (m, 2H), 7.98–7.89 (m, 1H), 7.72 (t, *J* = 7.8 Hz, 1H). ^13^C NMR (75 MHz, CDCl_3_) δ 189.8, 137.2, 136.9, 133.2, 133.2, 130.0, 117.5, 113.9.

#### 3.4.4. 2-Cyanobenzaldehyde (**2d**) [25]

The compound was purified by flash column chromatography (ethyl acetate/hexanes: 1/10). White solid (65% yield). ^1^H NMR (300 MHz, CDCl_3_) δ 10.15 (s, 1H), 8.77–8.71 (m, 1H), 8.52 (ddd, *J* = 8.2, 2.3, 1.1 Hz, 1H), 8.26 (dt, *J* = 7.6, 1.3 Hz, 1H), 7.79 (t, *J* = 7.9 Hz, 1H). ^13^C NMR (75 MHz, CDCl_3_) δ 189.7, 137.4, 134.6, 130.4, 128.6, 124.5, 29.7.

#### 3.4.5. 4-Nitrobenzaldehyde (**2e**) [18]

The compound was purified by flash column chromatography (ethyl acetate/hexanes: 1/10). Yellow solid (90% yield). ^1^H NMR (300 MHz, CDCl_3_) δ 10.15 (s, 1H), 8.90–8.64 (m, 1H), 8.52 (ddd, *J* = 8.2, 2.3, 1.1 Hz, 1H), 8.26 (dt, *J* = 7.6, 1.3 Hz, 1H), 7.79 (t, *J* = 7.9 Hz, 1H). ^13^C NMR (75 MHz, CDCl_3_) δ 189.7, 137.4, 134.6, 130.4, 128.6, 124.5.

#### 3.4.6. 4-Chlorobenzaldehyde (**2f**) [18]

The compound was purified by flash column chromatography (ethyl acetate/hexanes: 1/10). Yellow solid (87% yield). ^1^H NMR (300 MHz, CDCl_3_) δ 10.00 (s, 1H), 7.84 (d, *J* = 8.2 Hz, 2H), 7.53 (d, *J* = 9.3 Hz, 2H). ^13^C NMR (75 MHz, CDCl_3_) δ 190.8, 140.9, 134.7, 130.9, 129.5.

#### 3.4.7. 4-Bromobenzaldehyde (**2g**) [26]

The compound was purified by flash column chromatography (ethyl acetate/hexanes: 1/10). White solid (82% yield). ^1^H NMR (300 MHz, CDCl_3_) δ 10.00 (s, 1H), 7.82–7.66 (m, 4H). ^13^C NMR (75 MHz, CDCl_3_) δ 191.0, 132.5, 131.0, 129.8.

#### 3.4.8. 4-Fluorobenzaldehyde (**2h**) [27]

The compound was purified by flash column chromatography (ethyl acetate/hexanes: 1/10). Colorless liquid (79% yield). ^1^H NMR (300 MHz, CDCl_3_) δ 9.98 (s, 1H), 7.92 (dd, *J* = 7.7, 6.5 Hz, 2H), 7.22 (t, *J* = 8.5 Hz, 2H). ^13^C NMR (75 MHz, CDCl_3_) δ 190.5, 168.2, 164.8, 132.3, 132.2, 116.5, 116.2.

#### 3.4.9. 4-Methylbenzaldehyde (**2i**) [18]

The compound was purified by flash column chromatography (ethyl acetate/hexanes: 1/10). Colorless liquid (95% yield, 72% for 2a′). ^1^H NMR (300 MHz, CDCl_3_) δ 9.91 (s, 1H), 7.73 (d, *J* = 8.0 Hz, 2H), 7.27 (d, *J* = 7.9 Hz, 2H), 2.38 (s, 3H). ^13^C NMR (75 MHz, CDCl_3_) δ 191.9, 145.5, 134.2, 129.6, 21.8.

#### 3.4.10. 2-Methoxybenzaldehyde (**2j**) [28]

The compound was purified by flash column chromatography (ethyl acetate/hexanes: 1/10). Yellow solid (75% yield). ^1^H NMR (300 MHz, CDCl_3_) δ 10.49 (s, 1H), 7.84 (dd, *J* = 7.7, 1.7 Hz, 1H), 7.57 (td, *J* = 8.5, 7.9, 1.8 Hz, 1H), 7.11–6.91 (m, 2H), 3.94 (s, 3H). ^13^C NMR (75 MHz, CDCl_3_) δ 189.8, 161.8, 136.0, 128.5, 124.8, 120.7, 111.6, 55.6.

#### 3.4.11. 2-Naphthaldehyde (**2k**) [18]

The compound was purified by flash column chromatography (ethyl acetate/hexanes: 1/20). Yellow solid (92% yield). ^1^H NMR (300 MHz, CDCl_3_) δ 10.19 (s, 1H), 8.37 (s, 1H), 8.07–7.90 (m, 4H), 7.73–7.55 (m, 2H). ^13^C NMR (75 MHz, CDCl_3_) δ 192.22, 136.46, 134.51, 134.1, 132.7, 129.5, 129.1, 128.0, 127.0, 122.8.

#### 3.4.12. Acetophenone (**2aa**) [18]

The compound was purified by flash column chromatography (ethyl acetate/hexanes: 1/10). Colorless liquid (88% yield). ^1^H NMR (300 MHz, CDCl_3_) δ 7.93 (d, *J* = 7.4 Hz, 2H), 7.48 (dt, *J* = 31.5, 7.3 Hz, 3H), 2.57 (s, 3H). ^13^C NMR (75 MHz, CDCl_3_) δ 198.0, 198.0, 137.0, 133.0, 128.5, 128.3, 26.6.

#### 3.4.13. 4-Methylacetophenone (**2ab**) [18]

The compound was purified by flash column chromatography (ethyl acetate/hexanes: 1/10). Colorless liquid (89% yield). ^1^H NMR (300 MHz, CDCl_3_) δ 7.85 (d, *J* = 8.2 Hz, 2H), 7.25 (d, *J* = 8.0 Hz, 2H), 2.57 (s, 3H), 2.40 (s, 3H). ^13^C NMR (75 MHz, CDCl_3_) δ 197.8, 143.8, 134.7, 129.2, 128.4, 26.5, 21.6.

#### 3.4.14. 4-Fluoroacetophenone (**2ac**) [29]

The compound was purified by flash column chromatography (ethyl acetate/hexanes: 1/10). Colorless liquid (86% yield). ^1^H NMR (300 MHz, CDCl_3_) δ 7.95 (dd, *J* = 8.4, 5.9 Hz, 2H), 7.09 (t, *J* = 8.7 Hz, 2H), 2.55 (s, 3H). ^13^C NMR (75 MHz, CDCl_3_) δ 196.4, 167.4, 164.0, 133.6, 131.0, 130.8, 115.7, 115.4, 100.0, 26.5.

#### 3.4.15. 4-Bromoacetophenone (**2ad**) [30]

The compound was purified by flash column chromatography (ethyl acetate/hexanes: 1/10). White solid (84% yield). ^1^H NMR (300 MHz, CDCl_3_) δ 7.82 (d, *J* = 8.7 Hz, 2H), 7.60 (d, *J* = 8.7 Hz, 2H), 2.59 (s, 3H). ^13^C NMR (75 MHz, CDCl_3_) δ 197.0, 135.8, 131.9, 129.8, 128.3, 26.6.

#### 3.4.16. 4-Methoxyacetophenone (**2ae**) [31]

The compound was purified by flash column chromatography (ethyl acetate/hexanes: 1/10). White solid (92% yield). ^1^H NMR (300 MHz, CDCl_3_) δ 7.95 (d, *J* = 9.0 Hz, 2H), 6.94 (d, *J* = 8.9 Hz, 2H), 3.88 (s, 3H), 2.57 (s, 3H). ^13^C NMR (75 MHz, CDCl_3_) δ 196.8, 163.5, 130.6, 113.7, 100.0, 55.5, 26.4.

#### 3.4.17. 2-bromo-1-phenylethan-1-one (**2af**) [18]

The compound was purified by flash column chromatography (ethyl acetate/hexanes: 1/20). Yellow solid (42% yield). ^1^H NMR (300 MHz, CDCl_3_) δ 8.06–7.96 (m, 2H), 7.69–7.58 (m, 1H), 7.52 (t, *J* = 7.5 Hz, 2H), 4.48 (s, 2H). ^13^C NMR (75 MHz, CDCl_3_) δ 191.3, 134.0, 128.9, 30.9.

#### 3.4.18. Benzophenone (**2ag**) [18]

The compound was purified by flash column chromatography (ethyl acetate/hexanes: 1/10). White solid (77% yield). ^1^H NMR (300 MHz, CDCl_3_) δ 7.85–7.78 (m, 4H), 7.61 (t, *J* = 7.4 Hz, 2H), 7.50 (t, *J* = 7.6 Hz, 4H). ^13^C NMR (75 MHz, CDCl_3_) δ 196.7, 137.6, 132.4, 130.0, 128.3.

#### 3.4.19. (4-fluorophenyl)phenylmethanone (**2ah**) [18]

The compound was purified by flash column chromatography (ethyl acetate/hexanes: 1/10). White solid (63% yield). ^1^H NMR (300 MHz, CDCl_3_) δ 7.87 (dd, *J* = 8.6, 5.6 Hz, 2H), 7.79 (d, *J* = 7.2 Hz, 2H), 7.62 (t, *J* = 7.4 Hz, 1H), 7.51 (t, *J* = 7.7 Hz, 2H), 7.18 (t, *J* = 8.6 Hz, 2H). ^13^C NMR (75 MHz, CDCl_3_) δ 195.3, 167.1, 163.7, 137.5, 133.8, 133.8, 132.7, 132.6, 132.5, 129.9, 128.4, 115.6, 115.3, 77.0.

#### 3.4.20. (4-chlorophenyl)(phenyl)methanone (**2ai**) [32]

The compound was purified by flash column chromatography (ethyl acetate/hexanes: 1/10). White solid (65% yield). ^1^H NMR (300 MHz, CDCl_3_) δ 7.78 (dd, *J* = 7.8, 5.6 Hz, 4H), 7.62 (t, *J* = 7.4 Hz, 1H), 7.50 (dd, *J* = 12.3, 8.2 Hz, 4H). ^13^C NMR (75 MHz, CDCl_3_) δ 190.5, 168.2, 164.8, 133.0, 132.3, 132.1, 116.5, 116.2.

#### 3.4.21. Bis(4-chlorophenyl)methanone (**2aj**) [33]

The compound was purified by flash column chromatography (ethyl acetate/hexanes: 1/10). White solid (61% yield). _1_H NMR (300 MHz, CDCl_3_) δ 7.75 (d, *J* = 8.4 Hz, 4H), 7.49 (d, *J* = 8.5 Hz, 4H). ^13^C NMR (75 MHz, CDCl_3_) δ 194.2, 139.2, 135.5, 131.3, 128.8.

#### 3.4.22. Methyl benzoylformate (**2ba**) [16]

The compound was purified by flash column chromatography (ethyl acetate/hexanes: 1/20). Colorless liquid (82% yield). ^1^H NMR (300 MHz, CDCl_3_) δ 8.13–7.94 (m, 2H), 7.77–7.62 (m, 1H), 7.59–7.47 (m, 2H), 4.01 (s, 3H). ^13^C NMR (75 MHz, CDCl_3_) δ 186.0, 164.0, 135.0, 132.4, 130.1, 128.9, 127.9, 100.0.

#### 3.4.23. Ethylbenzoylformate (**2bb**) [16]

The compound was purified by flash column chromatography (ethyl acetate/hexanes: 1/20). Colorless liquid (79% yield). ^1^H NMR (300 MHz, CDCl^3^) δ 8.03 (dd, *J* = 8.2, 1.0 Hz, 2H), 7.74–7.65 (m, 1H), 7.54 (t, *J* = 7.7 Hz, 2H), 4.48 (q, *J* = 7.1 Hz, 2H), 1.45 (t, *J* = 7.2 Hz, 3H). ^13^C NMR (75 MHz, CDCl_3_) δ 186.4, 163.8, 134.9, 132.5, 130.0, 128.9, 128.6, 128.5, 62.3.

#### 3.4.24. Isopropyl benzoylcarboxylate (**2bc**) [16]

The compound was purified by flash column chromatography (ethyl acetate/hexanes: 1/20). Colorless liquid (79% yield). ^1^H NMR (300 MHz, CDCl_3_) δ 8.01 (d, *J* = 8.4 Hz, 2H), 7.68 (t, *J* = 8.0 Hz, 1H), 7.53 (t, *J* = 7.9 Hz, 2H), 5.42–5.27 (m, 1H), 1.43 (d, *J* = 7.2 Hz, 6H). ^13^C NMR (75 MHz, CDCl_3_) δ 186.7, 163.6, 134.8, 132.5, 130.0, 128.9, 70.7, 21.7.

#### 3.4.25. Tert-butyl benzoylcarboxylate (**2bd**) [34]

The compound was purified by flash column chromatography (ethyl acetate/hexanes: 1/20). Colorless liquid (75% yield). ^1^H NMR (300 MHz, CDCl_3_) δ 8.05–7.95 (m, 2H), 7.72–7.63 (m, 1H), 7.59–7.49 (m, 2H), 1.66 (s, 9H). ^13^C NMR (75 MHz, CDCl_3_) δ 186.7, 163.6, 134.8, 132.5, 130.0, 128.9, 70.7, 21.7.

#### 3.4.26. Cyclohexyl benzoylcarboxylate (**2be**) [35]

The compound was purified by flash column chromatography (ethyl acetate/hexanes: 1/20). Colorless liquid (72% yield). ^1^H NMR (300 MHz, CDCl_3_) δ 8.10–7.95 (m, 2H), 7.75–7.60 (m, 1H), 7.53 (t, *J* = 7.7 Hz, 2H), 5.11 (tt, *J* = 8.9, 3.9 Hz, 1H), 2.13–1.92 (m, 2H), 1.81 (dt, *J* = 13.8, 4.9 Hz, 3H), 1.62 (td, *J* = 13.0, 6.7 Hz, 5H). ^13^C NMR (75 MHz, CDCl_3_) δ 186.8, 163.7, 134.8, 132.6, 129.9, 128.9, 127.9, 75.5, 31.4, 25.2, 23.6.

#### 3.4.27. 4-Fluorobenzil (**2bf**) [33]

The compound was purified by flash column chromatography (ethyl acetate/hexanes: 1/20). Yellow oil (68% yield). ^1^H NMR (300 MHz, CDCl_3_) δ 8.10–7.95 (m, 4H), 7.75–7.64 (m, 1H), 7.54 (t, *J* = 7.6 Hz, 2H), 7.26–7.15 (m, 2H). ^13^C NMR (75 MHz, CDCl_3_) δ 194.0, 192.7, 168.5, 165.0, 136.1, 135.0, 132.9, 131.5, 129.9, 129.0, 128.0, 116.5, 116.2, 115.6.

#### 3.4.28. (4-Methylphenyl)phenylethanedione (**2bg**) [33]

The compound was purified by flash column chromatography (ethyl acetate/hexanes: 1/20). Yellow oil (71% yield). ^1^H NMR (300 MHz, CDCl_3_) δ 7.93 (dd, *J* = 29.7, 7.8 Hz, 4H), 7.67 (t, *J* = 7.4 Hz, 1H), 7.52 (t, *J* = 7.7 Hz, 2H), 7.33 (d, *J* = 8.1 Hz, 2H), 2.45 (s, 3H). ^13^C NMR (75 MHz, CDCl_3_) δ 194.8, 194.3, 146.2, 134.8, 133.0, 129.9, 129.7, 129.0, 22.0.

#### 3.4.29. Phthalic anhydride (**2bh**) [36]

The compound was purified by flash column chromatography (ethyl acetate/hexanes: 1/20). White solid (50% yield). ^1^H NMR (300 MHz, DMSO-d_6_) δ 7.62 (d, *J* = 21.4 Hz, 4H). ^13^C NMR (75 MHz, DMSO-d_6_) δ 169.0, 133.3, 131.2, 128.8.

## 4. Conclusions

In this article, tetraphenylphosphonium bromide was described as an effective organocatalyst for the oxidation of halide ions at the *α*-position of aryl groups to carbonyl groups. A mechanistically sound and unique oxidation-catalyzed cycle was successfully designed using hydrolysis of Ph_4_PBr and H^+^/COO^-^ generated by EDTA and CaF_2_. Triphenylphosphine autoxidizes under aerobic conditions to develop a pentavalent phosphine oxidant, allowing the reaction to be realized under catalytic amounts of oxidant.

## Figures and Tables

**Table 1:** Optimization studies.

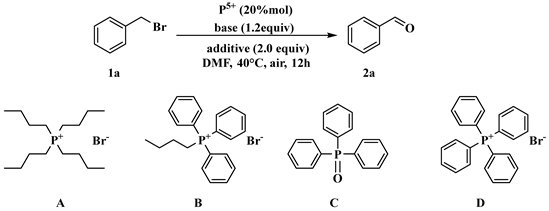					
Entry	P^5+^	Loading (P^5+^)	Additive	Base	Yield (%) ^a,b^
1	D	20 mol%	-	-	0
2	D	20 mol%	EDTA	-	<5
3	D	20 mol%	-	CaF_2_	0
4	D	20 mol%	EDTA	CaF_2_	82 ^c^
5	A	20 mol%	EDTA	CaF_2_	0
6	B	20 mol%	EDTA	CaF_2_	30
7	C	20 mol%	EDTA	CaF_2_	37
8	D	20 mol%	EDTA	TEA	56
9	D	20 mol%	EDTA	KF	42
10	D	20 mol%	EDTA	TMG	85 ^d^
11	D	20 mol%	18-Crown-6	CaF_2_	40 ^e^
12	D	20 mol%	HFIP	CaF_2_	35
13	D	20 mol%	EDTA	CaF_2_	30 ^f^
14	D	10 mol%	EDTA	CaF_2_	50 ^g^
15	D	15 mol%	EDTA	CaF_2_	72
16	D	25 mol%	EDTA	CaF_2_	83
17	—		EDTA	CaF_2_	0

^a^ Reaction conditions: 1a (1.0 eq., 0.2 mmol), P^5+^ (20 mol%, 0.04 mmol), base (1.2 eq), additive (2 eq.), solvent DMF (undistilled, 4 mL), 40 °C, air, 12 h. ^b^ Determination of the ^1^H NMR standard with 1,3,5-trimethoxybenzene as an internal yield. ^c^ Isolated yield. ^d^ Reaction time is 3 h, but there was a 0% yield when esters were the substrate. ^e^ 50% PhCOOH was isolated with 2a. ^f^ Reaction carried out at room temperature. ^g^ Other reaction conditions, the same as footnote **a**.

## Data Availability

The original contributions presented in the study are included in the article/Supplementary Materials; further inquiries can be directed to the corresponding authors.

## Supplementary Materials

The following supporting information can be downloaded at: https://www.mdpi.com/article/10.3390/molecules31203742/s1, spectral data and references are available; Figures S1–S70: ^1^H, ^13^C NMR.
