# Supplementary material for: Reaction Studies on the Conversion of Aryl Methyl Halides to Aryl Carbonyl Compounds Using Ph4PBr/EDTA/CaF2 as a Catalytic Oxidation System
Source: Molecules. 2026 Oct 18;31(20):3742. doi: 10.3390/molecules31203742 (PMC13707991; doi:10.3390/molecules31203742)
Supplement: Supplementary file 1 [file molecules-31-03742-s001.zip › molecules-4514331-supplementary.pdf]

Supporting Information

Reaction studies on the conversion of aryl methyl halides to aryl carbonyl compounds using EDTA/$Ph_4P^+Br^-$ as a catalytic oxidation system

Xianwei Xu[a], Jun You[a], Linpeng Song[a], Yanchao Yu[a], Wenju WU[a*]*

[a]Key Laboratory of Green Chemical Engineering and Technology of College of Heilongjiang Province, School of Materials Science and Chemical Engineering, Harbin University of Science and Technology, Harbin, 150080, P. R. China.

[*] Corresponding author. Email: youjunjun@126.com; wuwenju1017@163.com

Contents

1. Experimental NMR Data 5

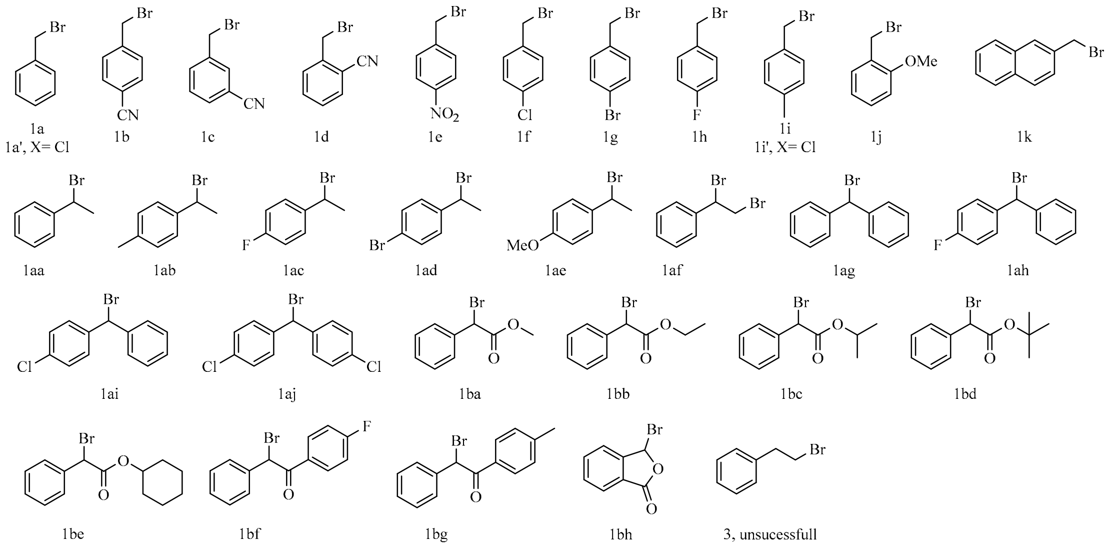
1a
1a', X= Cl
1b
1c
1d
1e
1f
1g
1h
1i
1i', X= Cl
1j
1k
1aa
1ab
1ac
1ad
1ae
1af
1ag
1ah
1ai
1aj
1ba
1bb
1bc
1bd
1be
1bf
1bg
1bh
3, unsucessfull

1. Experimental NMR Data

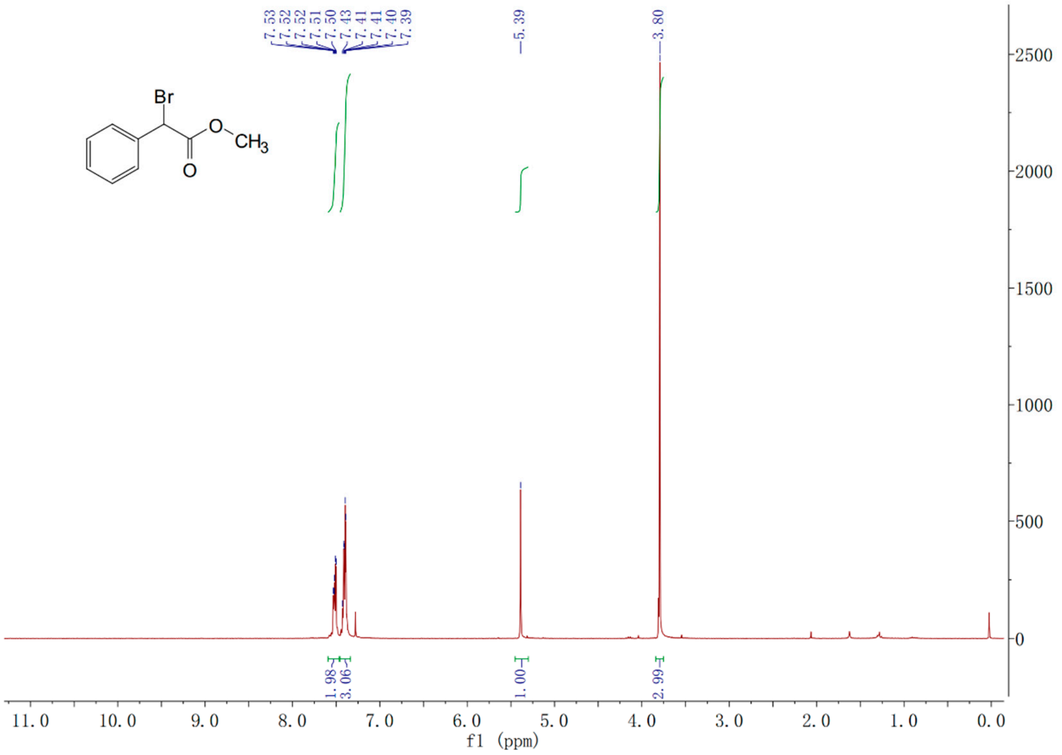

Figure S1. ^{1}H NMR spectrum of 1ba in $CDCl_3$ at room temperature

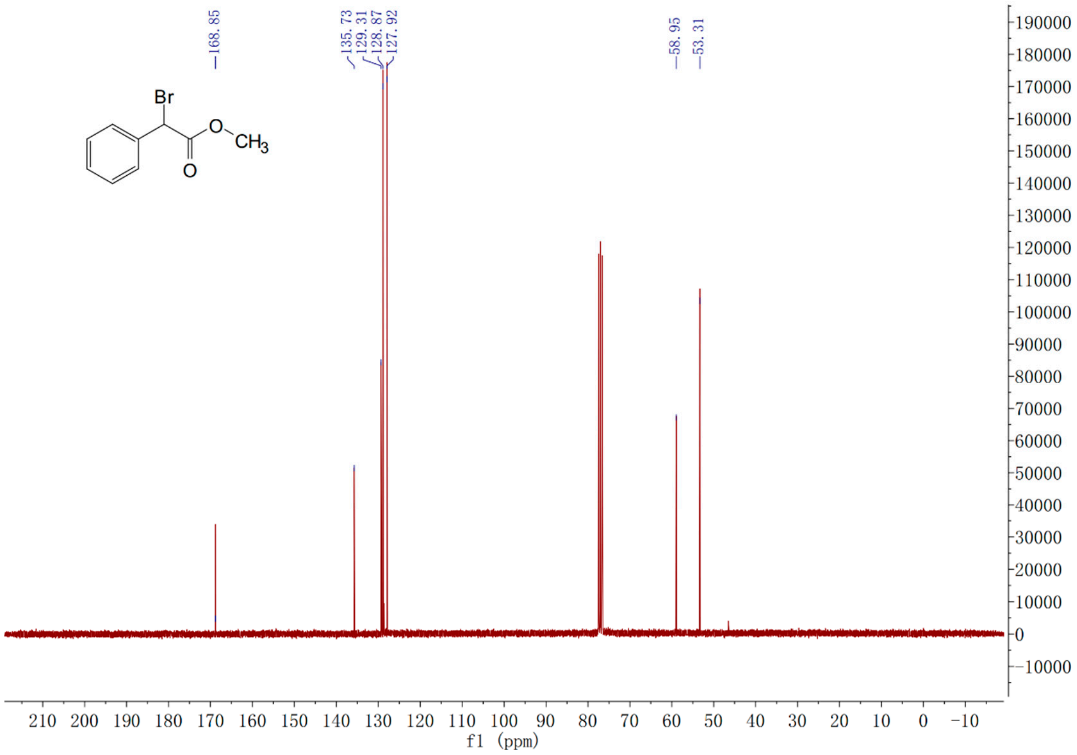

Figure S2. ^{13}C NMR spectrum of 1ba in $CDCl_3$ at room temperature

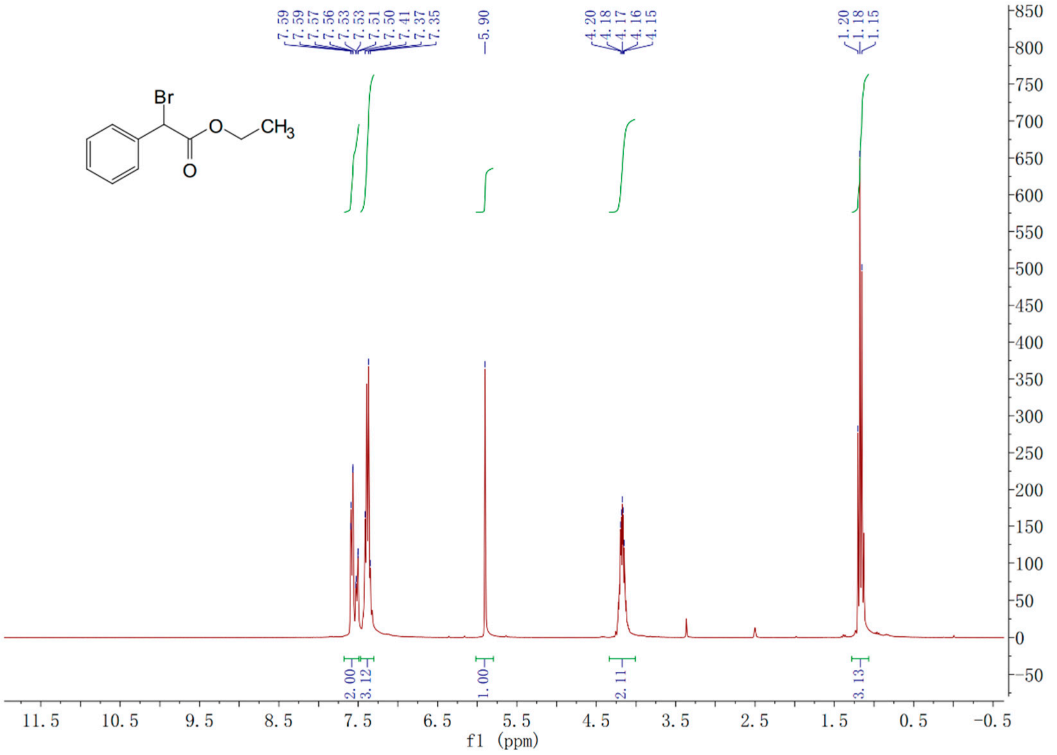

Figure S3. ^{1}H NMR spectrum of 1bb in $CDCl_3$ at room temperature

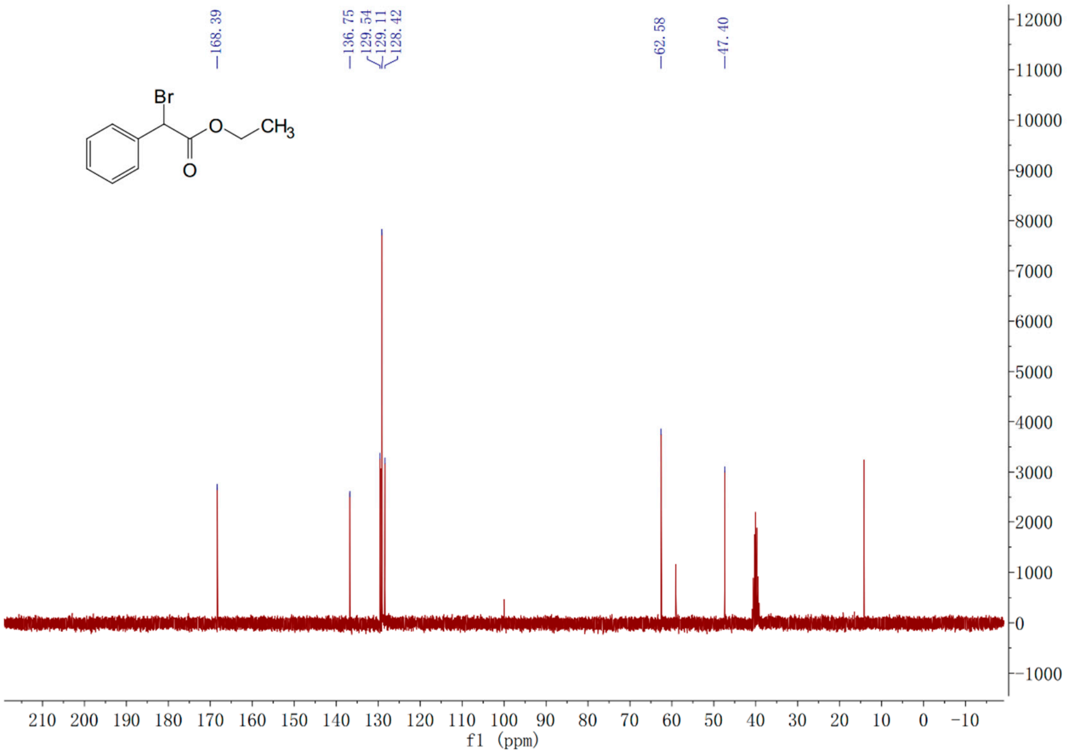

Figure S4. ^{13}C NMR spectrum of 1bb in $CDCl_3$ at room temperature

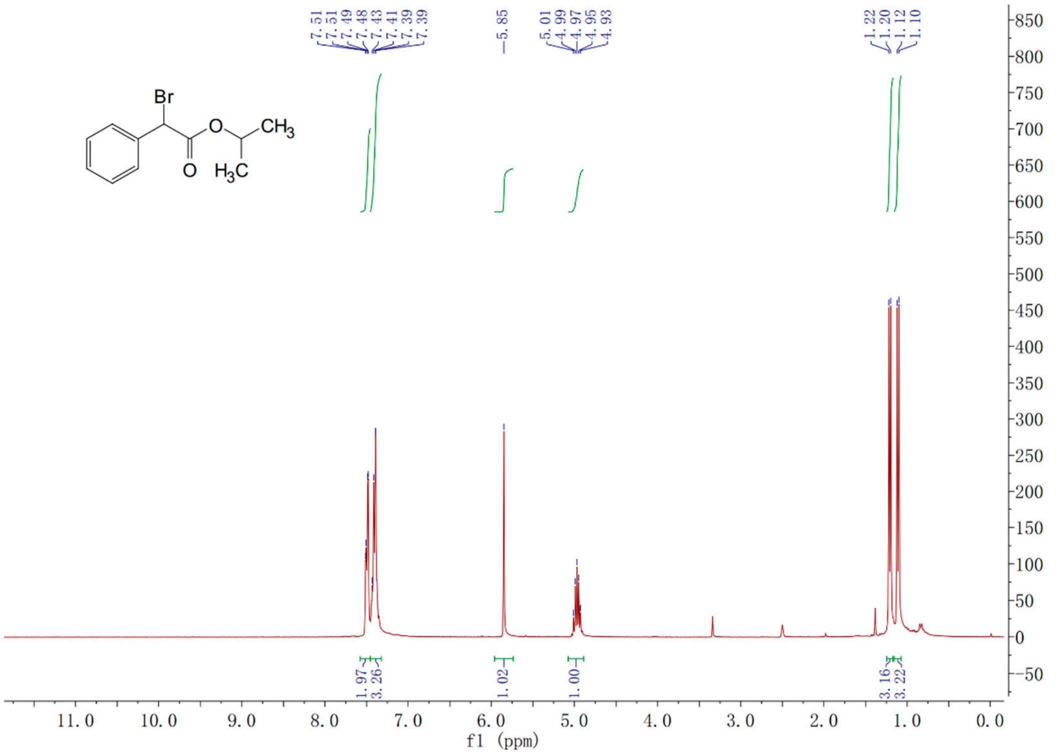

Figure S5. ^{1}H NMR spectrum of 1bc in DMSO at room temperature

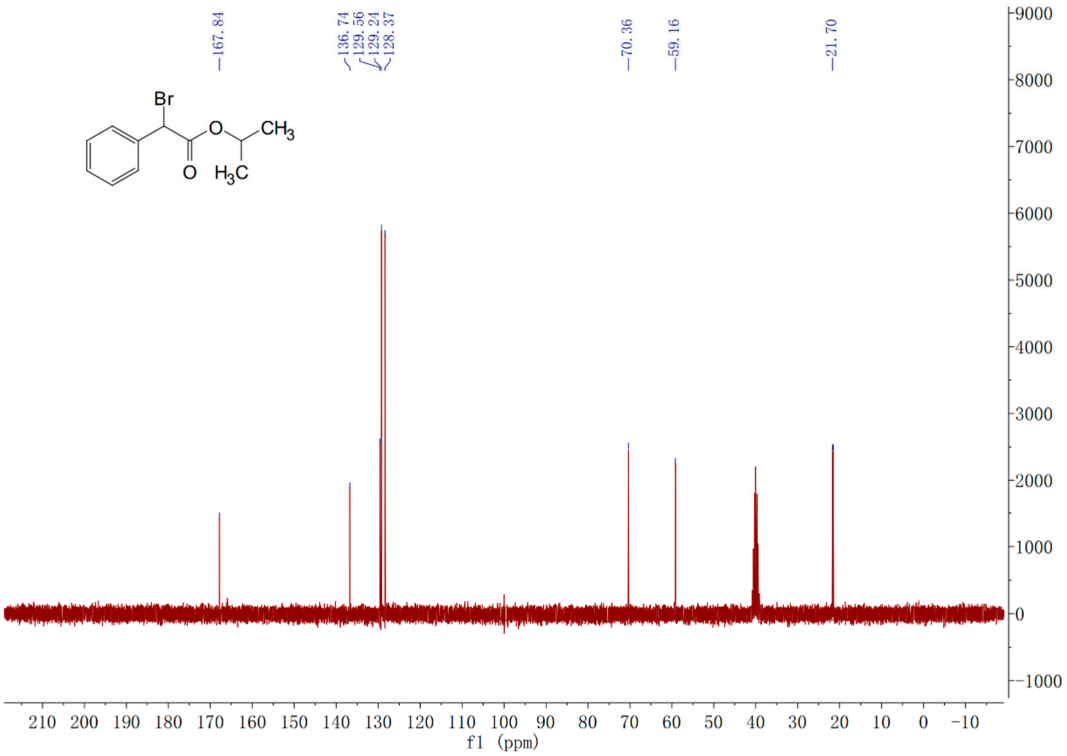

Figure S6. ^{13}C NMR spectrum of 1bc in DMSO at room temperature

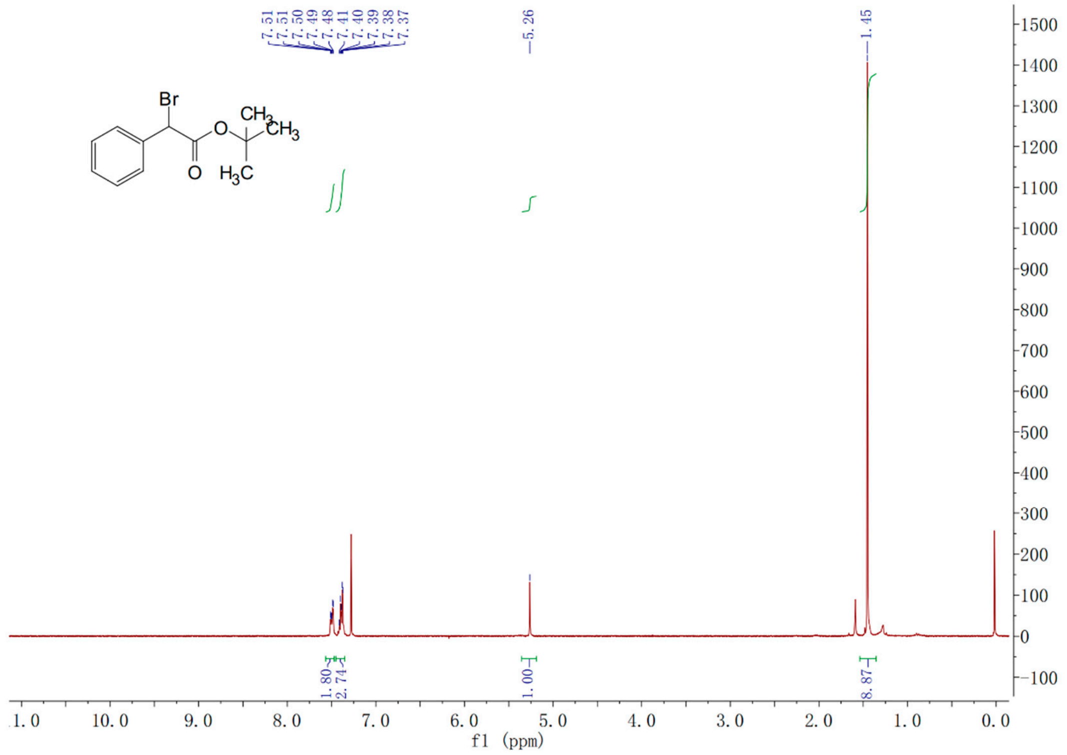

Figure S7. ^{1}H NMR spectrum of 1bd in $CDCl_3$ at room temperature

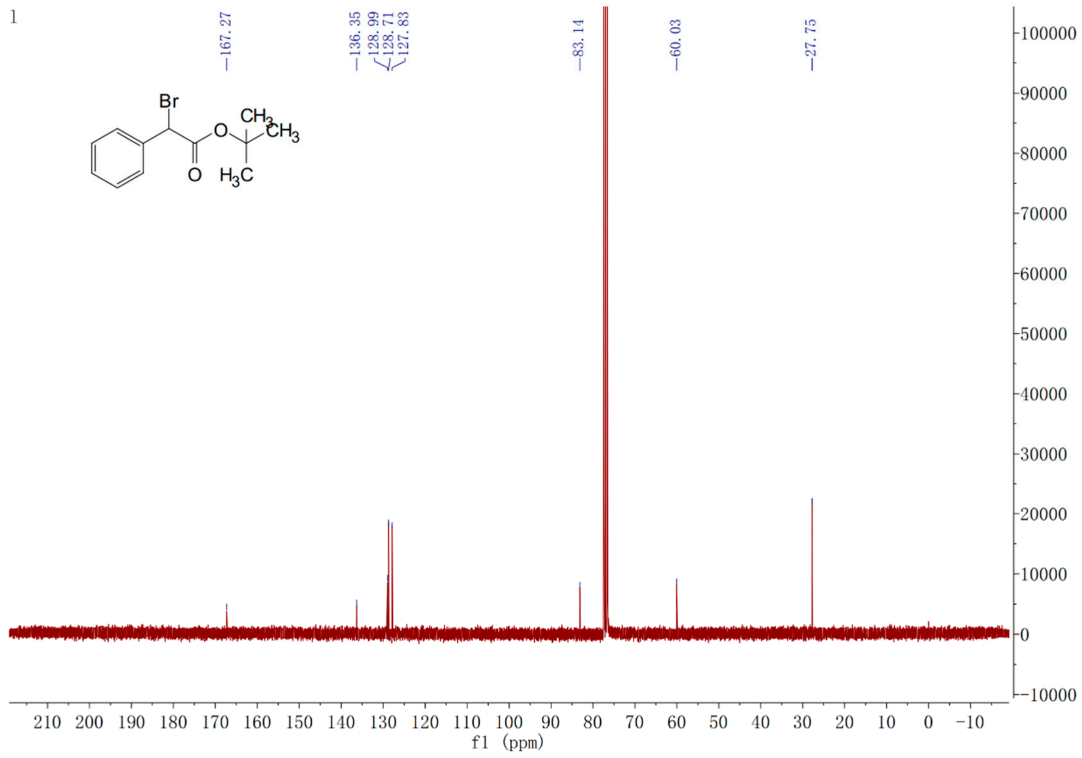

Figure S8. ^{13}C NMR spectrum of 1bd in $CDCl_3$ at room temperature

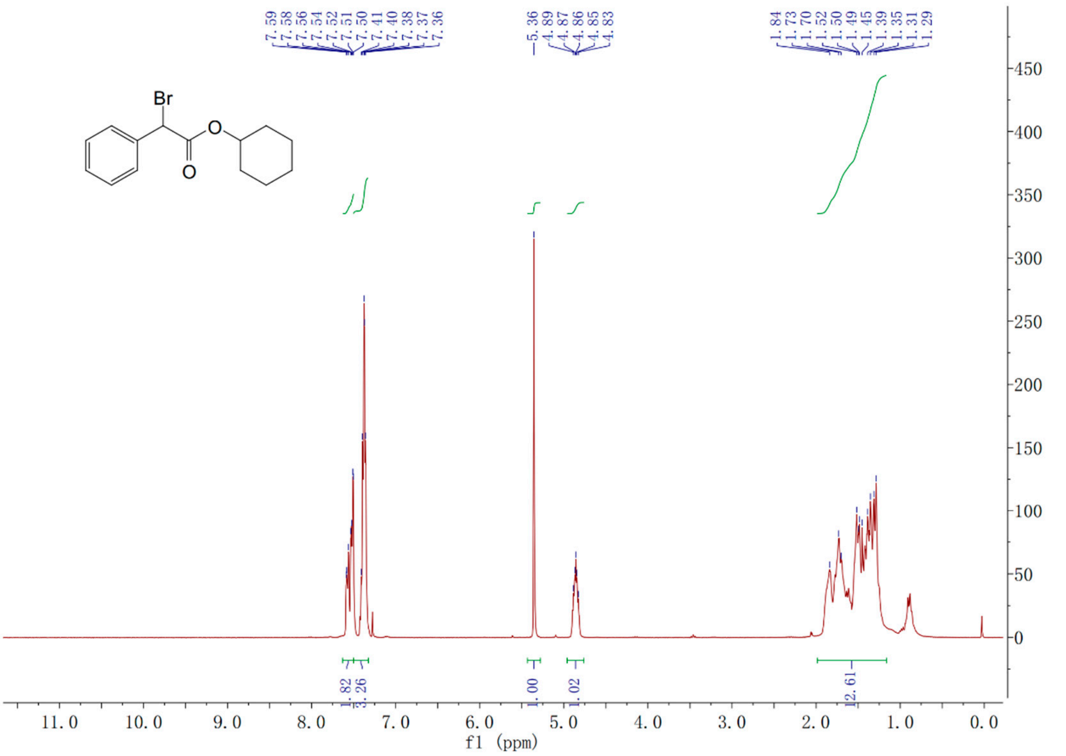

Figure S9. ^{1}H NMR spectrum of 1be in $CDCl_3$ at room temperature

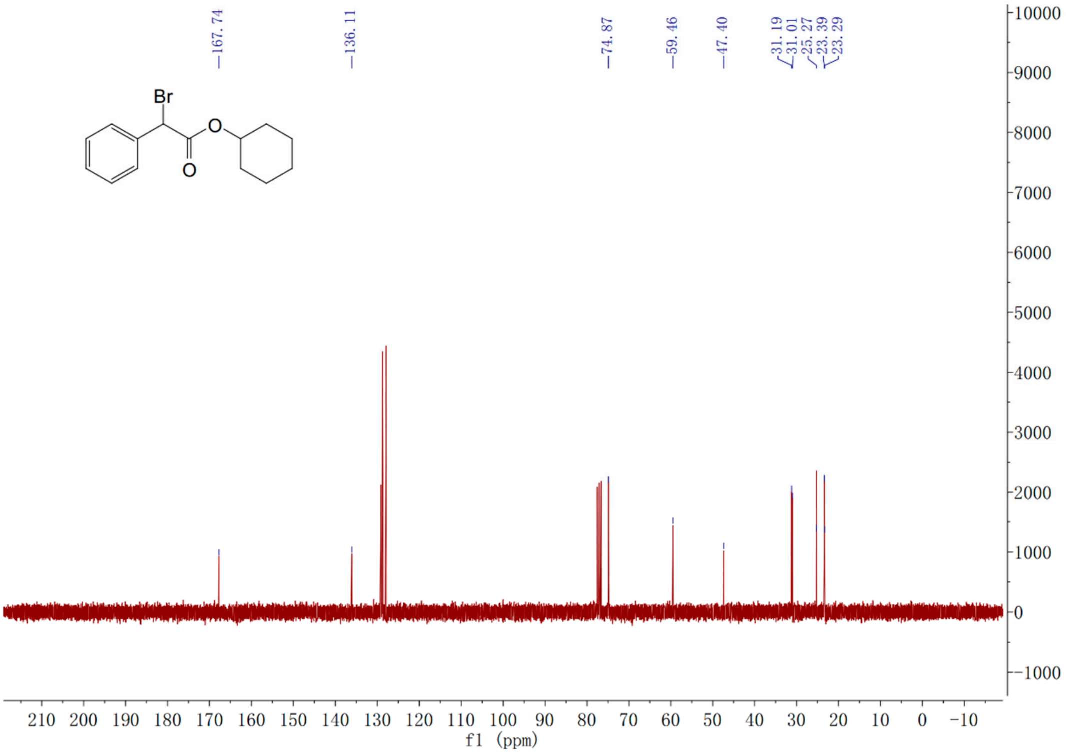

Figure S10. ^{13}C NMR spectrum of 1be in $CDCl_3$ at room temperature

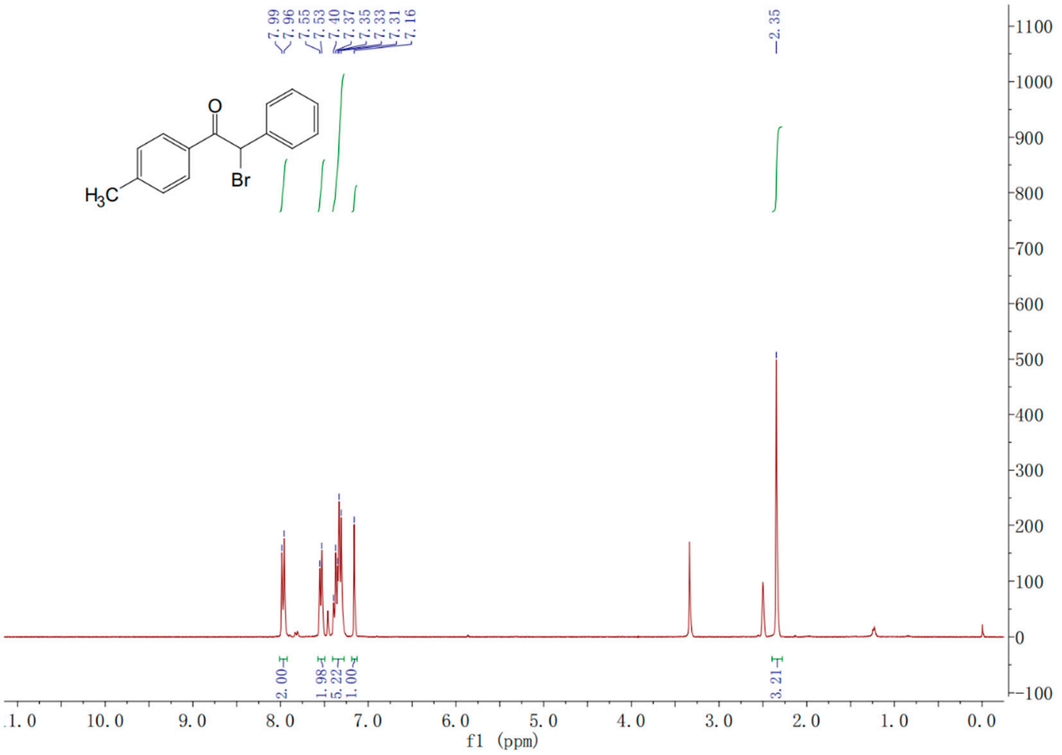

Figure S11. ^{1}H NMR spectrum of 1bg in DMSO at room temperature

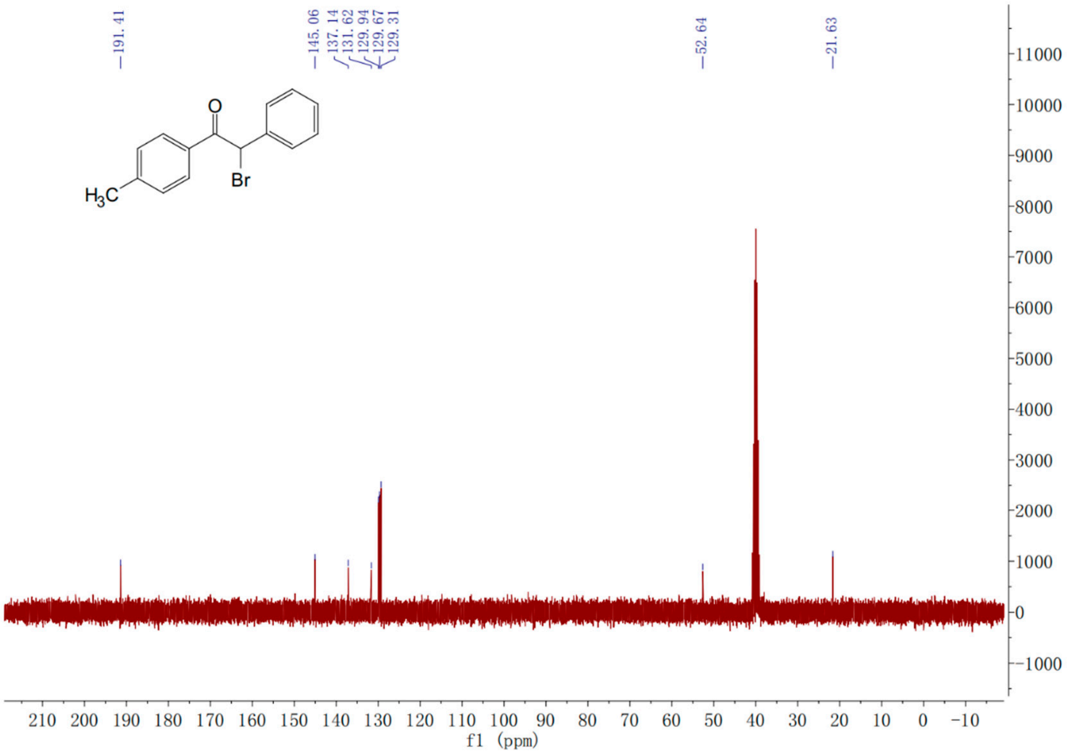

Figure S12. ^{13}C NMR spectrum of 1bg in DMSO at room temperature

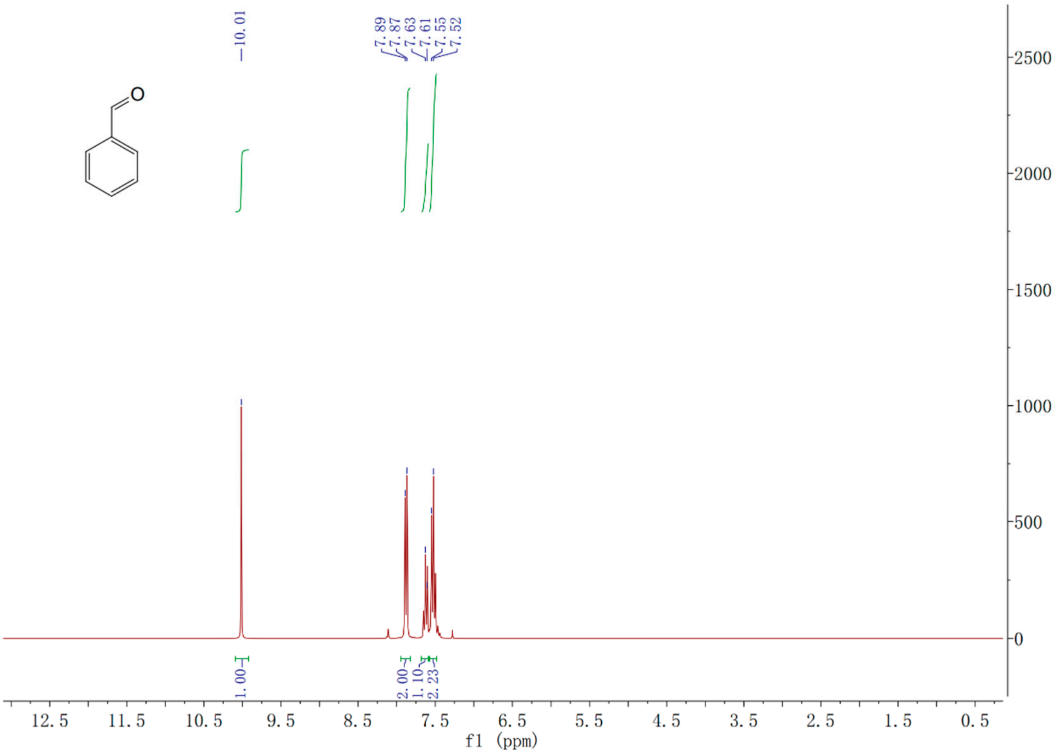

Figure S13. ^{1}H NMR spectrum of 2a in $CDCl_3$ at room temperature

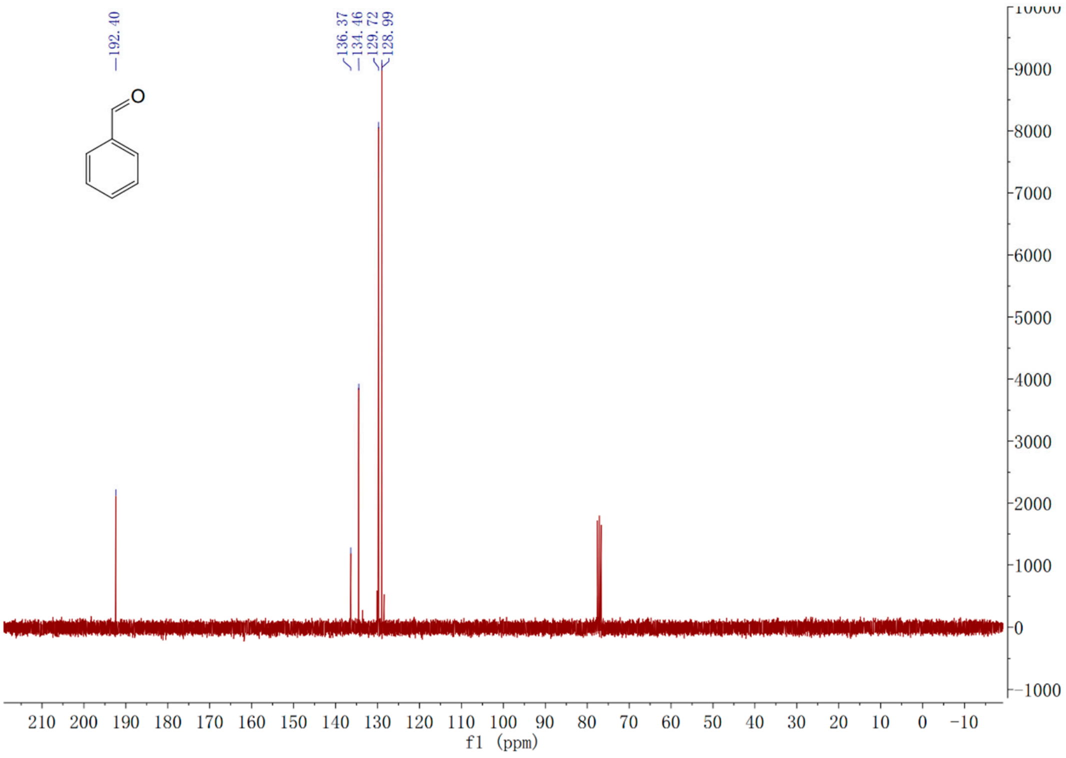

Figure S14. ^{13}C NMR spectrum of 2a in $CDCl_3$ at room temperature

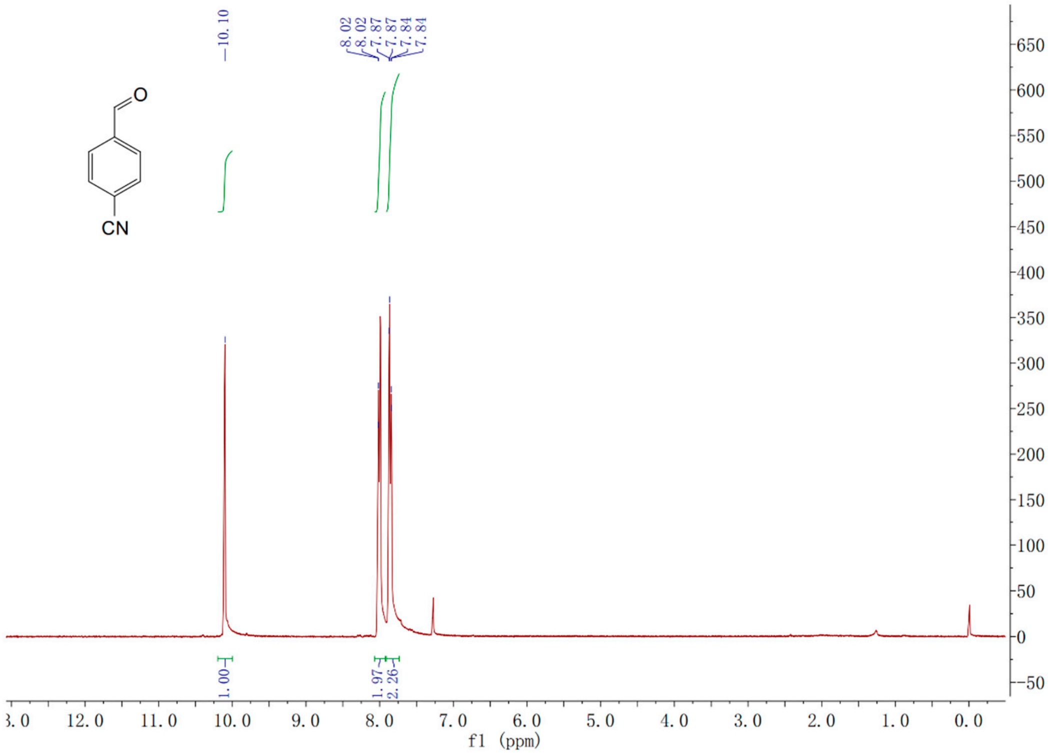

Figure S15. ^{1}H NMR spectrum of 2b in $CDCl_3$ at room temperature

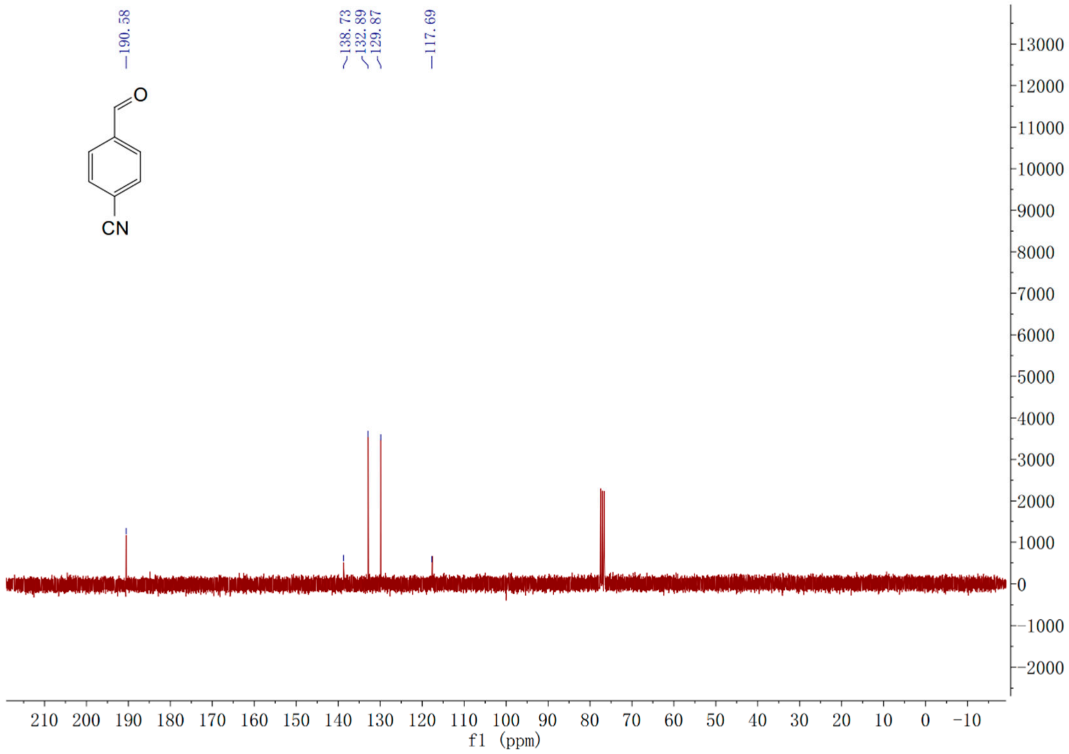

Figure S16. ^{13}C NMR spectrum of 2b in $CDCl_3$ at room temperature

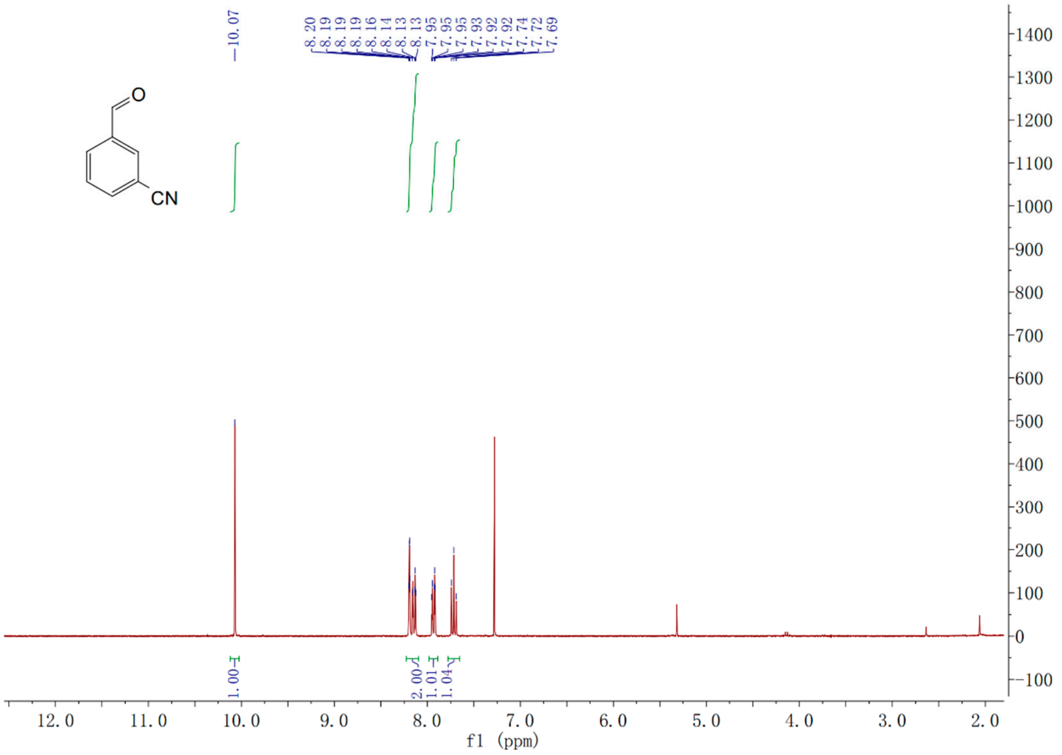

Figure S17. ^{1}H NMR spectrum of 2c in $CDCl_3$ at room temperature

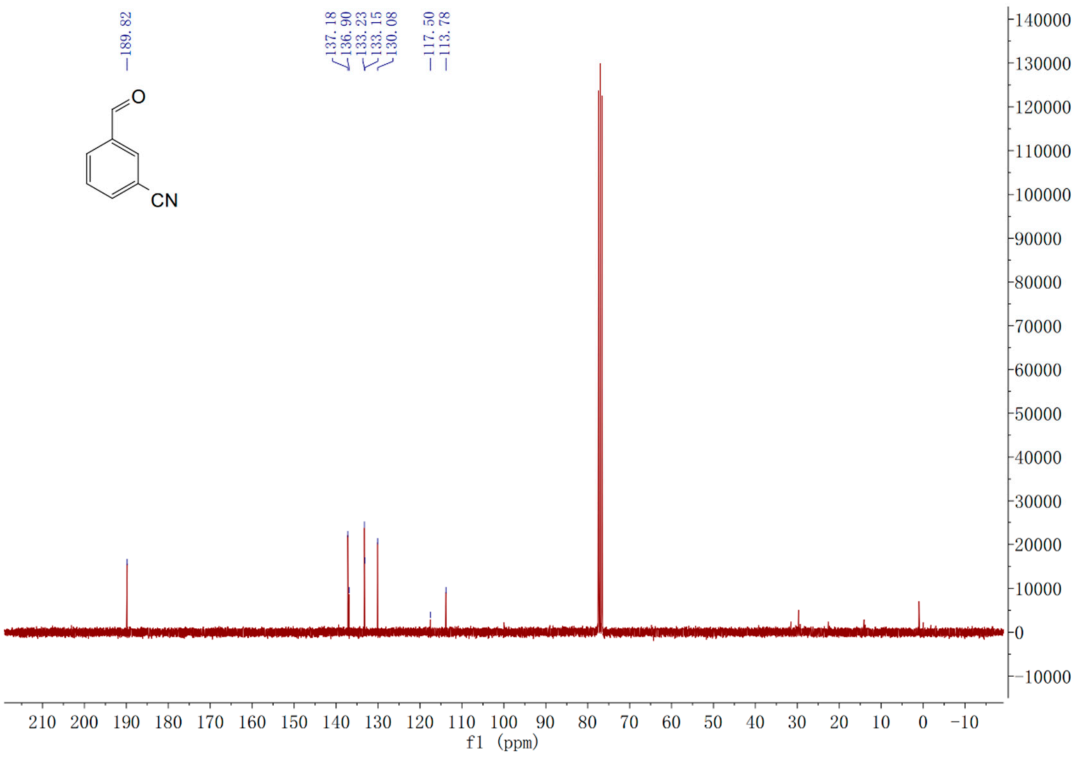

Figure S18. ^{13}C NMR spectrum of 2c in $CDCl_3$ at room temperature

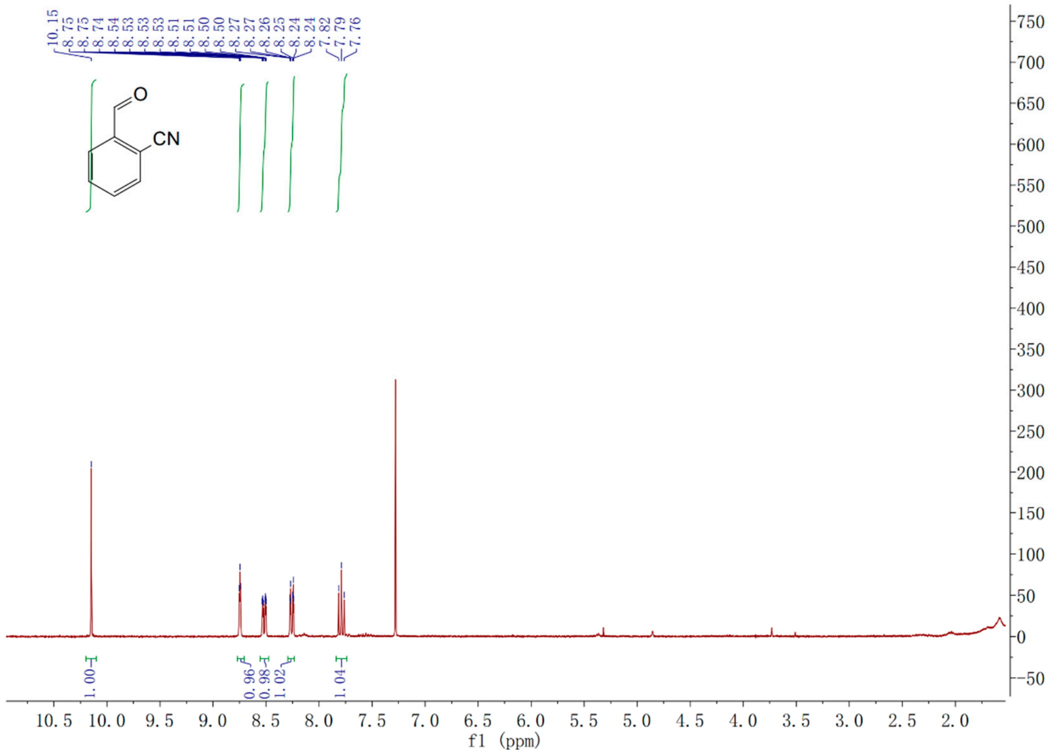

Figure S19. ^{1}H NMR spectrum of 2d in $CDCl_3$ at room temperature

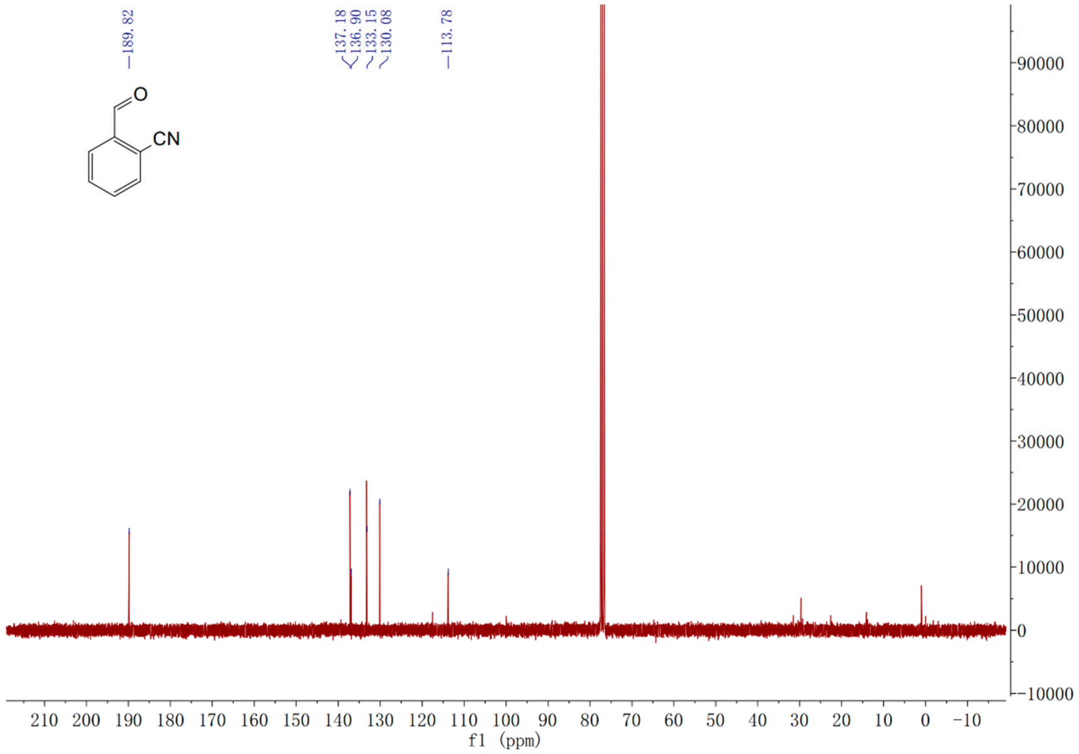

Figure S20. ^{13}C NMR spectrum of 2d in $CDCl_3$ at room temperature

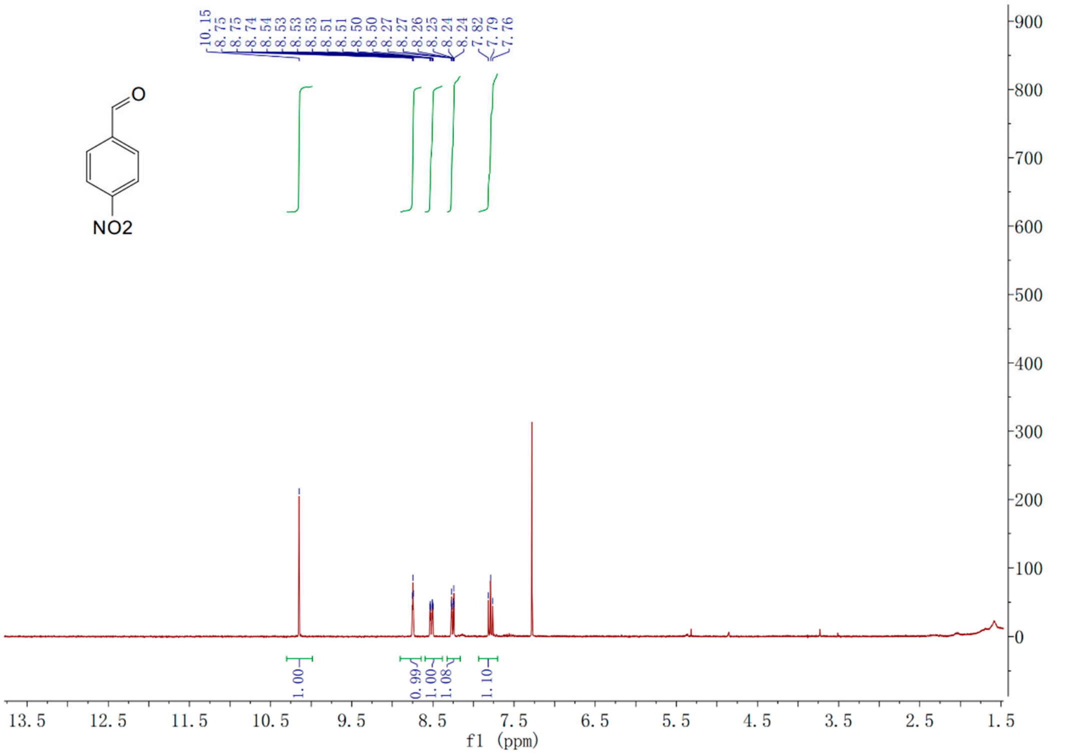

Figure S21. ^{1}H NMR spectrum of 2e in $CDCl_3$ at room temperature

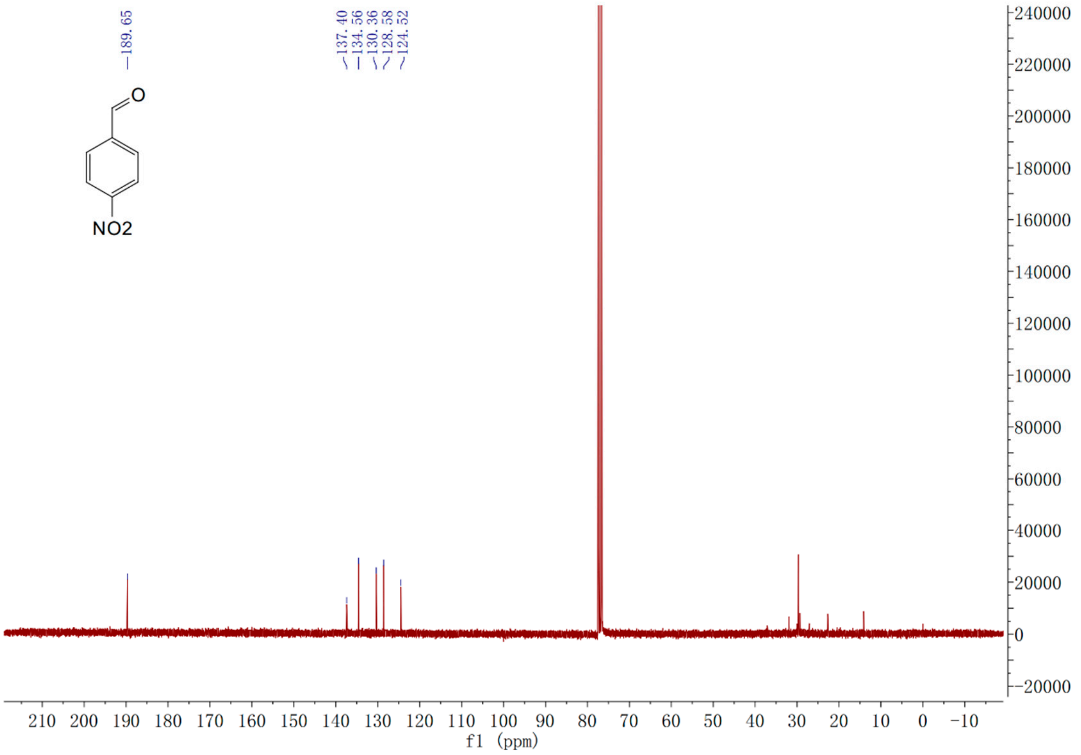

Figure S22. ^{13}C NMR spectrum of 2e in $CDCl_3$ at room temperature

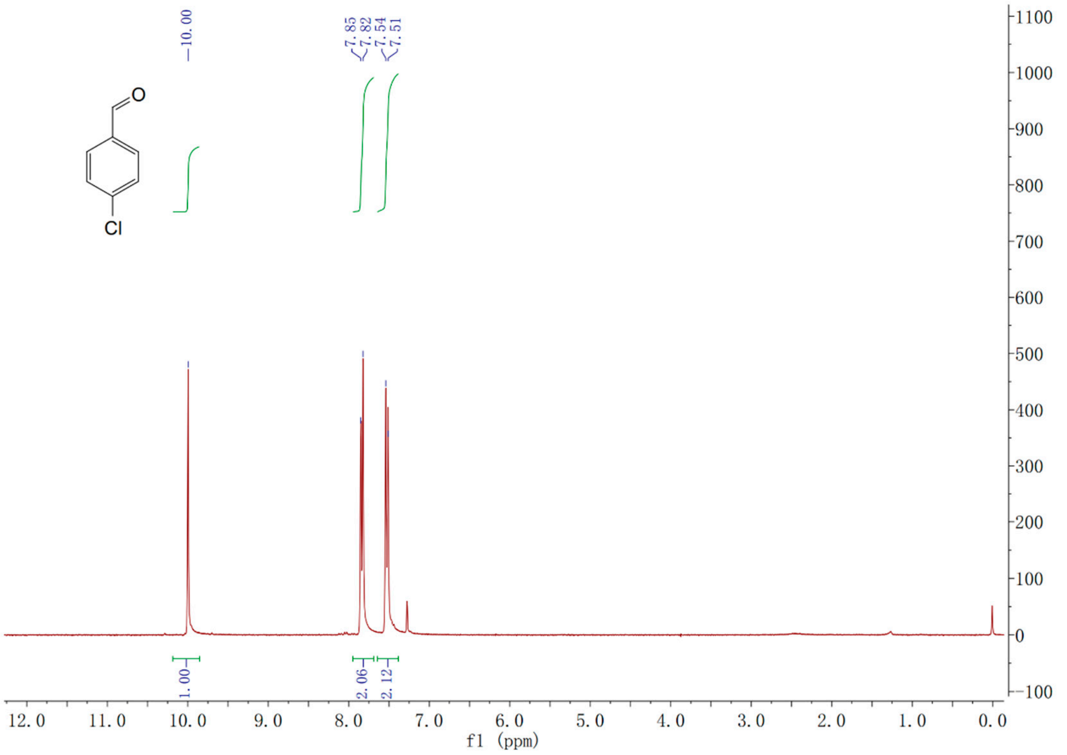

Figure S23. ^{1}H NMR spectrum of 2f in $CDCl_3$ at room temperature

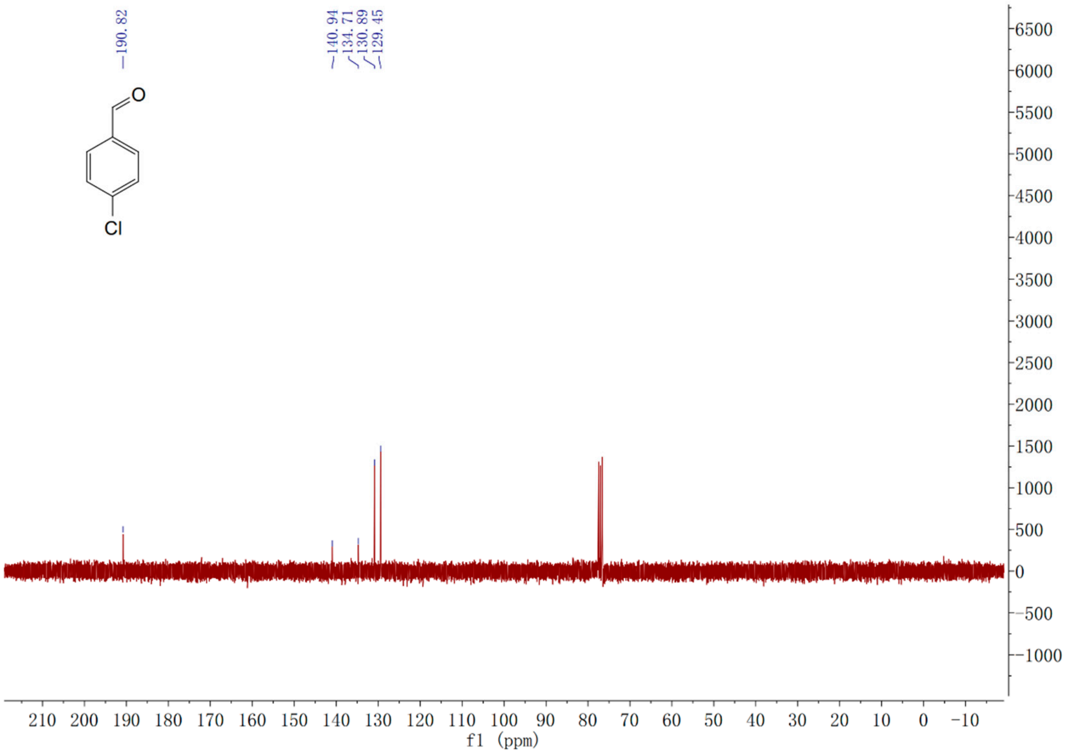

Figure S24. ^{13}C NMR spectrum of 2f in $CDCl_3$ at room temperature

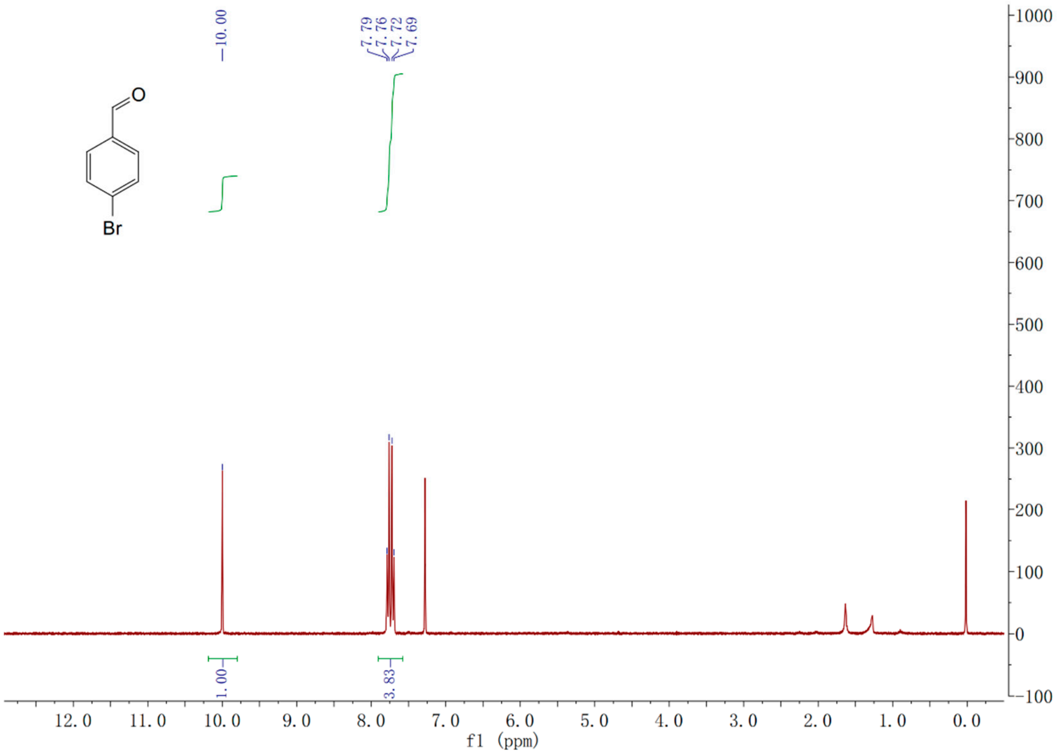

Figure S25. ^{1}H NMR spectrum of 2g in $CDCl_3$ at room temperature

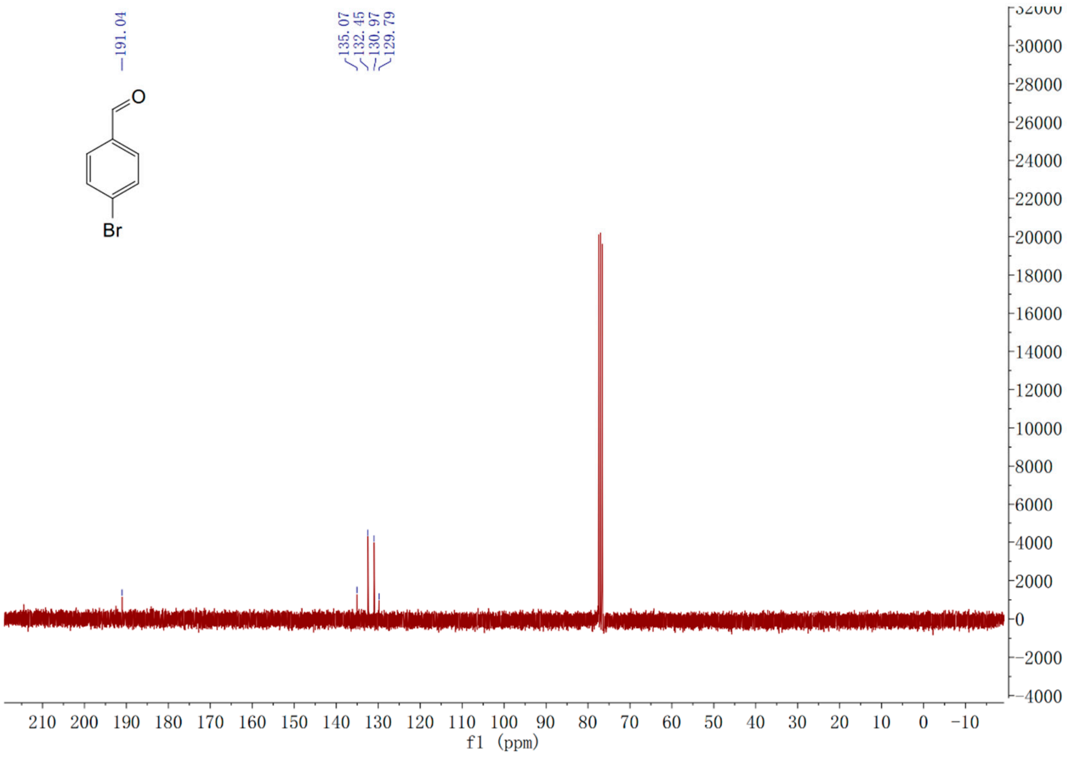

Figure S26. ^{13}C NMR spectrum of 2g in $CDCl_3$ at room temperature

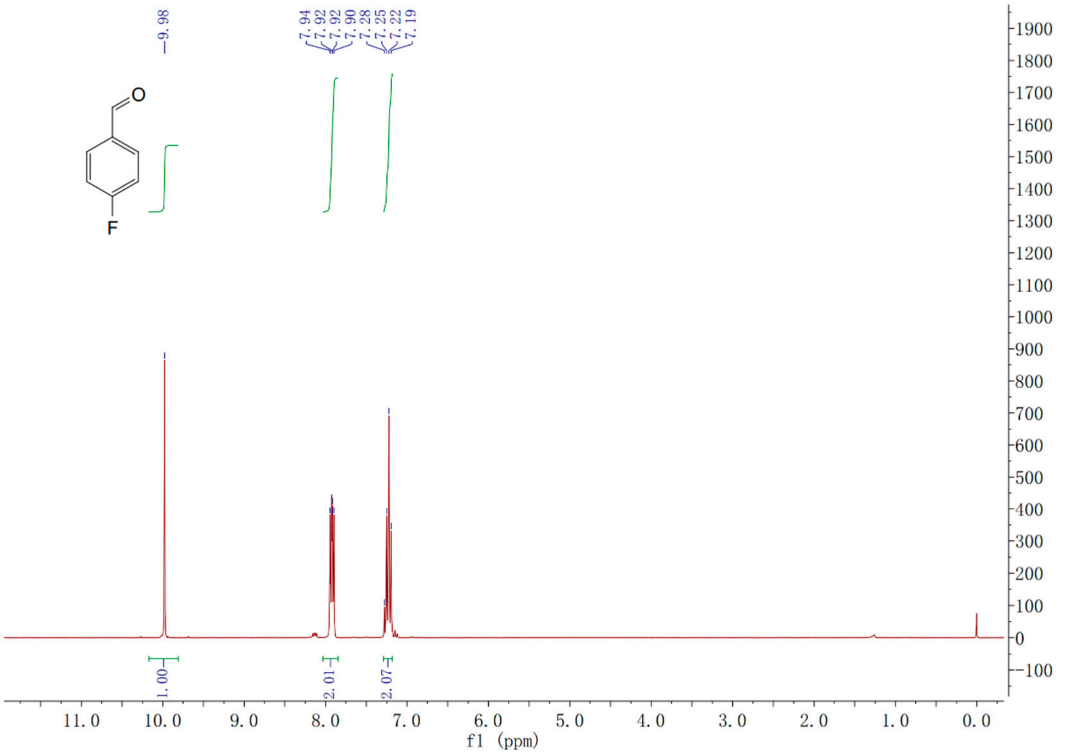

Figure S27. ^{1}H NMR spectrum of 2h in $CDCl_3$ at room temperature

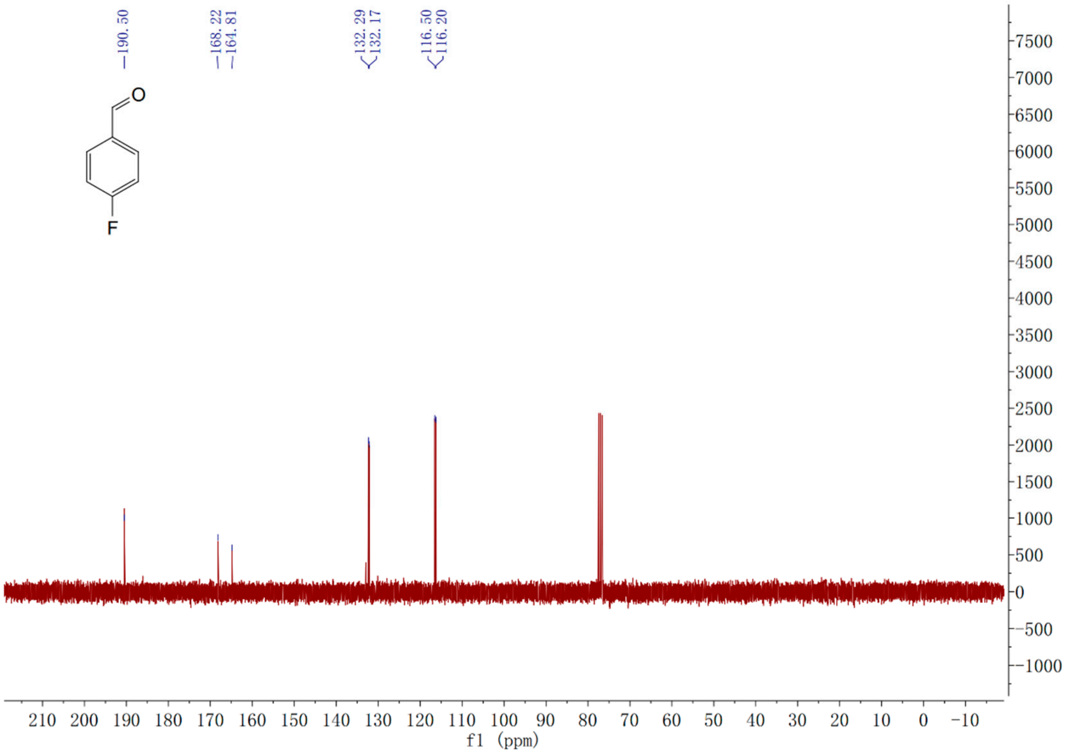

Figure S28. ^{13}C NMR spectrum of 2h in $CDCl_3$ at room temperature

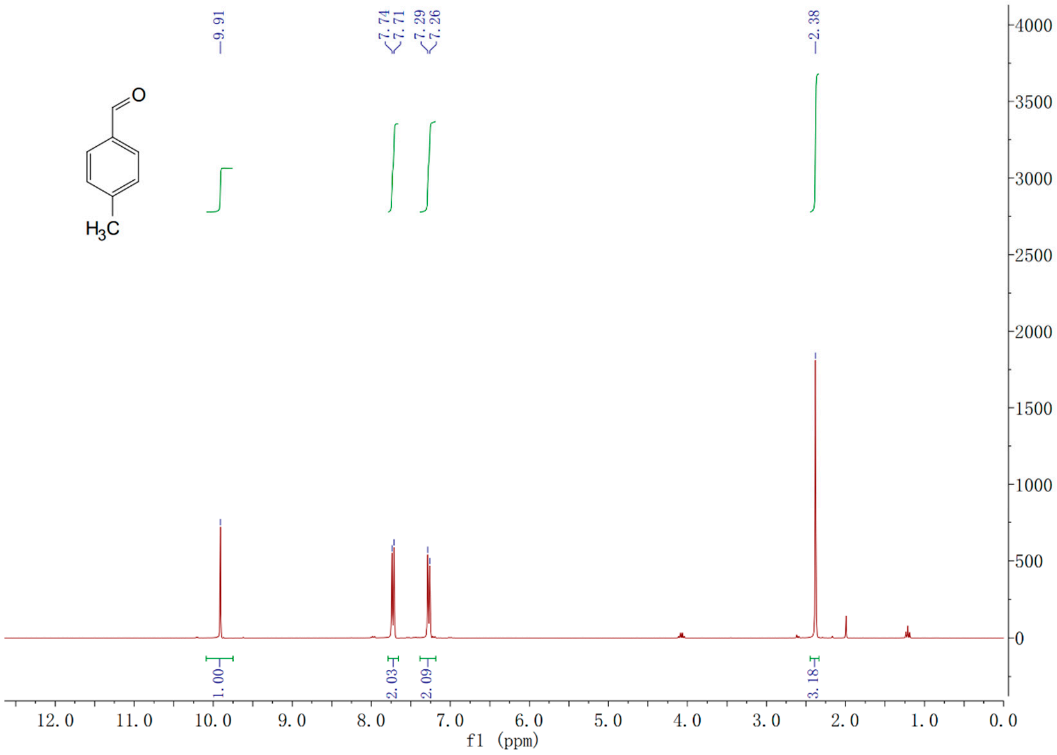

Figure S29. ^{1}H NMR spectrum of 2i in $CDCl_3$ at room temperature

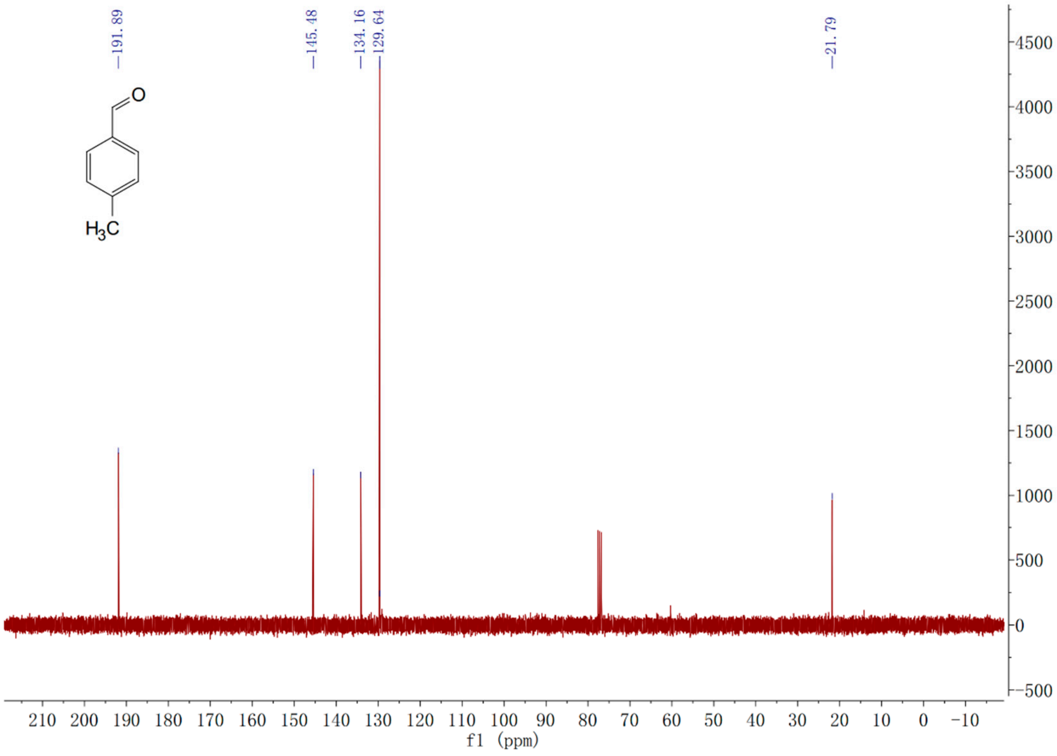

Figure S30. ^{13}C NMR spectrum of 2i in $CDCl_3$ at room temperature

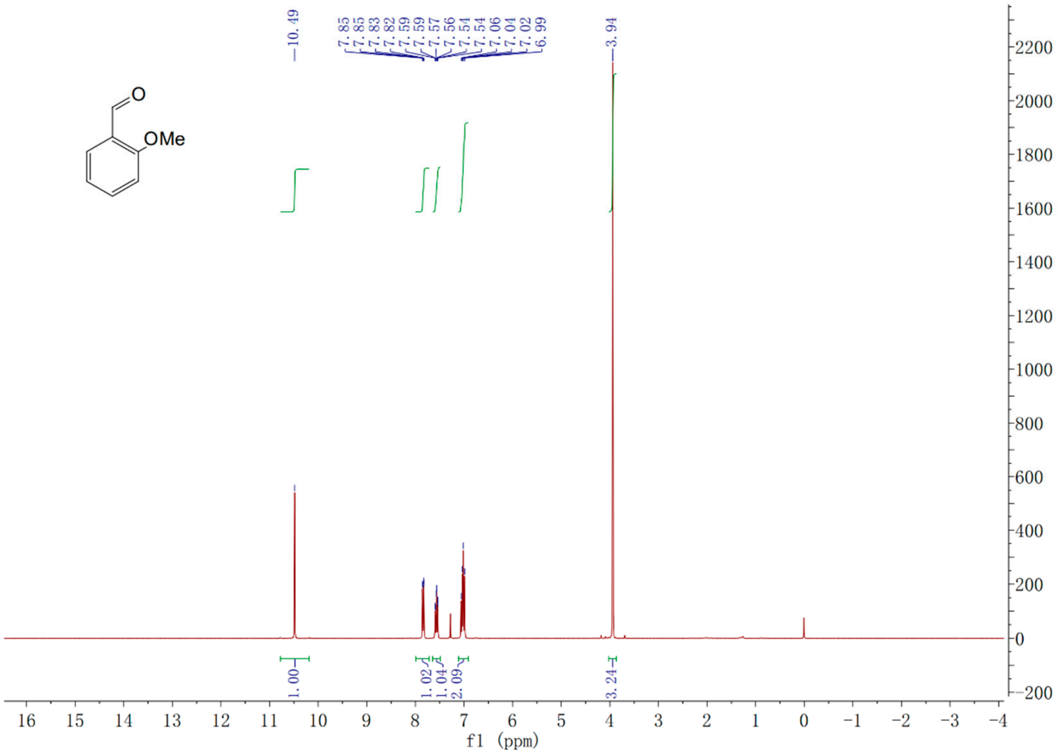

Figure S31. ^{1}H NMR spectrum of 2j in $CDCl_3$ at room temperature

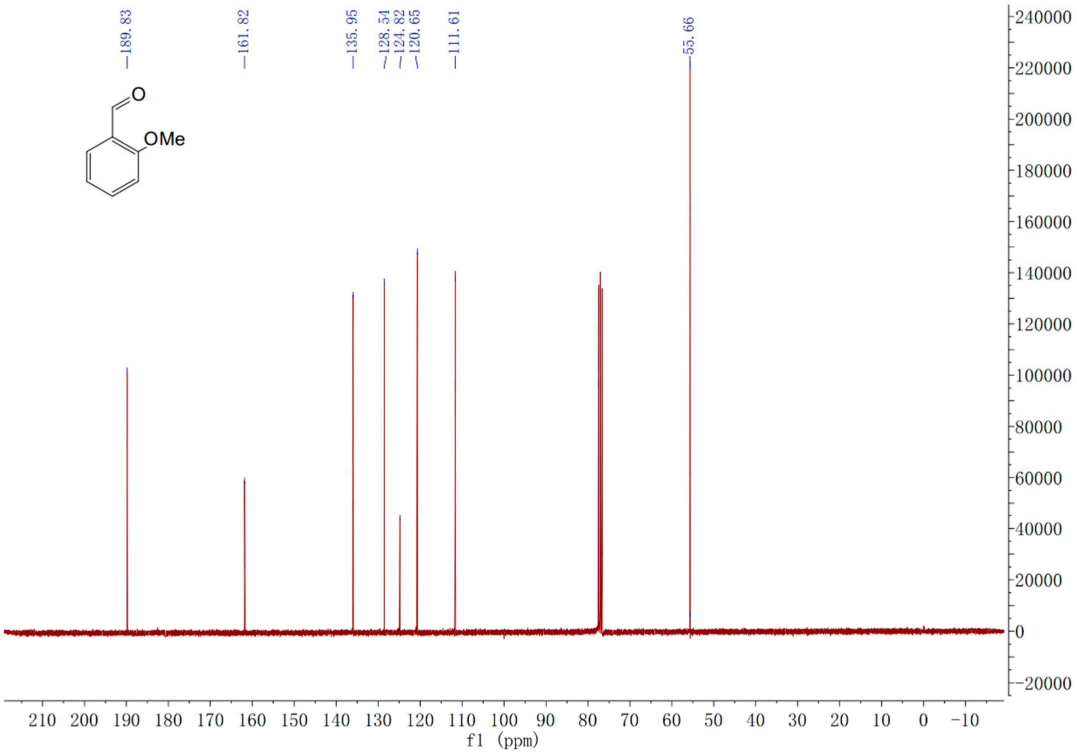

Figure S32. ^{13}C NMR spectrum of 2j in $CDCl_3$ at room temperature

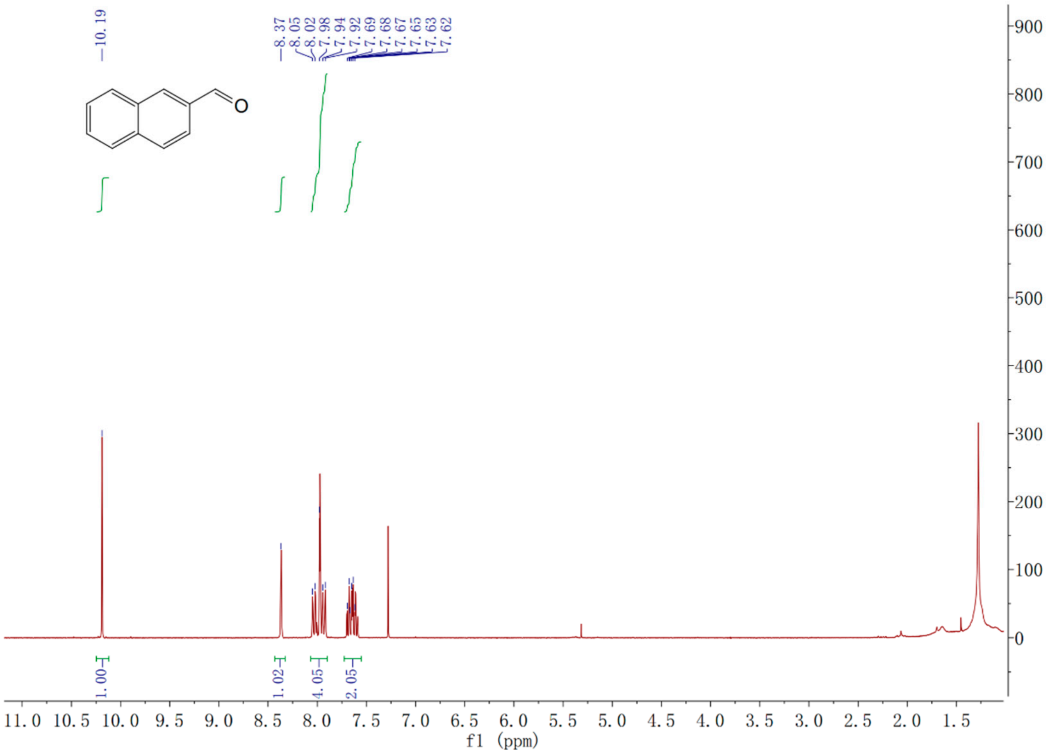

Figure S33. ^{1}H NMR spectrum of 2k in $CDCl_3$ at room temperature

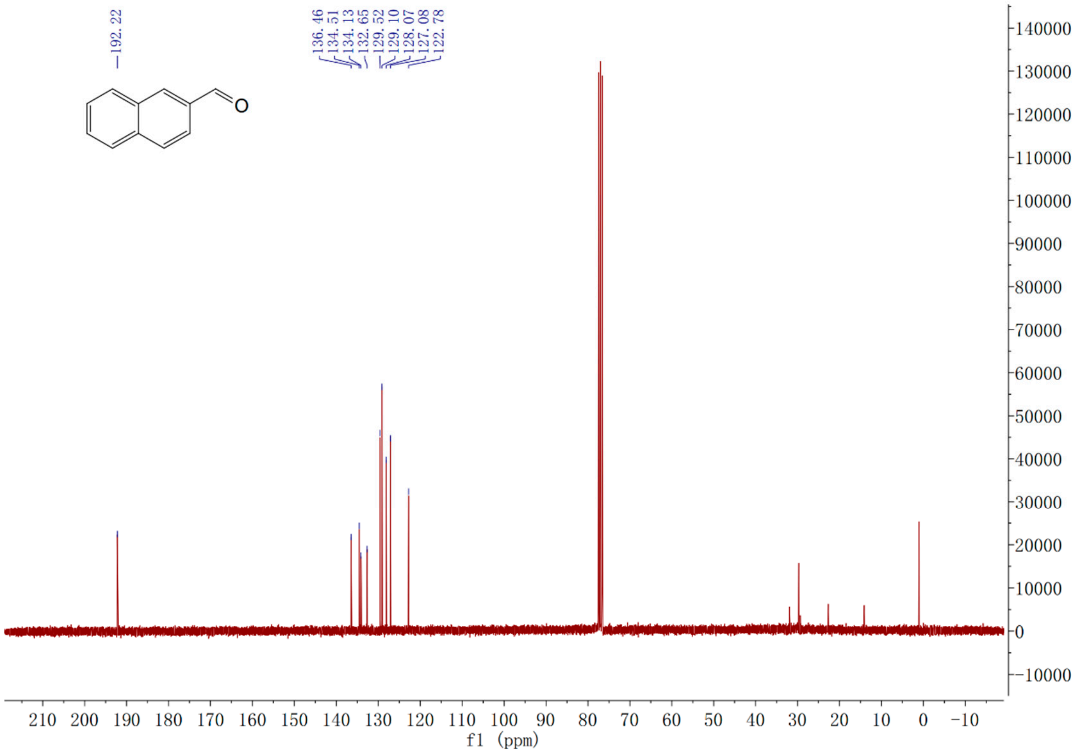

Figure S34. ^{13}C NMR spectrum of 2k in $CDCl_3$ at room temperature

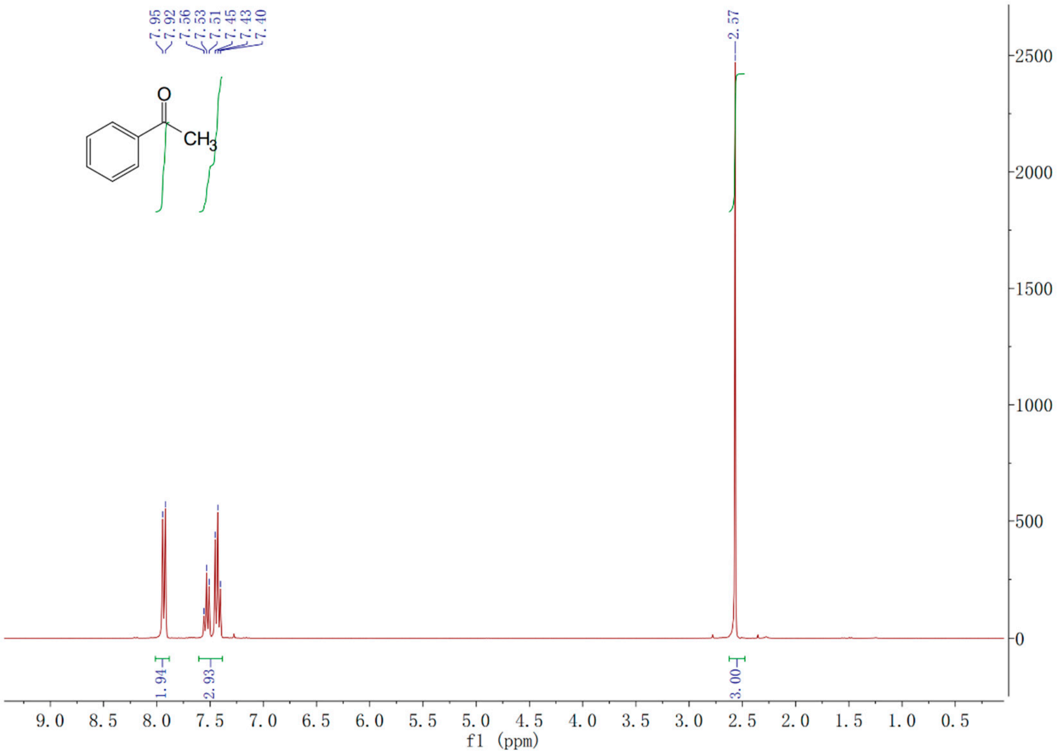

Figure S35. ^{1}H NMR spectrum of 2aa in $CDCl_3$ at room temperature

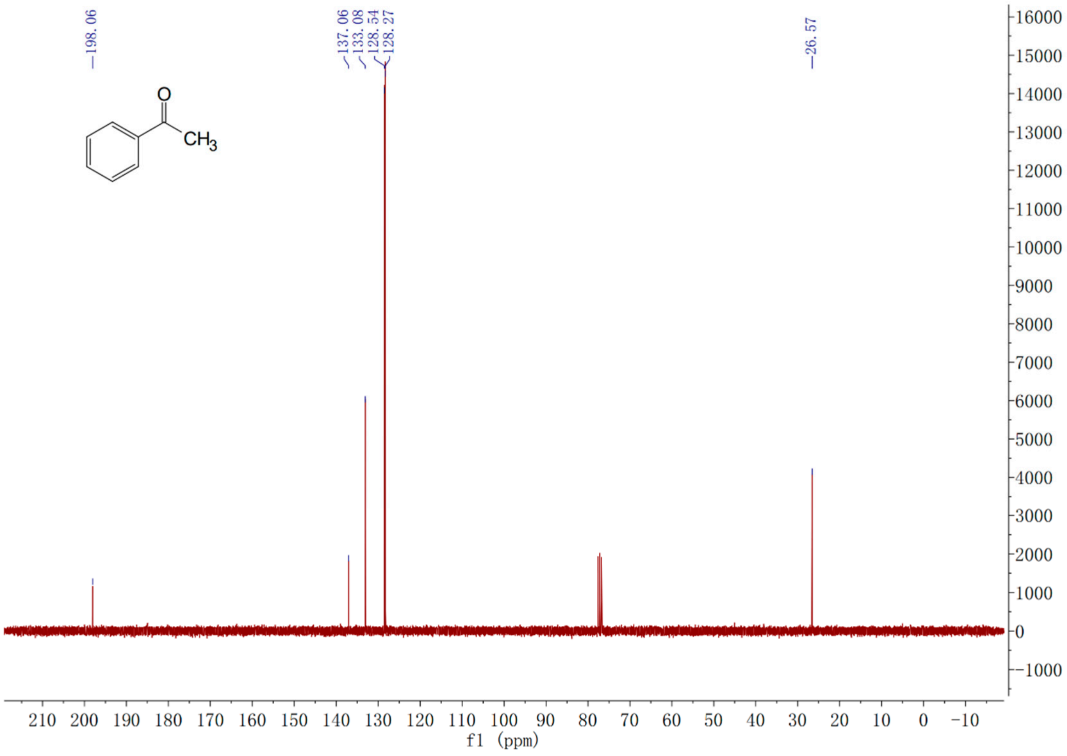

Figure S36. ^{13}C NMR spectrum of 2aa in $CDCl_3$ at room temperature

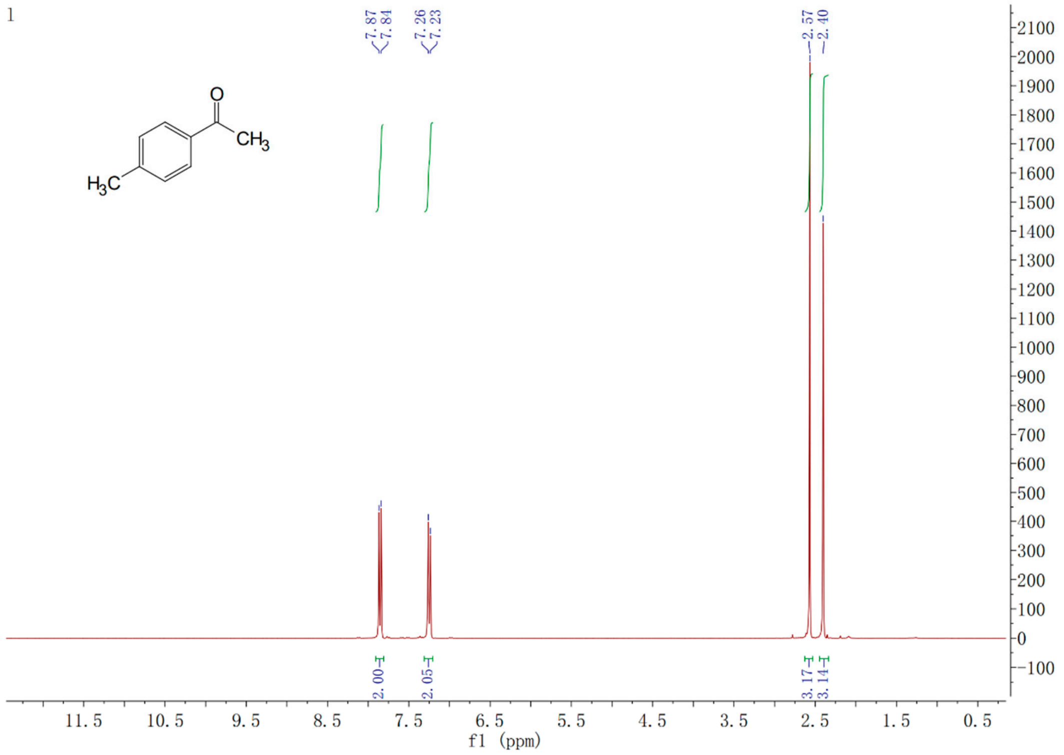

Figure S37. ^{1}H NMR spectrum of 2ab in $CDCl_3$ at room temperature

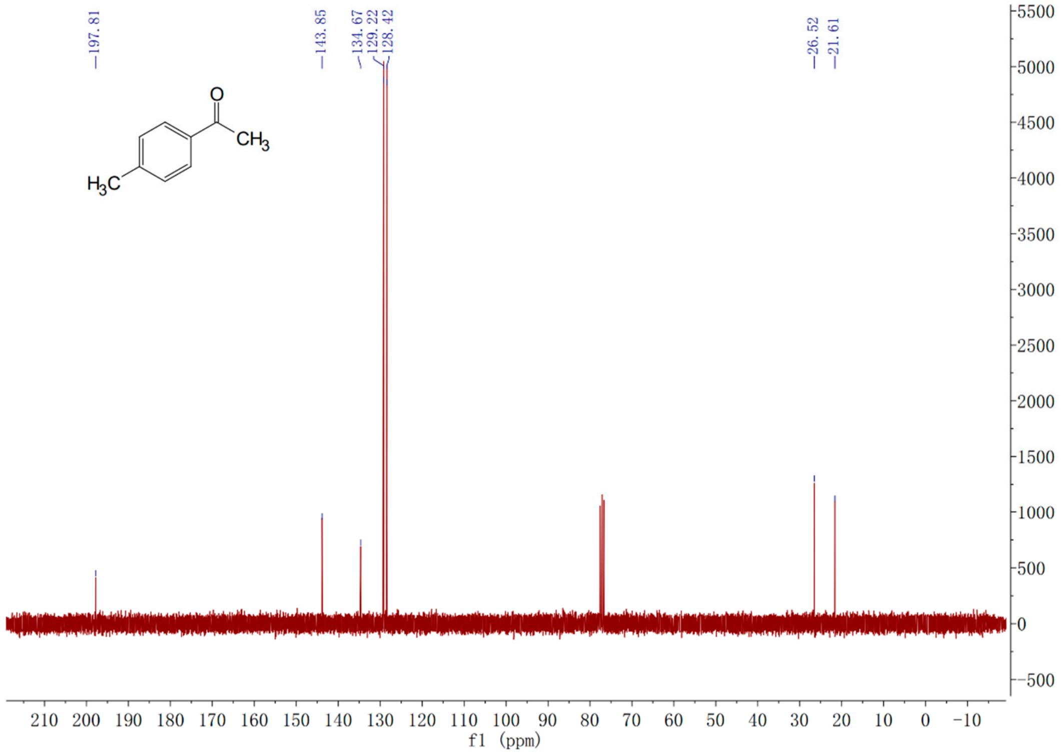

Figure S38. ^{13}C NMR spectrum of 2ab in $CDCl_3$ at room temperature

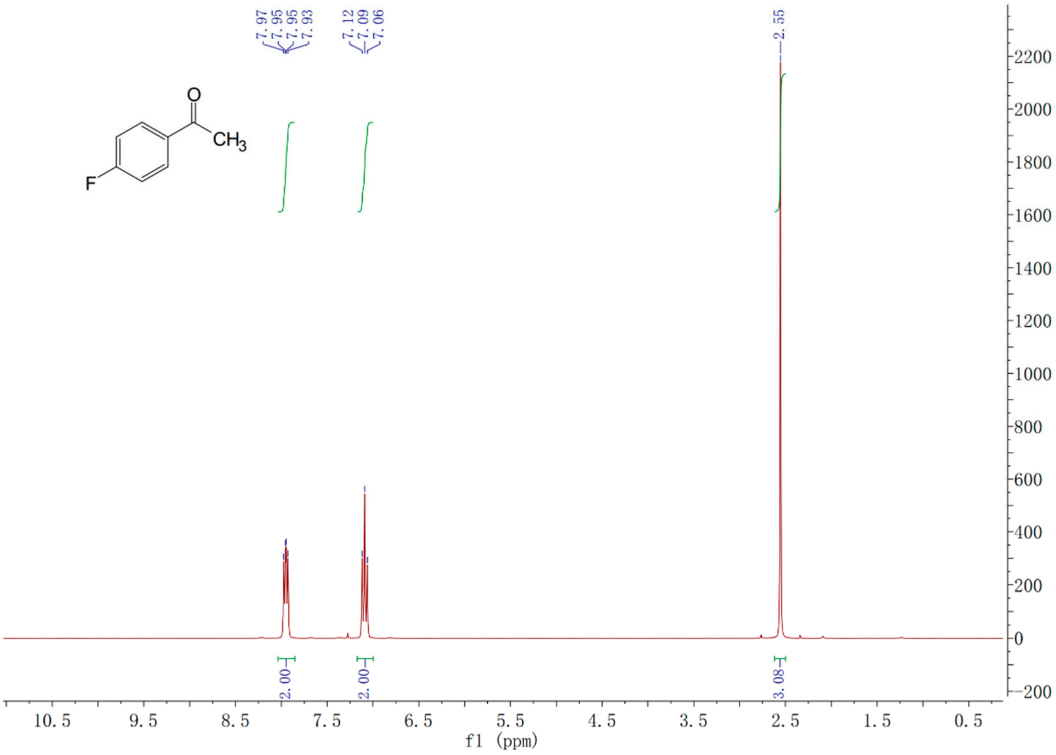

Figure S39. ^{1}H NMR spectrum of 2ac in $CDCl_3$ at room temperature

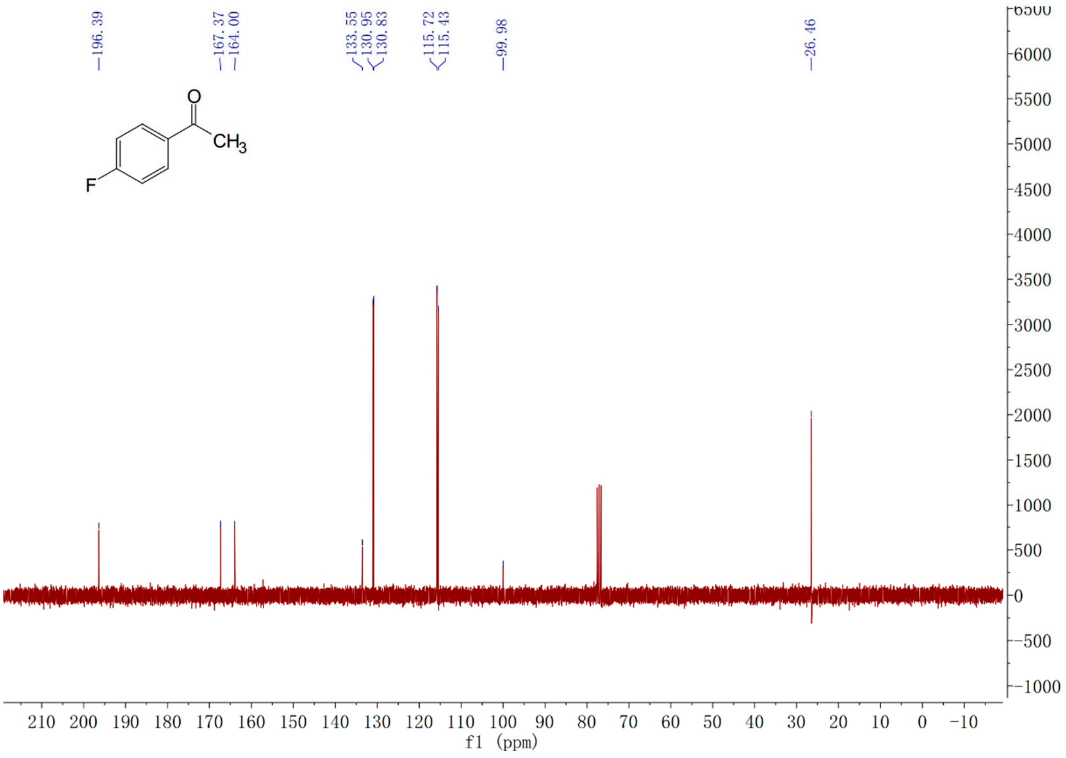

Figure S40. ^{13}C NMR spectrum of 2ac in $CDCl_3$ at room temperature

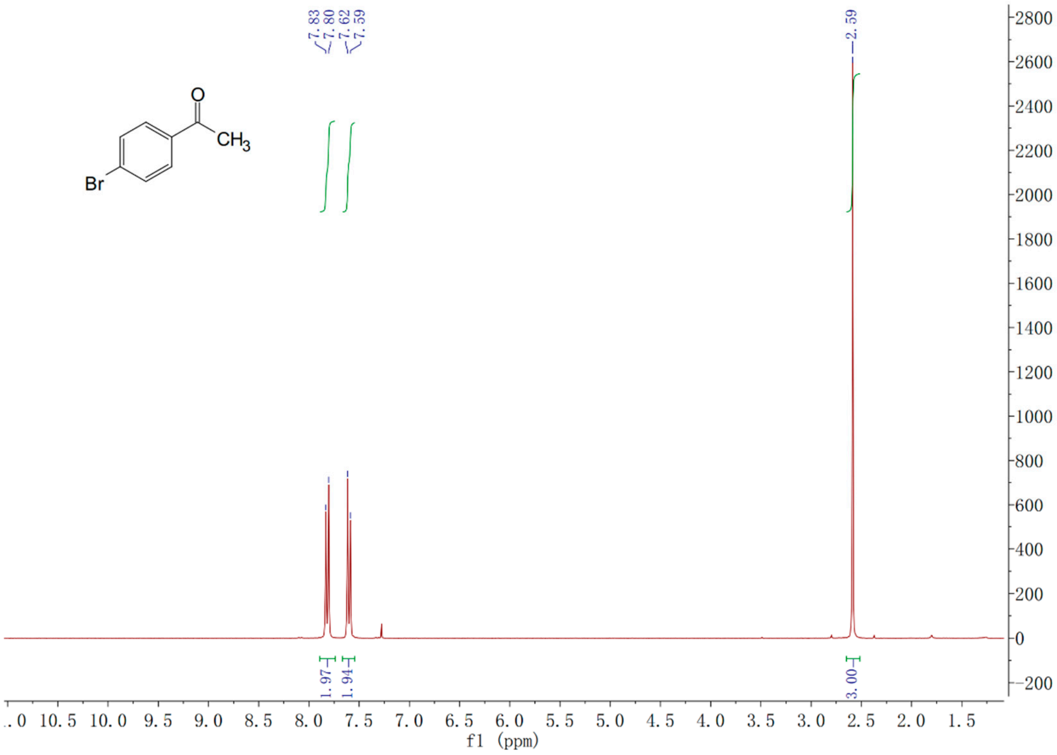

Figure S41. ^{1}H NMR spectrum of 2ad in $CDCl_3$ at room temperature

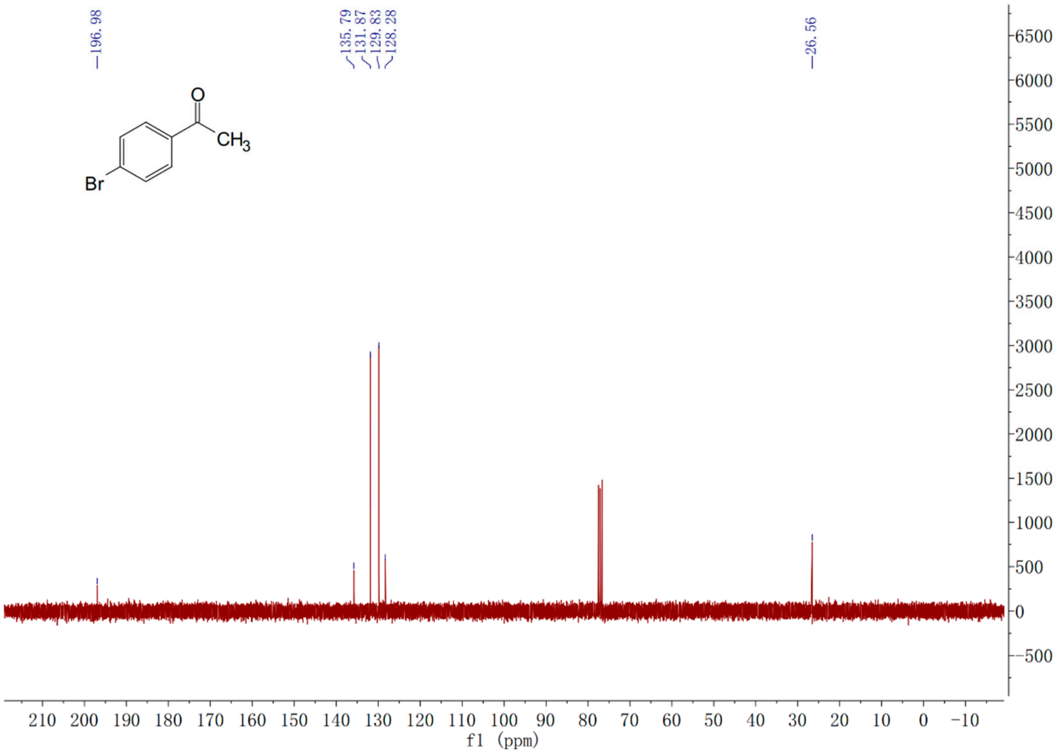

Figure S42. ^{13}C NMR spectrum of 2ad in $CDCl_3$ at room temperature

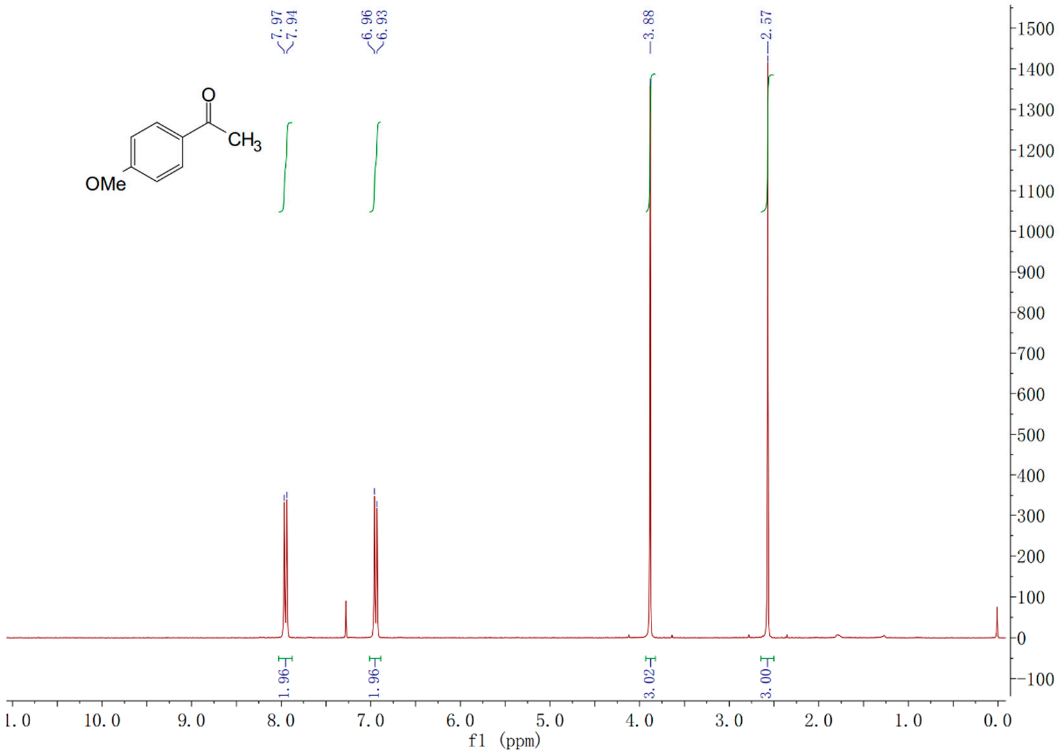

Figure S43. ^{1}H NMR spectrum of 2ae in $CDCl_3$ at room temperature

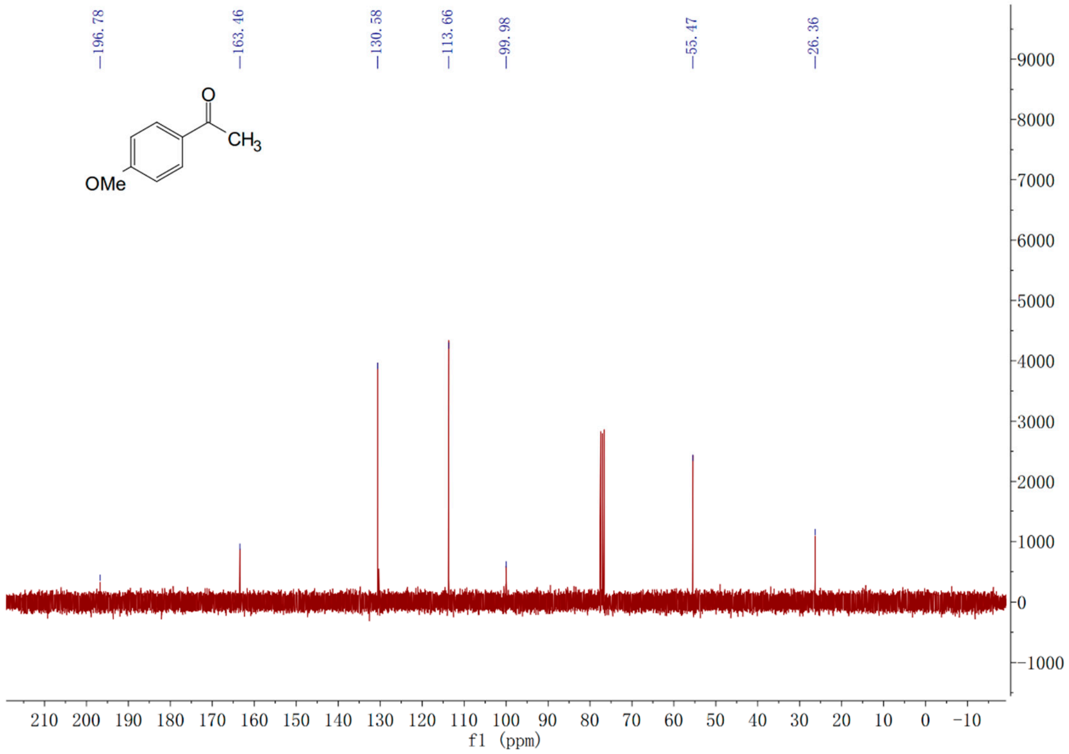

Figure S44. ^{13}C NMR spectrum of 2ae in $CDCl_3$ at room temperature

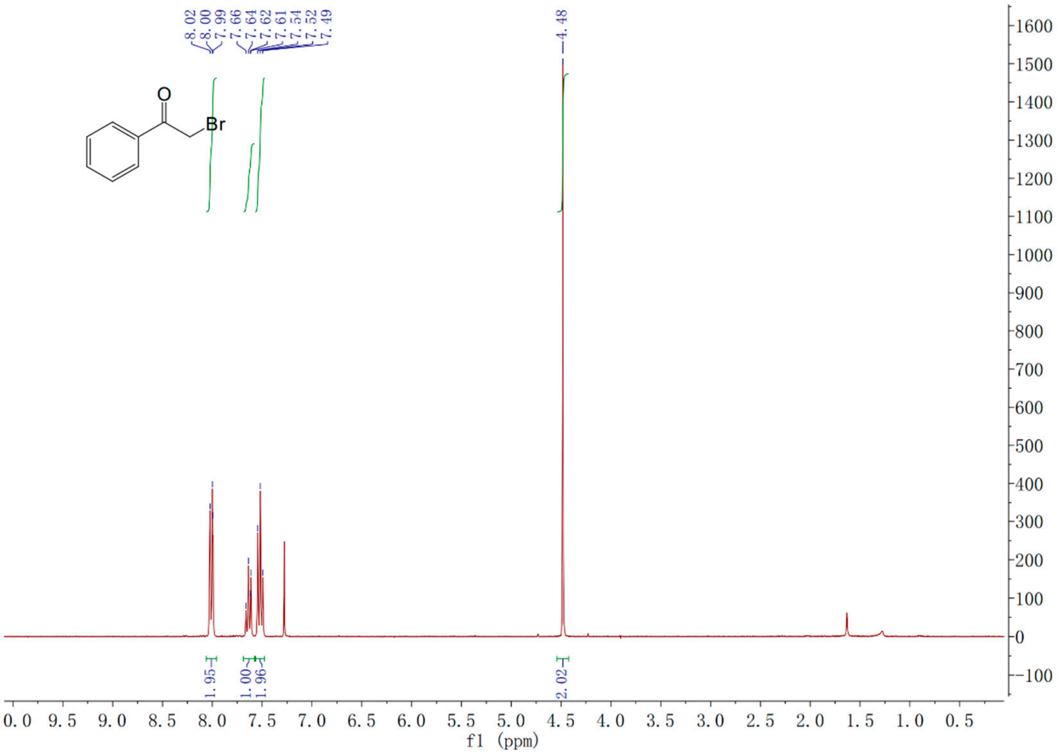

Figure S45. ^{1}H NMR spectrum of 2af in $CDCl_3$ at room temperature

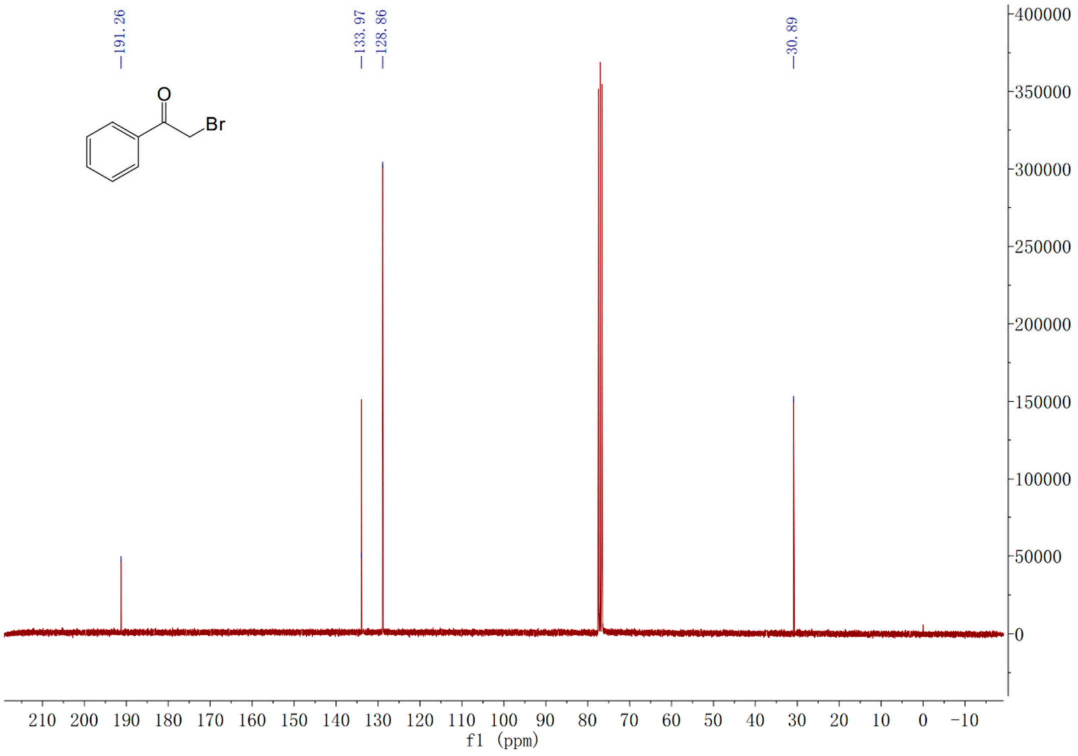

Figure S46. ^{13}C NMR spectrum of 2af in $CDCl_3$ at room temperature

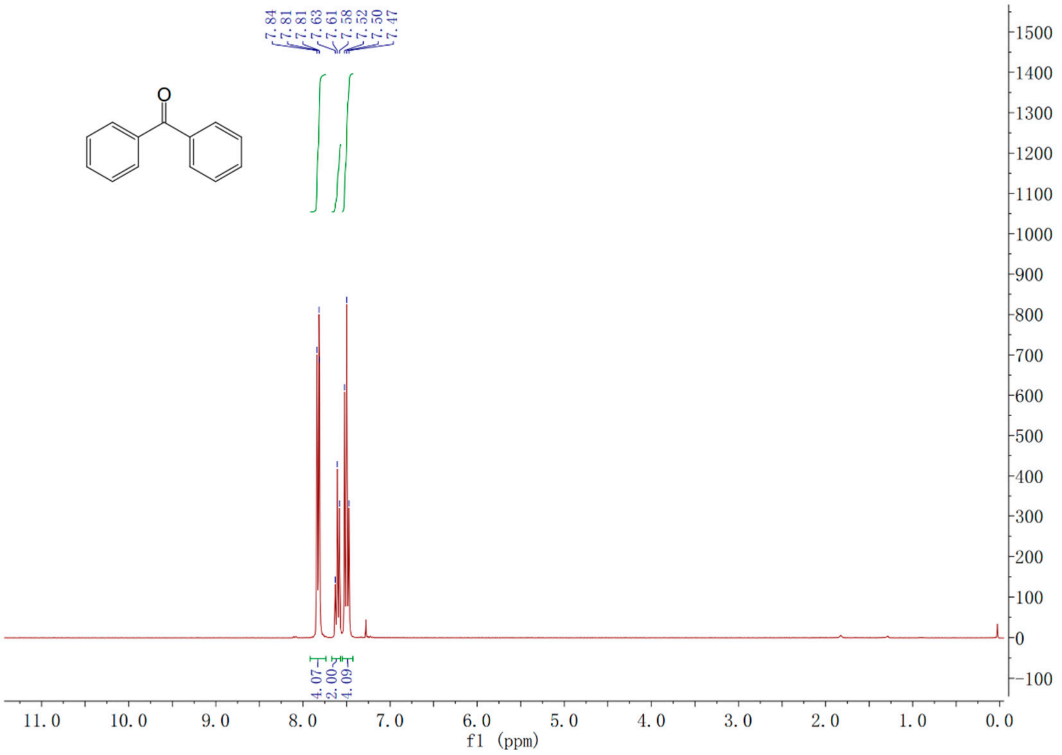

Figure S47. ^{1}H NMR spectrum of 2ag in $CDCl_3$ at room temperature

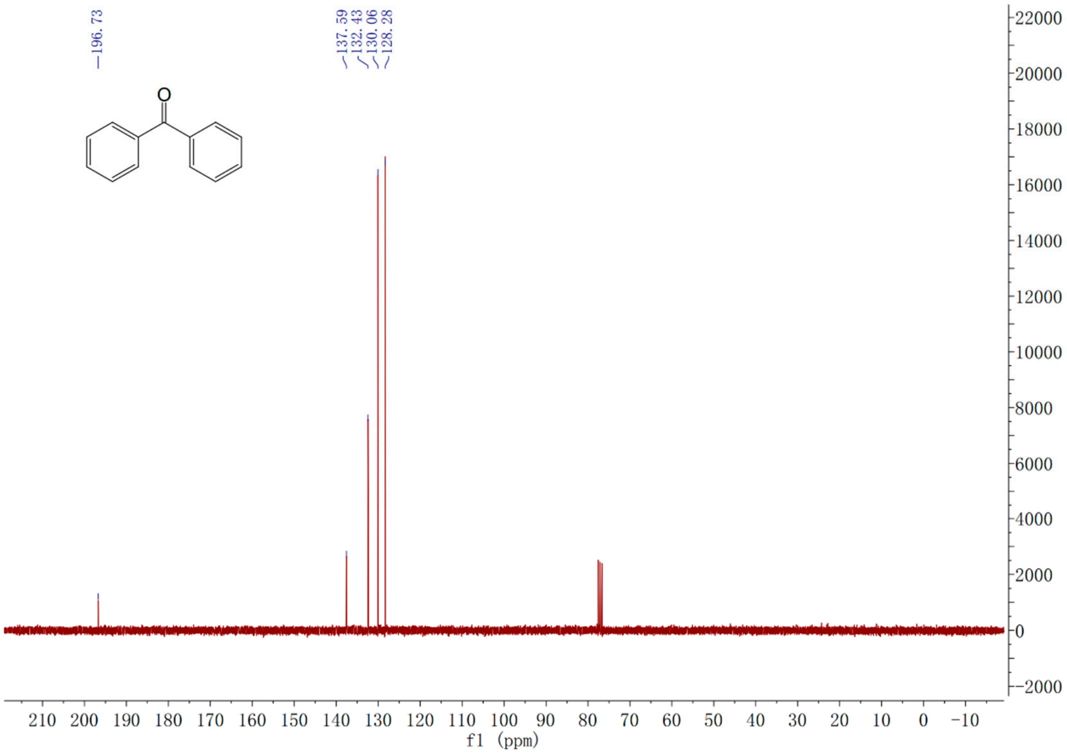

Figure S48. ^{13}C NMR spectrum of 2ag in $CDCl_3$ at room temperature

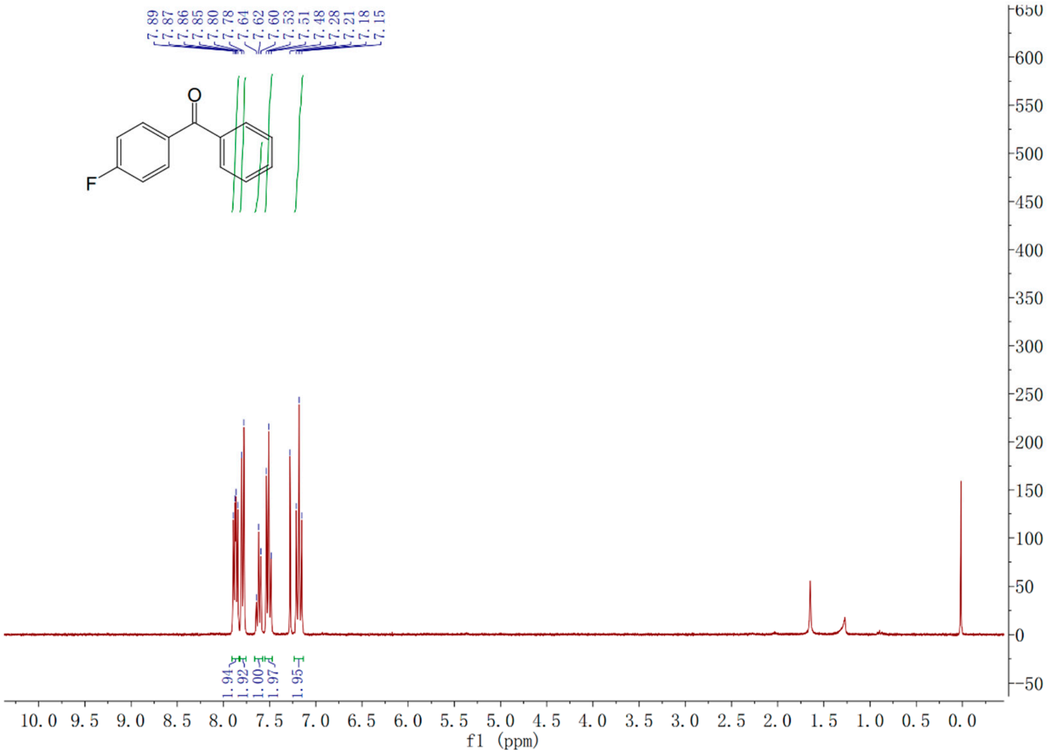

Figure S49. ^{1}H NMR spectrum of 2ah in $CDCl_3$ at room temperature

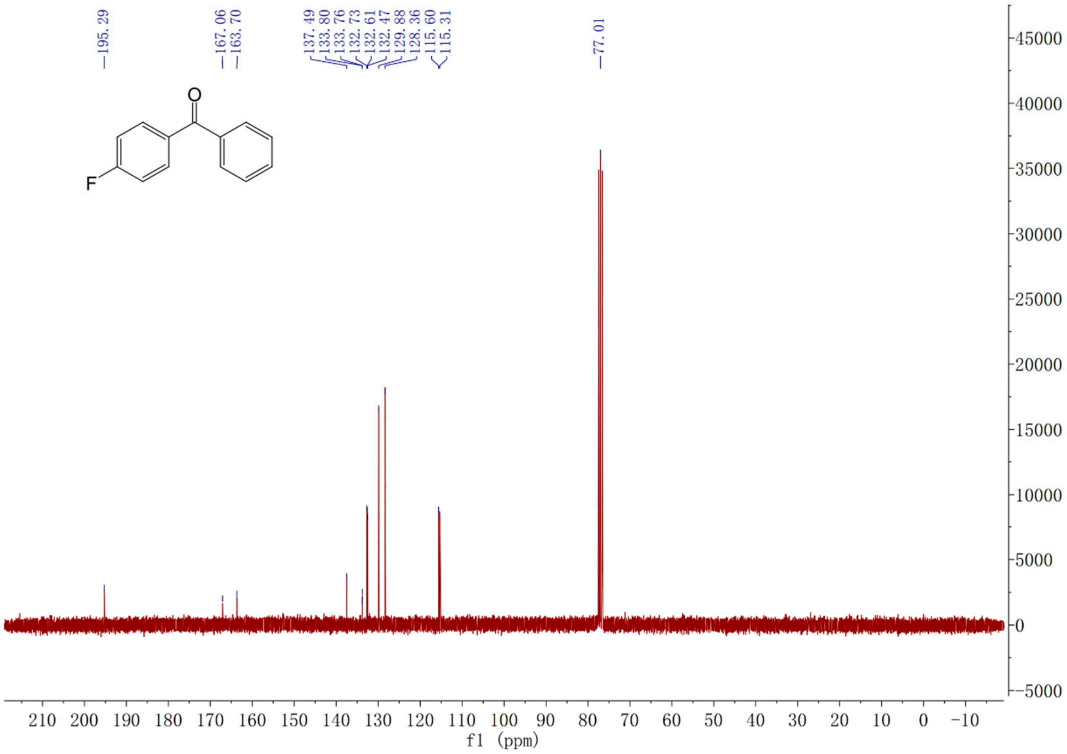

Figure S50. ^{13}C NMR spectrum of 2ah in $CDCl_3$ at room temperature

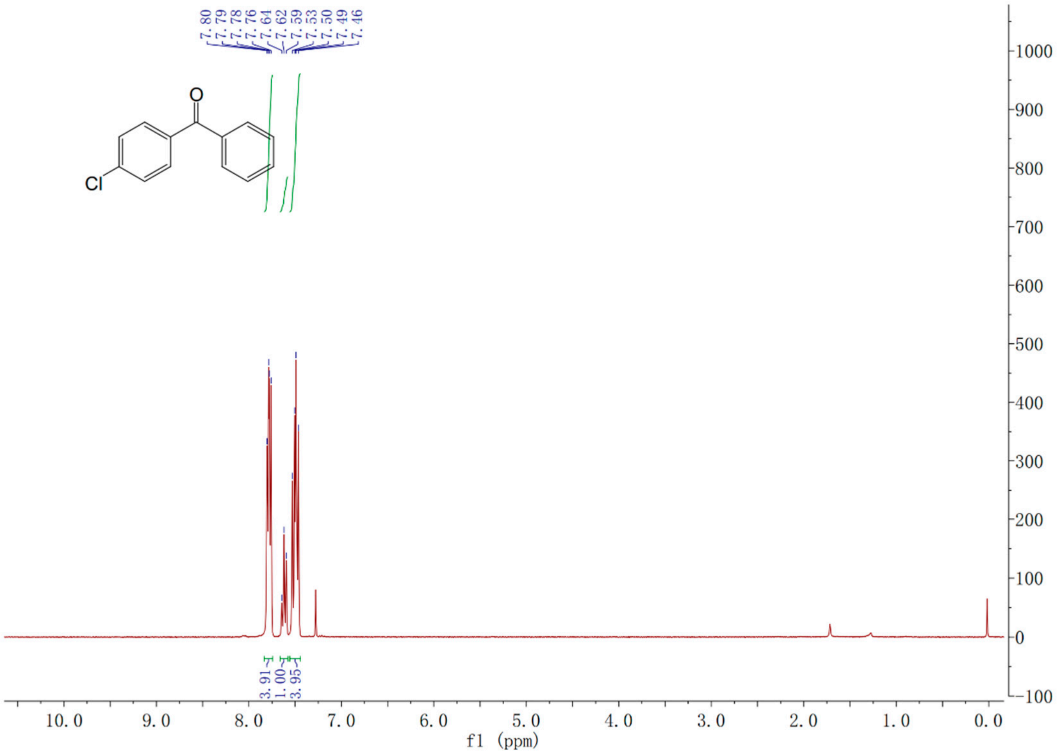

Figure S51. ^{1}H NMR spectrum of 2ai in $CDCl_3$ at room temperature

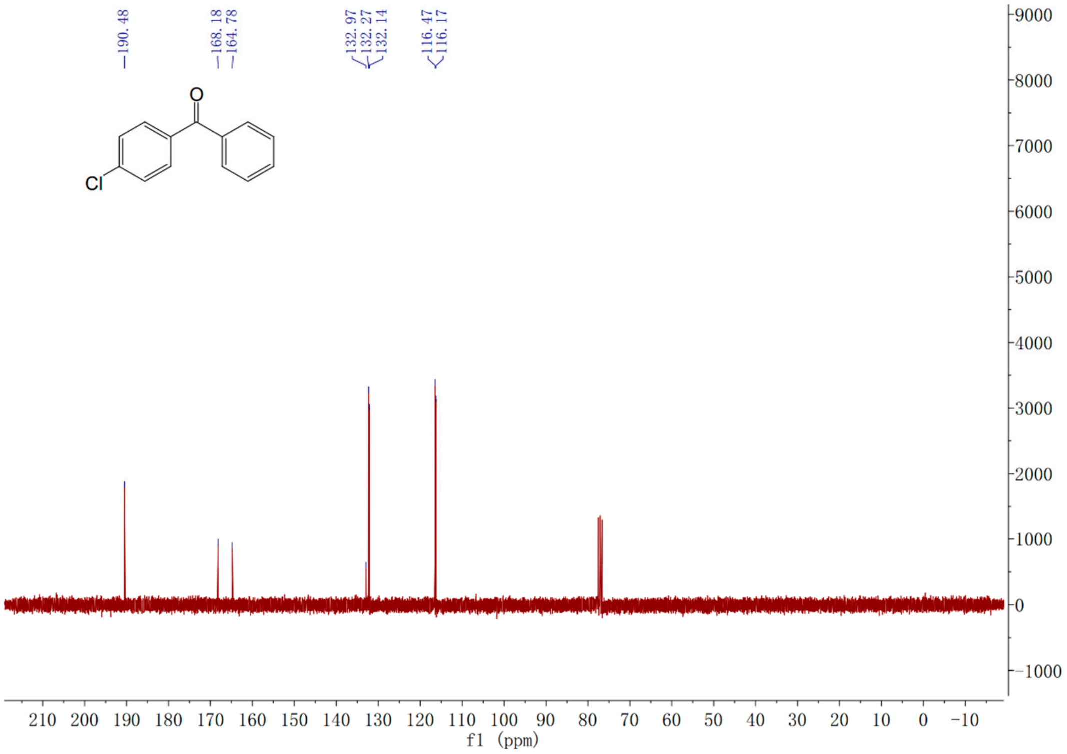

Figure S52. ^{13}C NMR spectrum of 2ai in $CDCl_3$ at room temperature

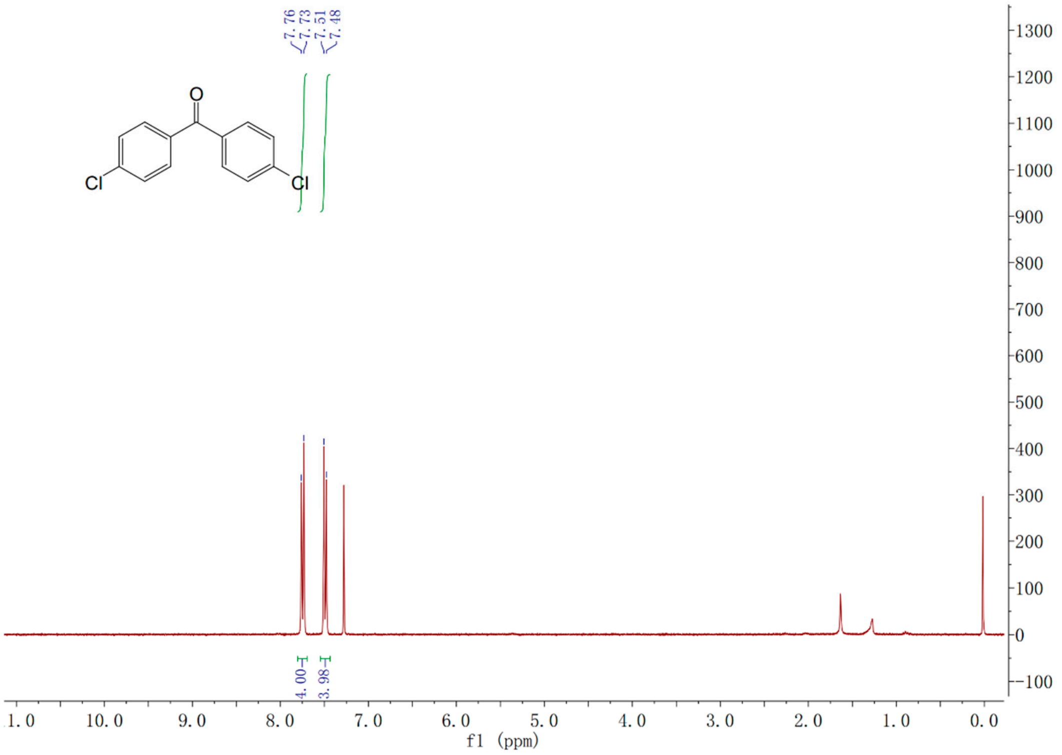

Figure S53. ^{1}H NMR spectrum of 2aj in $CDCl_3$ at room temperature

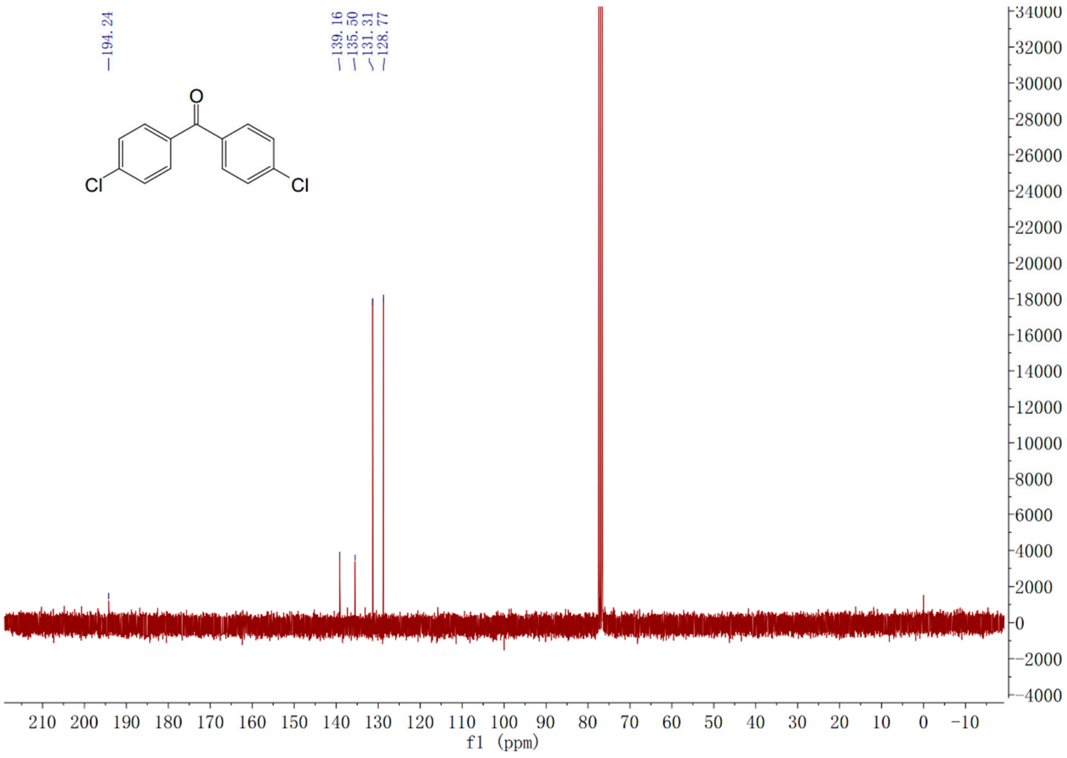

Figure S54. ^{13}C NMR spectrum of 2aj in $CDCl_3$ at room temperature

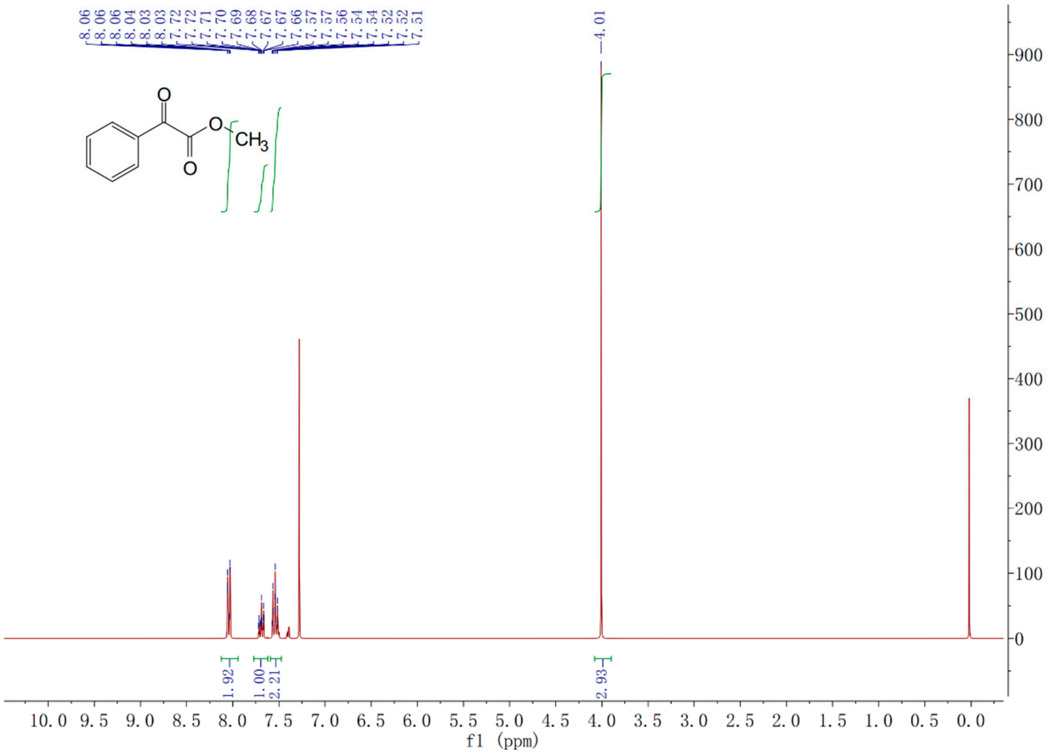

Figure S55. ^{1}H NMR spectrum of 2ba in $CDCl_3$ at room temperature

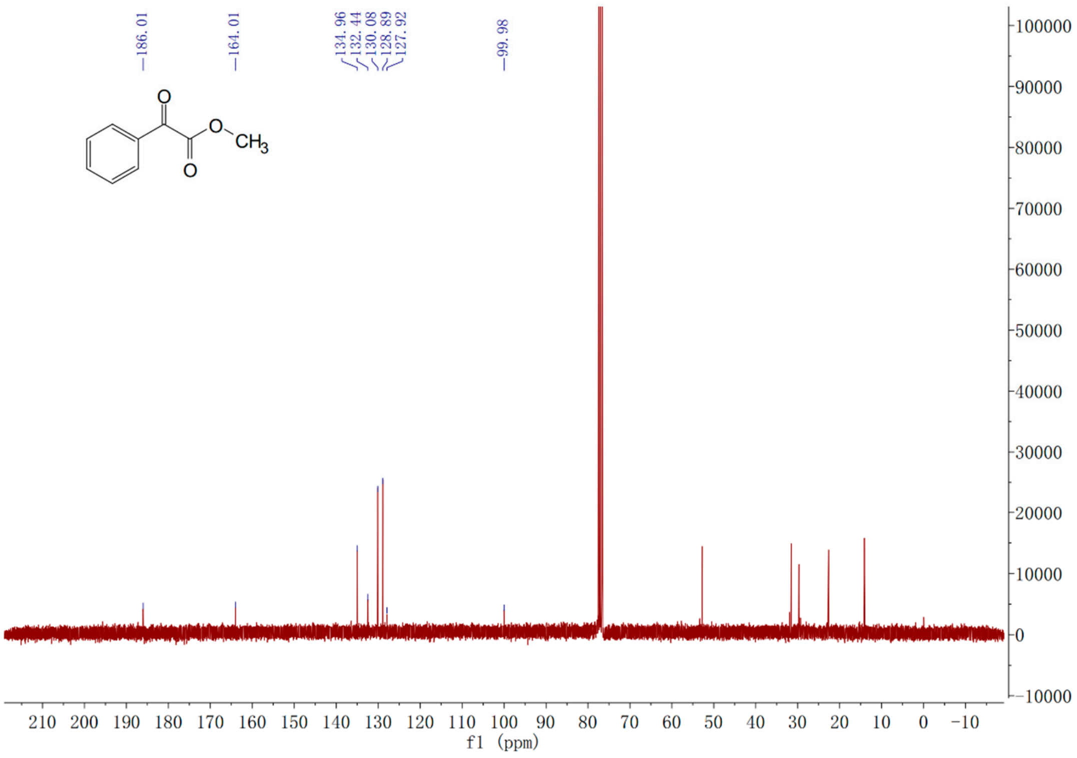

Figure S56. ^{13}C NMR spectrum of 2ba in $CDCl_3$ at room temperature

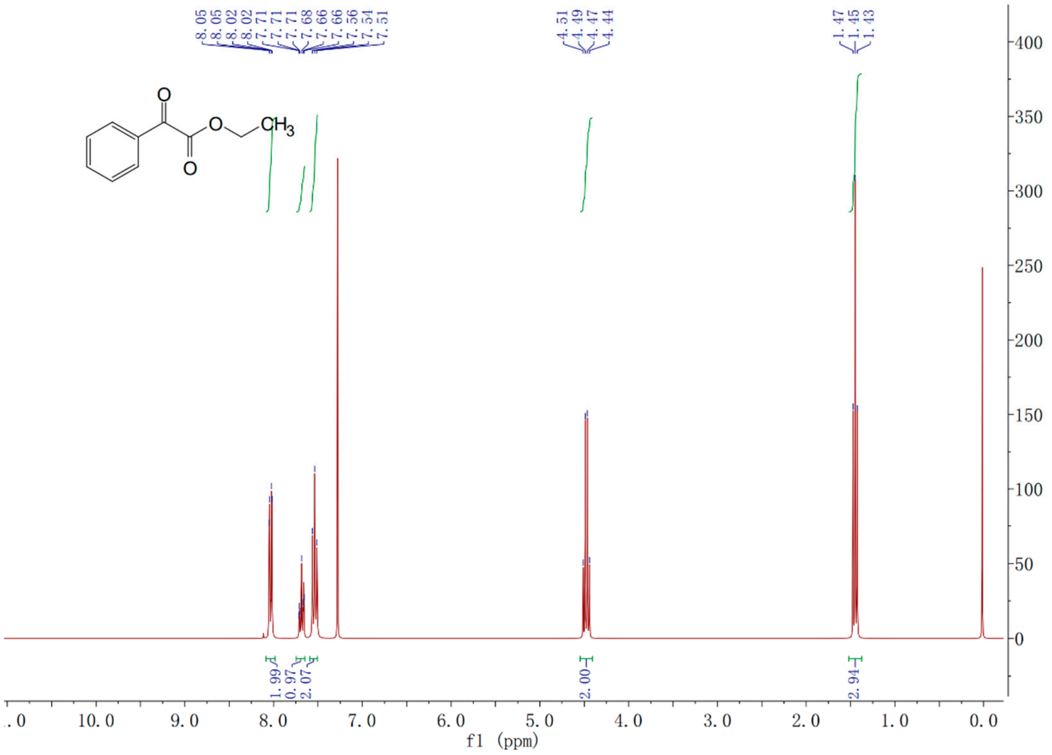

Figure S57. ^{1}H NMR spectrum of 2bb in $CDCl_3$ at room temperature

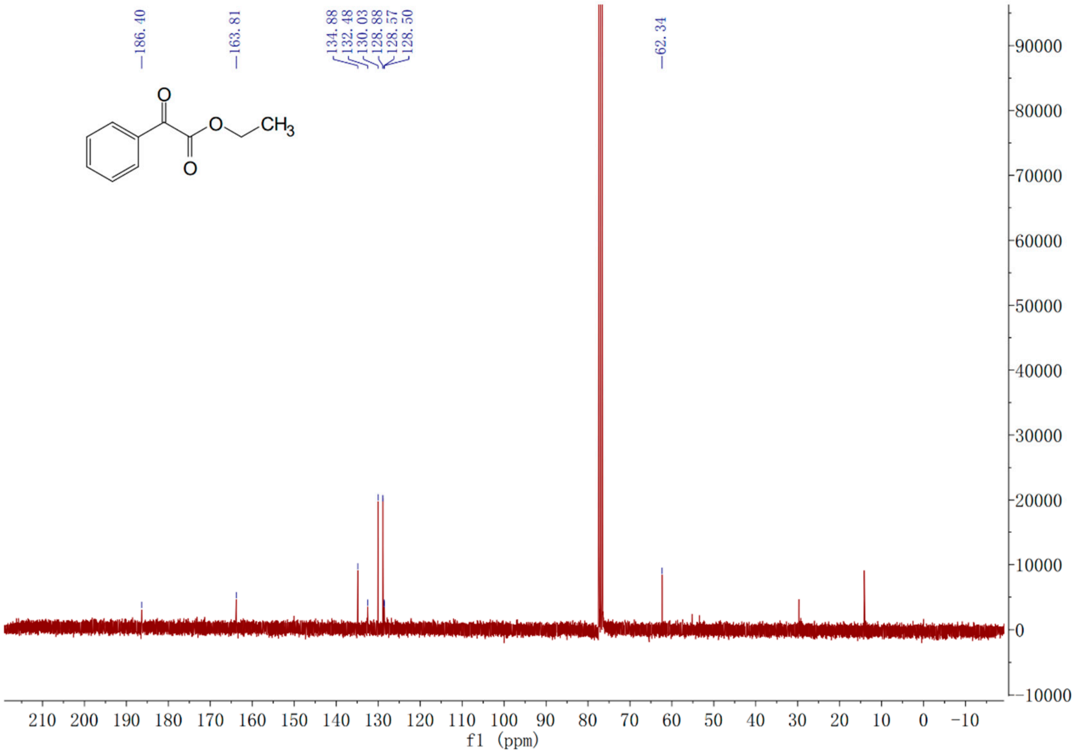

Figure S58. ^{13}C NMR spectrum of 2bb in $CDCl_3$ at room temperature

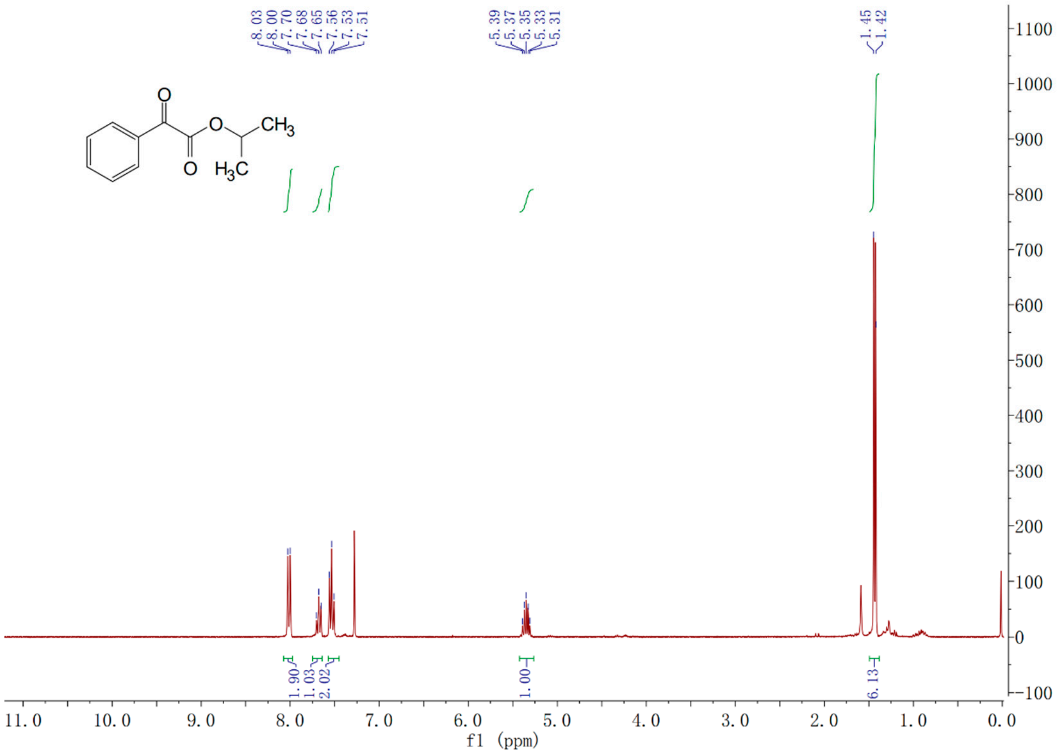

Figure S59. ^{1}H NMR spectrum of 2bc in $CDCl_3$ at room temperature

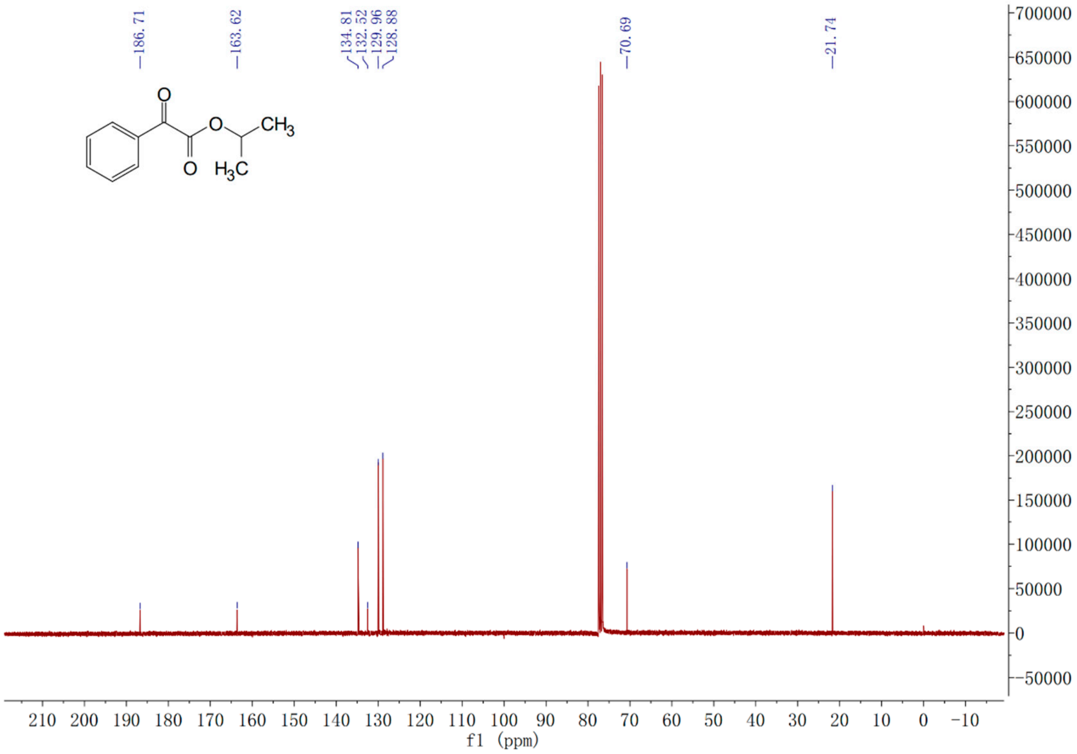

Figure S60. ^{13}C NMR spectrum of 2bc in $CDCl_3$ at room temperature

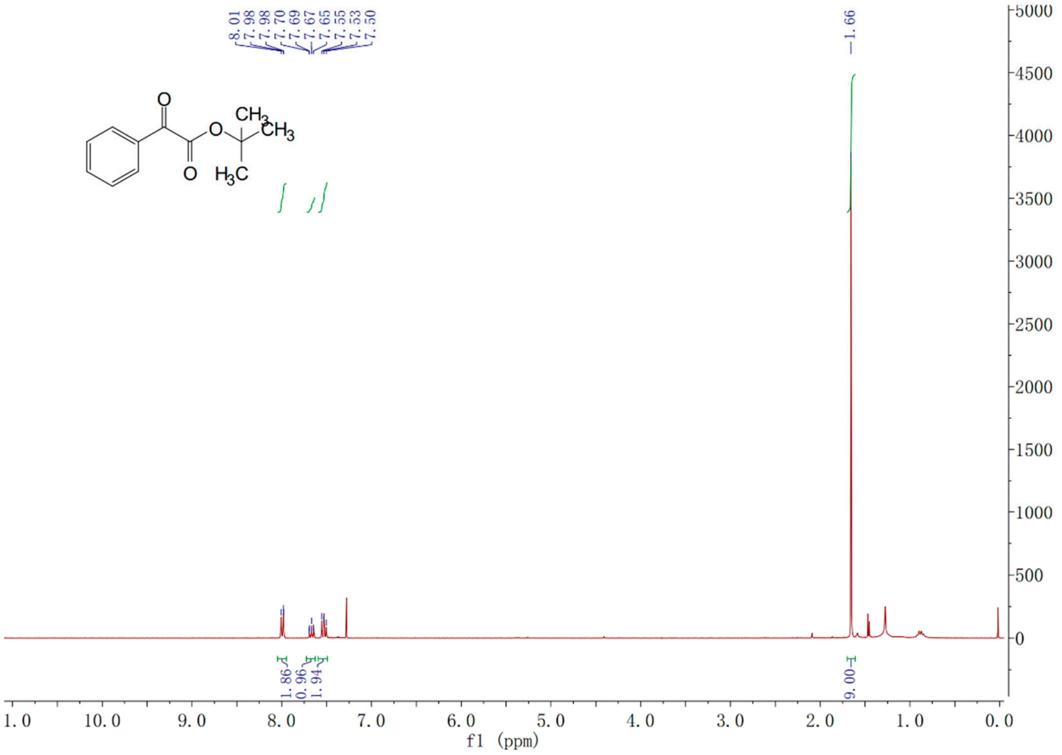

Figure S61. ^{1}H NMR spectrum of 2bd in $CDCl_3$ at room temperature

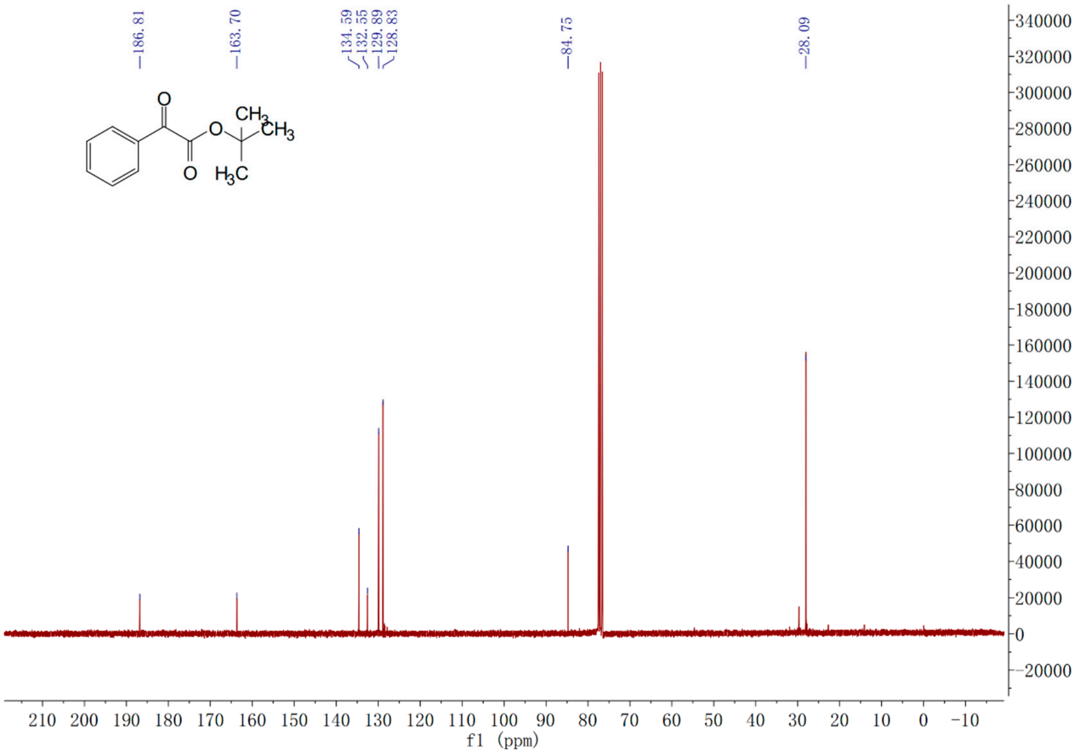

Figure S62. ^{13}C NMR spectrum of 2bd in $CDCl_3$ at room temperature

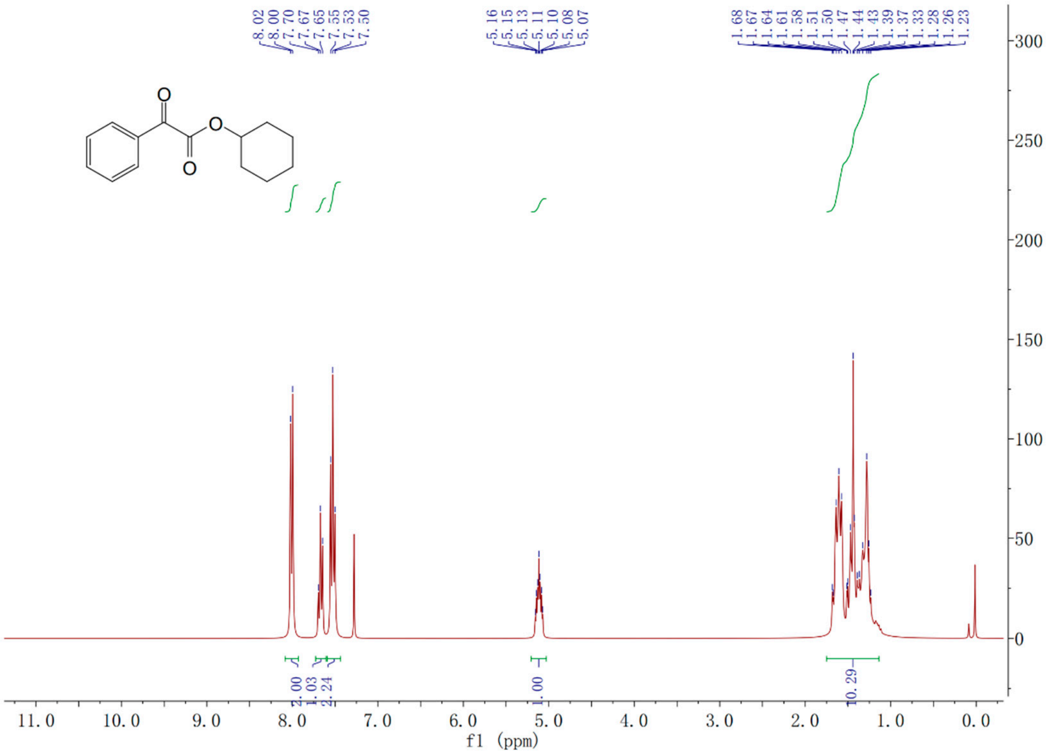

Figure S63. ^{1}H NMR spectrum of 2be in $CDCl_3$ at room temperature

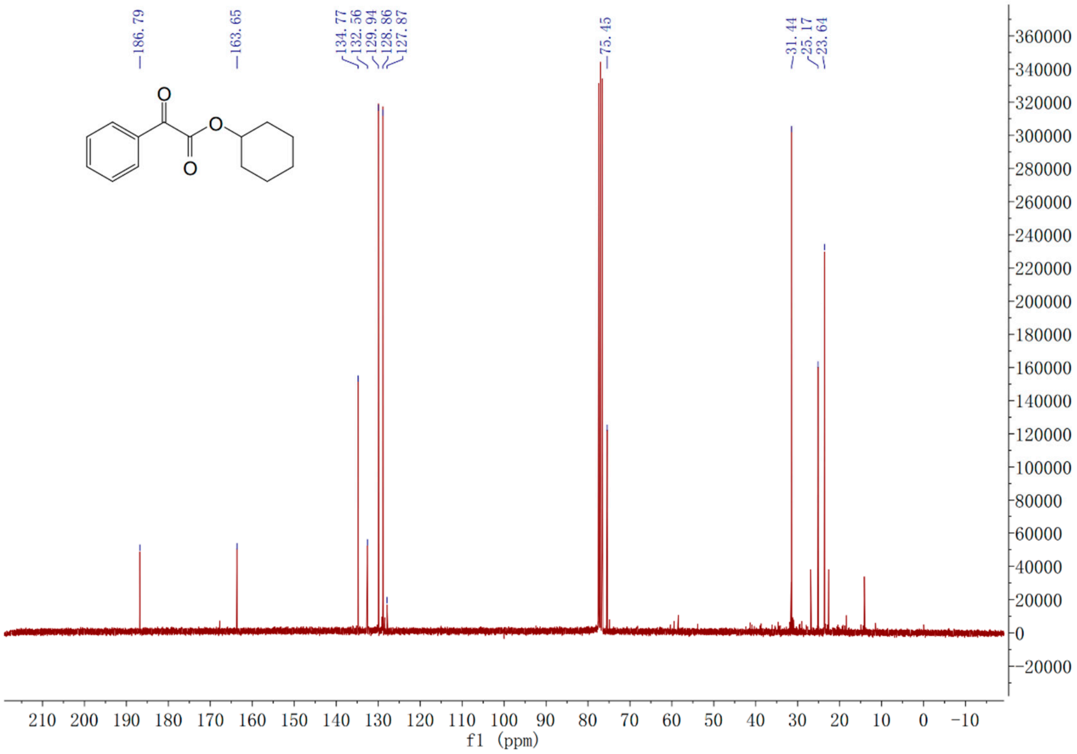

Figure S64. ^{13}C NMR spectrum of 2be in $CDCl_3$ at room temperature

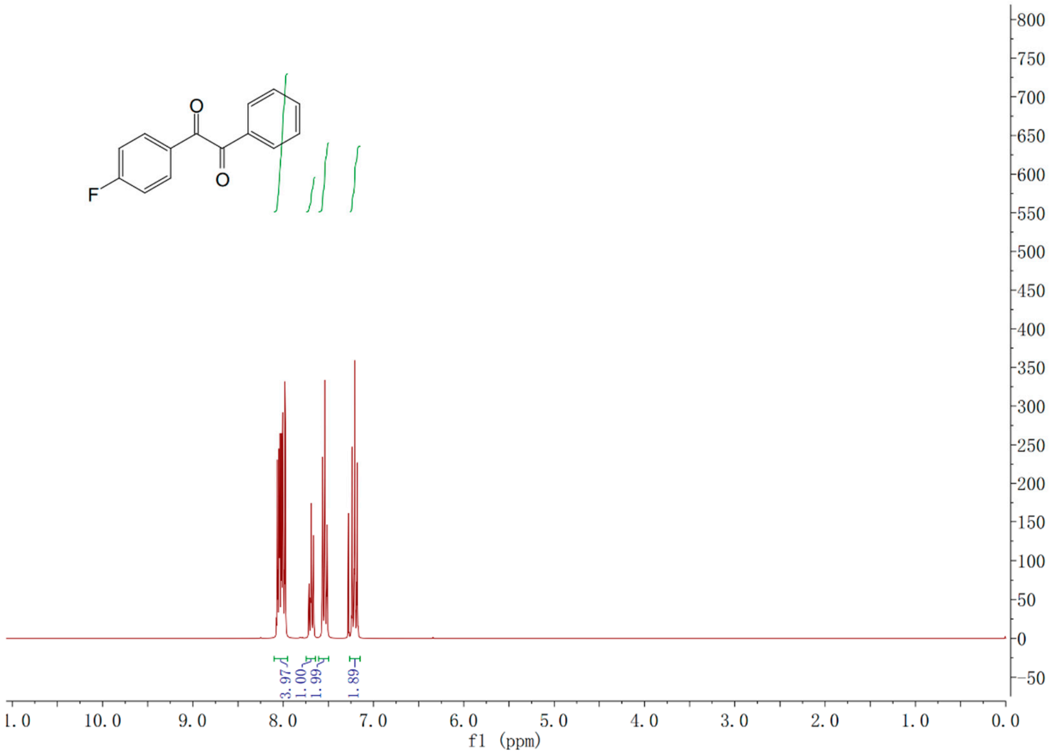

Figure S65. ^{1}H NMR spectrum of 2bf in $CDCl_3$ at room temperature

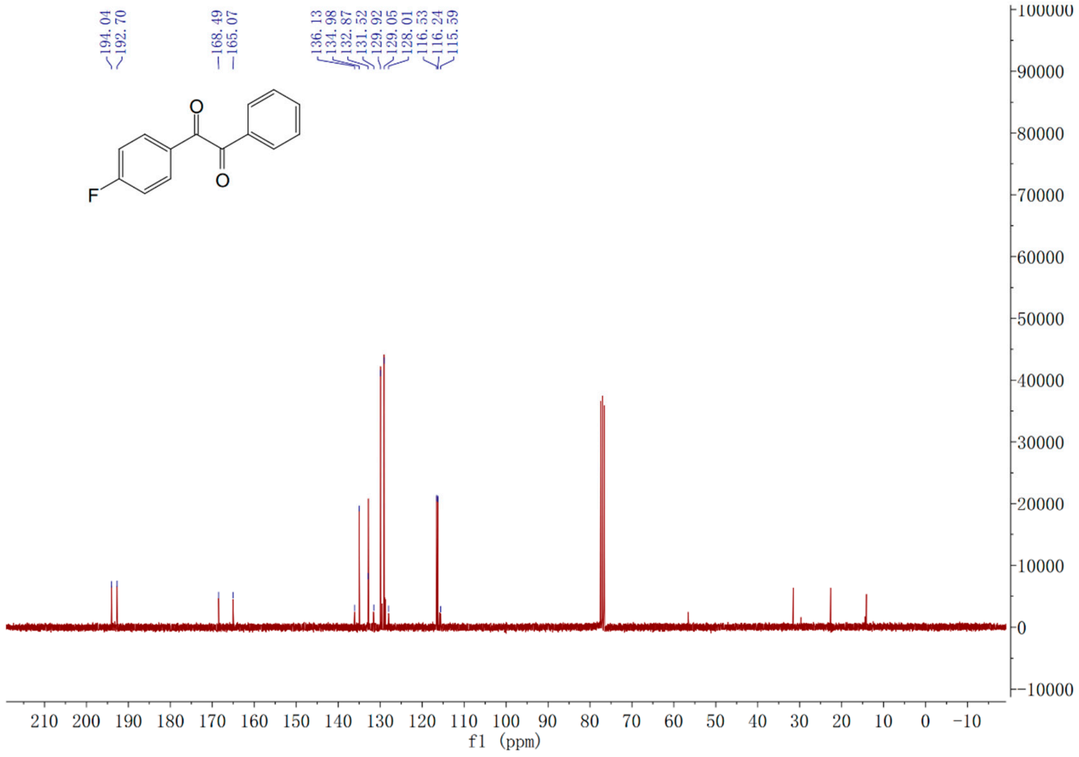

Figure S66. ^{13}C NMR spectrum of 2bf in $CDCl_3$ at room temperature

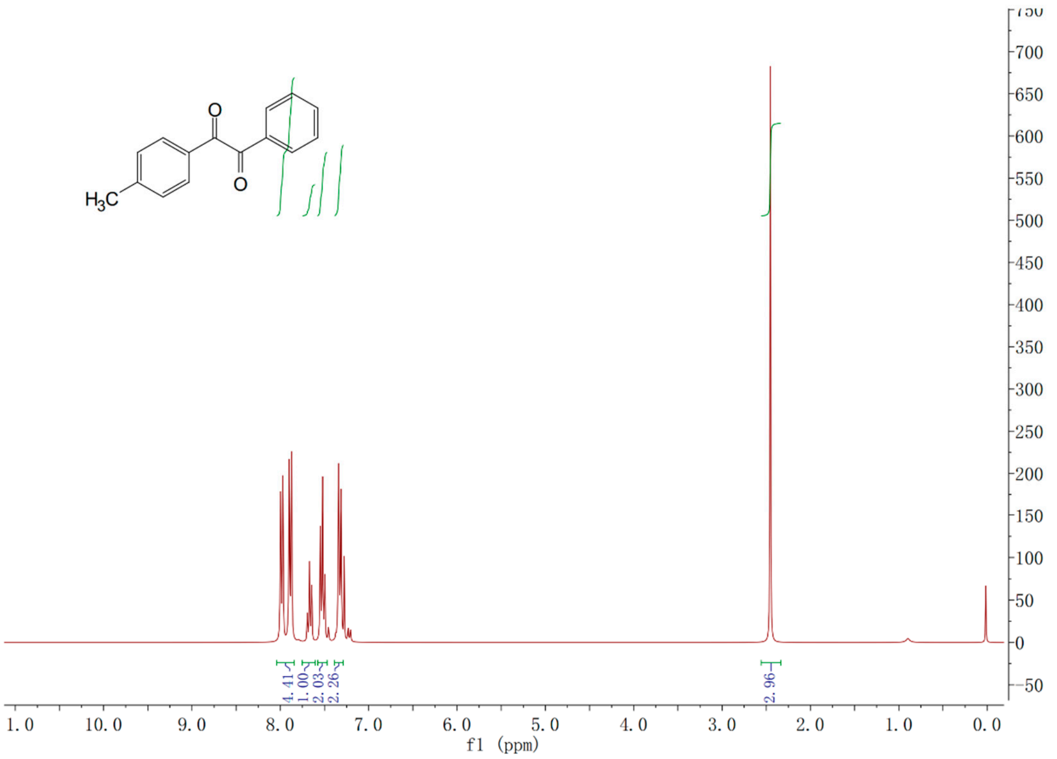

Figure S67. ^{1}H NMR spectrum of 2bg in $CDCl_3$ at room temperature

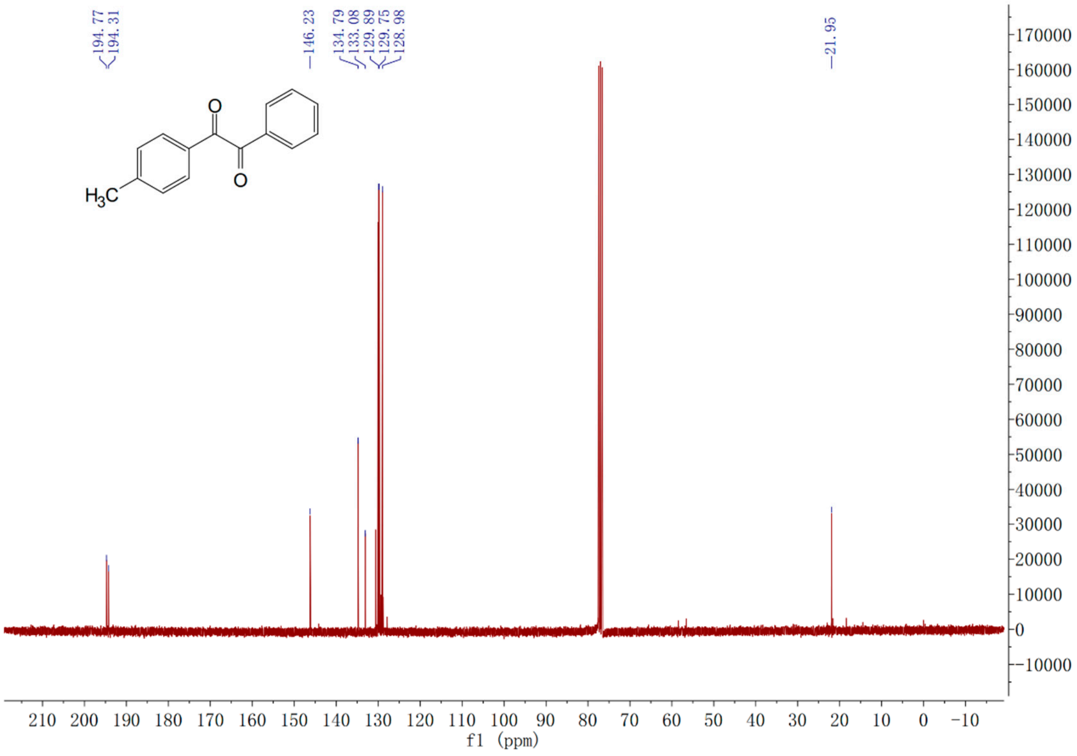

Figure S68. ^{13}C NMR spectrum of 2bg in $CDCl_3$ at room temperature

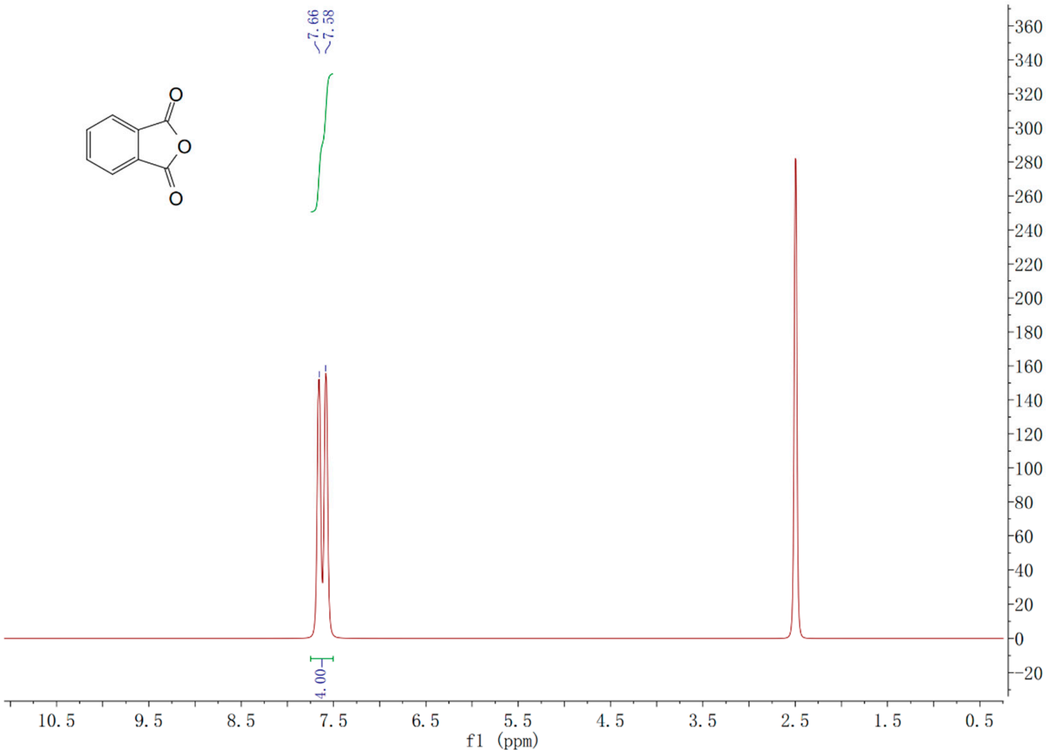

Figure S69. ^{1}H NMR spectrum of 2bh in DMSO at room temperature

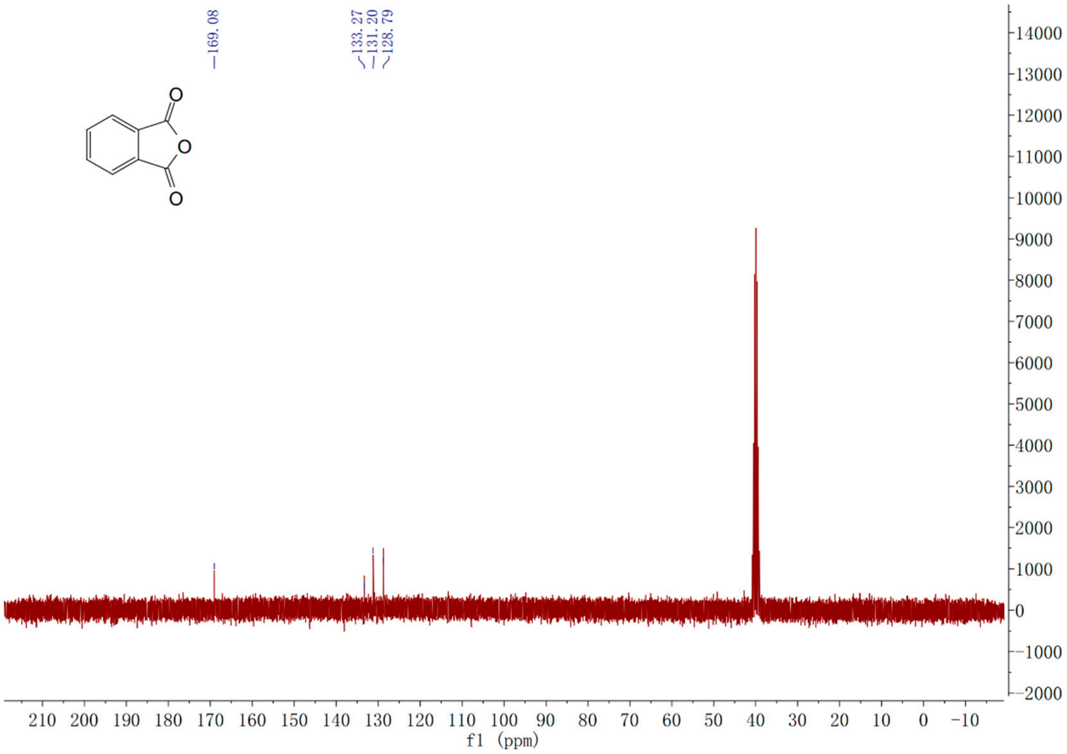

Figure S70. ^{13}C NMR spectrum of 2bh in DMSO at room temperature
